# A Practical Protocol for a Comprehensive Evaluation of Sulfur Fumigation of Trichosanthis Radix Based on Both Non-Targeted and Widely Targeted Metabolomics

**DOI:** 10.3389/fpls.2020.578086

**Published:** 2020-09-17

**Authors:** Chuanzhi Kang, Chaogeng Lv, Jian Yang, Liping Kang, Wenqi Ma, Wenjin Zhang, Sheng Wang, Tielin Wang, Jiahui Sun, Yang Ge, Lu-Qi Huang, Lanping Guo

**Affiliations:** National Resource Center for Chinese Materia Medica, China Academy of Chinese Medical Sciences, State Key Laboratory Breeding Base of Dao-di Herbs, Beijing, China

**Keywords:** sulfur-fumigation, Trichosanthis Radix, chemical markers, widely targeted metabolomics, transformation mechanism, non-targeted metabolomics

## Abstract

Trichosanthis Radix (TR) is one of the most severely sulfur-fumigated herbs in the market, whose transformation mechanism of chemical compositions and sulfur-fumigation markers of TR have not been clarified. To excavate characteristic sulfur-fumigation markers of TR samples, this study brings up a practical protocol using both ultra-performance liquid chromatography/quadrupole time-of-flight-mass spectrum (UPLC-ESI-QTOF-MS/MS)-based non-targeted metabolomics and ultra-performance liquid chromatography/electrospray ionization/quadrupole multiple-stage linear ion-trap mass spectrum (UPLC-ESI-QTRAP-MS/MS)-based widely targeted metabolomics. The results of study demonstrated that five characteristic markers are sulfur-containing components, which were identified as *p*-Hydroxybenzyl hydrogen sulfite, cucurbitacin D sulfite I, cucurbitacin D sulfite II, cucurbitacin B sulfite I, and cucurbitacin B sulfite II, respectively. Additionally, cucurbitacin B and D were also filtered and identified as the characteristic sulfur-fumigation markers. Meanwhile, the different sulfur-fumigation extent of TR samples was tested by chemical transformations analysis and sulfur dioxide residues test. Further, 58.16% (139 of 239) of the differential metabolites content significantly reduced in sulfur-fumigated TR samples. Besides, 20 kinds of non-sulfur marker metabolites were tested to evaluate the quality of TR samples before and after sulfur fumigation, predominantly including phenolic acids, amino acids, lipids and nucleotides. Taking TR as an example, this work provides a comprehensive practical protocol for the quality supervision of sulfur-fumigation herbs.

## Introduction

Sulfur fumigation has been used for more than 100 years as a common technology for the control of mold and insect in medicinal herbs (Jiang et al., 2013; Duan et al., 2016). This method was initially only used in a few Chinese herbs which are rich in starch and polysaccharides, such as Dioscoreae Rhizoma. Since the sulfur-fumigated herbs look better, weight more, and store easier than those that were not sulfur-fumigated. Sulfur fumigation was widely used in the initial processing of more variety of medicinal herbs including Gastrodiae Rhizoma (Kang et al., 2017), Achyranthis Bidentatae Radix (Kang et al., 2018), Angelicae Dahuricae Radix (Liu et al., 2014), Ginseng Radix (Zhu et al., 2015), and Paeoniae Radix Alba (Kong et al., 2018). Nevertheless, many studies have shown that sulfur-fumigation would cause residual sulfur dioxide in medicinal herbs, and would also cause quantitative and qualitative changes in their chemical components (Kong et al., 2014; Wu et al., 2018; Xing et al., 2018). Moreover, in order to reveal the quality, safety, and effectiveness of sulfur-fumigation, it is more meaningful to study the changes in the chemical composition of the Chinese herbal medicine after fumigation by comparing it with the residue of sulfur dioxide in Chinese herbal medicine. With the deepening of the research on sulfur-fumigation of medicinal herbs and the application of metabolomics-related technologies, growing numbers of sulfur-fumigation markers of medicinal herbs have been tapped. Previous studies have revealed that some special chemical components in medicinal herbs could be sulfited or sulfated to produce new sulfur-containing markers after sulfur-fumigation, such as the main saponin components in Ginseng Radix (Zhu et al., 2015), the main coumarin and its glycosides in Angelicae Dahuricae Radix (Liu et al., 2014), the flavonoid glycosides in Pueraria Lobata Radix (Yang et al., 2015), the phenylethanol glycosides in Gastrodiae Rhizoma (Kang et al., 2017), the cycloolefin ether terpenes in Lonicerae Japonicae Flos (Guo et al., 2014), and the monosaccharide in Moutan Cortex (Zhan et al., 2018). Therefore, it is assumed that the effect of sulfur-fumigation on the chemical composition of medicinal herbs of the same composition type may reveal the same transformation mechanism. The critical steps in the quality control of medical materials include exploring the chemical transformation mechanism of sulfur-fumigated herbs and excavating the stable and reliable sulfur-fumigation markers.

Trichosanthis Radix (TR) is one of the most vital herbs and frequently used for promoting fluid relieving thirst, clearing heat and fire, swelling and evacuating pus (National Pharmacopoeia Committee, 2015b). The main bioactive compounds of TR are trichosanthin, polysaccharides, saponins, starches, and proteins. To be specific, saponins (such as cucurbitacin B and cucurbitacin D, etc.) have effects of antitumor, anti-inflammatory, antimicrobial, antispasmodic, antidiabetic, and immunomodulatory (Li et al., 2003; Li et al., 2010; Chen et al., 2014). However, the study on the sulfur-fumigation of TR was still very insufficient as there were no clear quality control indicators (active ingredients). The reported studies were mainly focused on the detection of sulfur dioxide residues and the total contents of a single type of ingredients such as the total protein, total polysaccharides, total saponins, etc. (Zheng et al., 2016). So far, there still a lack of systematic research on the quantitative and qualitative changes of the chemical composition of sulfur fumigated TR. Moreover, there are currently neither reports on the formation of new sulfur-fumigation markers during the sulfur fumigation process of TR nor reports on the regulation of the chemical conversion of different levels of sulfur fumigation. At present, non-targeted metabolomics and targeted metabolomics based on LC-QTOF-MS or LC-QTRAP-MS have become more mature in the analysis of the chemical composition of Chinese herbal medicines and the mining of quality control indicators (Ma et al., 2014; Li et al., 2016; Dai et al., 2018; Wei et al., 2018; Zhang et al., 2018; Shengyun et al., 2019; Jiang et al., 2020). Specifically, broadly targeted metabolomics is a detection technology that integrates the “extensiveness” of non-targeted metabolomics with the “accuracy” of targeted metabolomics (Luo et al., 2018; Zhu et al., 2018). With the self-built, secondary database and the multiple reaction monitoring (MRM) scanning mode, it could identify metabolites qualitatively and quantitatively the samples batch by batch and finally obtain more concrete and more accurate metabolite information of the sample. QTOF-based non-targeted metabolomics can perform metabolic profile analysis on the chemical components of medicinal herbs. On this basis, QTRAP-based targeted metabolomics can perform qualitative and quantitative analysis of specific components in combination with established compound databases.

In this study, firstly, UPLC-ESI-QTOF-MS/MS-based non-targeted metabolomics technology was used to mine the sulfur fumigation markers of TR, and these markers were tentatively identified by the fragmentation characteristics of mass spectrometry. Second, the sulfur dioxide residues and sulfur fumigation markers in 30 batches of TR samples collected on the market were evaluated to prove the universality and feasibility of the markers. Third, a widely targeted metabolomics analysis based on UPLC-ESI-QTRAP-MS/MS was performed focusing on the key chemical components, including key sulfur-fumigation markers and some main components in TR, to clarify the transformation mechanism of chemical constituents of TR with different sulfur-fumigation levels. Finally, based on non-targeted metabolomics and broadly targeted metabolomics, this paper established a plan for a sulfur fumigation quality evaluation system, which can provide an important reference for the quality and safety evaluation of TR and other similar herbal materials.

## Materials and Methods

### Chemicals, Reagents, and Herbal Materials

Acetonitrile and formic acid (HPLC grade) were purchased from Merck (Darmstadt, GER). Analytic grade methanol was purchased from Fisher Scientific (Hudson, NH, USA). Deionized water was purified using a Milli-Q system (Millipore, MA, USA).

Standard compounds of cucurbitacin B (NO. 18052116), cucurbitacin D (NO. 19042168), and cucurbitacin E (NO. 19032512) were purchased from Shanghai Shifeng Bio-Technology Co., Ltd (Shanghai, China). Lead acetate (NO. 20160914), hydrochloric acid (NO. 20171107), soluble starch (NO. 20170414), iodine (NO. 20160914), and potassium iodide (NO. 20170110) were obtained from Sinopharm Chemical Reagent Co., Ltd (Shanghai, China). *p*-Hydroxybenzyl hydrogen sulfite was prepared in our lab and confirmed by HR-MS and NMR analyses (Kang et al., 2017).

Fresh TR sample was collected from Shexian (Henan, China), the traditional “Dao Di” producing area, in November, 2018. 30 batches of commercial TR samples were collected from Bozhou material medicine market. All collected samples were identified as *Trichosanthes kirilowii* or *Trichosanthes rosthornii* by Prof. Lan-Ping Guo. The authenticated specimens were deposited in the National Resource Center for Chinese Materia Medica, China Academy of Chinese Medical Sciences.

### Sulfur Fumigation of TR

The sulfur fumigation of TR was performed as described in the previous study (Kang et al., 2017). Briefly, a plastic apparatus comes apart into upper and lower layers at first, and then the skinless TR samples and sulfur was separately placed in the upper section and the lower section of the apparatus according to the weight ratio of sulfur to herbal material of 1:40 which was adopted to simulate the sulfur-fumigation conditions used by farmers. The time of sulfur-fumigation was set to 1, 2, and 4 h, respectively. Finally, the samples were dried at 45 °C and ground into powder (MM 400, Retsch, Germany). All samples were prepared in three biological replicates and stored at 4 °C prior to analysis.

### Preparation of Sample and Standard Solutions

#### Sample Preparation for UPLC-QTOF-MS/MS

One hundred and fifty micrograms TR powder were extracted by ultrasonication with 1.5 ml 80%(v/v) methanol for 60 min, then the extracted solution was centrifuged for 10 min at 13,000 rpm, and finally, the supernatant was filtered through a 0.2-μm microporous membrane filter before UPLC-QTOF/MS analysis. The mixed extract solutions were used as a control for quality control (QC).

#### Sample Preparation for UPLC-ESI-QTRAP-MS/MS

One hundred micrograms TR sample powder was extracted with 0.6 ml 70% methanol. The extract solution was then centrifugated at 10,000*g* for 10 min, absorbed by an SPE Cartridge (CNWBOND Carbon-GCB, 250 mg, 3 ml, Shanghai, China) and was filtrated through a 0.2-μm microporous membrane filter before UPLC-MS/MS analysis. The mixed extract solutions were used as a control sample for quality control.

### Sulfur Dioxide Residue Analysis

The sulfur dioxide residue was determined by iodine titration according to the Chinese Pharmacopoeia 2015 version (Part four) Appendix 2331 (National Pharmacopoeia Committee, 2015a).

### UPLC-ESI-QTOF-MS/MS Analysis

#### UPLC-ESI-QTOF-MS Conditions

UPLC analysis of the TR sample was performed by Waters Acquity UPLC-I-Class system (Waters Corporation, Milford, MA, US) coupled with Acquity HSS T3 column (100 × 2.1 mm, 1.8 μm) for chromatographic separation. The column temperature was 40°C, and the flow rate was 0.5 ml/min. The mobile phases were 0.1% formic acid aqueous solution (A) and acetonitrile containing 0.1% formic acid (B). The gradient elution program was as follows: 5%→12% B (0–0.3 min), 12→17% B (0.3–4 min), 17→23% B (4–5 min), 23→36% B (5–10 min), 36%→38% B (10–11 min), 38→42% B (11–12.5 min), 42→51% B (12.5–15 min), 51%→57% B (15–19 min), 57→62% B (19–21.5 min), 62→80% B (21.5–23.5 min), 80→98% B (23.5–25 min), 98% B(25–27 min), 98→5% B (27–27.5 min), 5% B (27.5–30 min). The injection volume was 3 μl.

MS analysis was performed by using a Waters Xevo G2-S QTOF-MS equipped with electrospray ionization (ESI) source in negative ionization mode. The MS data acquisition mode was the MS^E^ continuum. The desolvation gas flow rate was 900 L/h. The source temperature was 100°C. The desolvation temperature was 450 °C. The data acquisition range was 50 to 1,500 Da. The collision energy was 45 to 70 eV; the capillary voltage was 2 kV; the cone voltage was 40 V. The ions [M-H]^−^ (m/z 554.2620) of leucine enkephalin (200 pg/μl, 10 μl/min) was used as lock spray for mass accuracy.

### Multivariate Statistical Analysis

The MassLynx^™^ software and Progenesis QI software (Waters Co., Milford, MA, USA) were used to dissect the potential characteristic compounds of sulfur-fumigated TR based on retention time and accurate mass (Kang et al., 2017; Kang et al., 2018). The MS ions were aligned by Progenesis QI with a retention time window of 0.20 min and a mass tolerance of 5.0 ppm. Then, differential compounds were filtered with ANOVE *p*-value (*p* ≤ 0.05), minimum coefficient of variation (the value ≥ 2), and max fold change (the value ≥ 2). Finally, the principal component analysis (PCA) and orthogonal partial least squared discriminant analysis (OPLS-DA) were performed by EZinfo software 3.0 (Version 3.0; Waters Co., Milford, MA) and SIMCA-P software (Version 14.1; Umetrics, Umea, Sweden) (Lyu et al., 2020).

### UPLC-ESI-QTRAP-MS/MS Analysis

#### UPLC-ESI-QTRAP-MS/MS Conditions

The chromatographic separation of TR extracts was analyzed by UPLC-ESI-MS/MS system (UPLC, Shim-pack UFLC SHIMADZU CBM30A system) coupled with Acquity HSS T3 column (100 × 2.1 mm, 1.8 μm). The column temperature was 40 °C and the flow rate was 0.5 ml/min. The mobile phases were 0.04% acetic acid aqueous solution (A) and acetonitrile with 0.04% acetic acid (B). The gradient elution program was as follows: 5%→95% B (0–10 min), 95% B (10–11 min).95→5% B (11–11.1 min), 5% B (11.1–14 min). The injection volume was 4 μl.

MS analysis was performed by a triple quadrupole-linear ion trap mass spectrometer system (API 4500 QTRAP UPLC/MS/MS) equipped with an ESI Turbo Ion-Spray interface in both positive and negative ionization mode. Analyst 1.6.3 software (AB Sciex, Boston, US) was chosen to analyze the data, whose parameters were as follows: the ion spray voltage of positive ion mode (IS) was 5500 V and negative ion mode was −4500 V. The source temperature was set at 550°C. The collision gas (CAD) was high. The ion source curtain gas (CUR), gas I (GSI), and gas II (GSII) were set at 50, 60, and 30.0 psi, respectively. The collision gas (nitrogen)was set as 5 psi. QQQ scans were performed using Multiple Reaction Monitoring (MRM).

### Multivariate Statistical Analysis

The data was unit variance scaled and then a PCA was performed by statistics function prcomp in R (www.r-project.org). The hierarchical cluster analysis (HCA) results were demonstrated as heatmaps with dendrograms and the Pearson correlation coefficients (PCC) were calculated by cor function and presented as heatmaps by pheatmap in R. The score plots and permutation plots of OPLS-DA were generated by MetaboAnalystR in R. The differential metabolites were filtered by VIP ≥ 1 and absolute Log2FC (fold change) ≥ 1. The permutation test was performed to avoid overfitting and the parameter was set 200 permutations.

## Results and Discussion

### Optimization of UPLC-QTOF-MS Conditions for TR

The evaluation of different extraction solvents (methanol, ethanol, 80% ethanol, 50% methanol, 80% methanol) were carried out to find the most satisfactory extraction efficiency and integrity. Finally, a concentration of 80% methanol was selected (**Figure S1**).

Both ESI (−) and ESI (+) ion data were acquired in the study and finally, the ESI (−) was chosen considering the higher peak capacity and better resolution of the chromatogram (**Figure S1**). Additionally, more literature has shown that the ion data of sulfur-fumigation markers of medicinal herbs were mainly detected in negative mode (Kang et al., 2017; Kang et al., 2018). Hence, the differential metabolites analysis of sulfur-fumigation TR mostly uses mass spectrometry data in negative ion mode.

#### Non-Targeted Metabolomics Analysis of Sulfur-Fumigated TR With UPLC-QTOF-MS/MS

##### Non-targeted Metabolomics Analysis for Exploring Sulfur-fumigation Markers**

**Figure 1:**
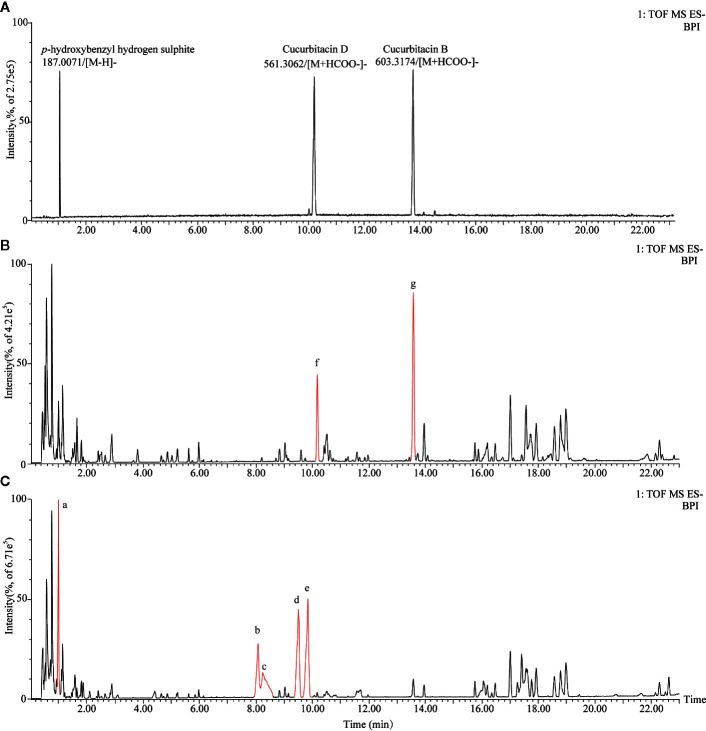
Total ion chromatograms of TR extraction for sulfur-fumigated prior to fumigation and after 1 h in negative mode. **(A)** Total ion chromatograms of *p*-Hydroxybenzyl hydrogen sulfite, cucurbitacin B, and cucurbitacin D. **(B)** Total ion chromatograms of non-fumigated TR extraction. **(C)** Total ion chromatograms of sulfur-fumigated TR extraction; Compounds a and g are sulfur-fumigation markers in TR samples.

The characteristic metabolites of non-fumigated (0 h) and sulfur-fumigated (1 h) TR samples were analyzed respectively (**Figure 1**). The result showed that some components of TR changed obviously after sulfur-fumigation, such as components a-g. To be specific, the response intensity of compounds f and g reduced apparently, whereas compounds a-e enhanced during the sulfur-fumigation. Furthermore, compound a was tentatively identified as *p*-Hydroxybenzyl hydrogen sulphite, compared to reference substances. Compounds f and g were identified as cucurbitin D and cucurbitin B, respectively (**Figure 1A** **)**, and compounds b-e were more likely to be the conversion products of them.

**Figure 2:**
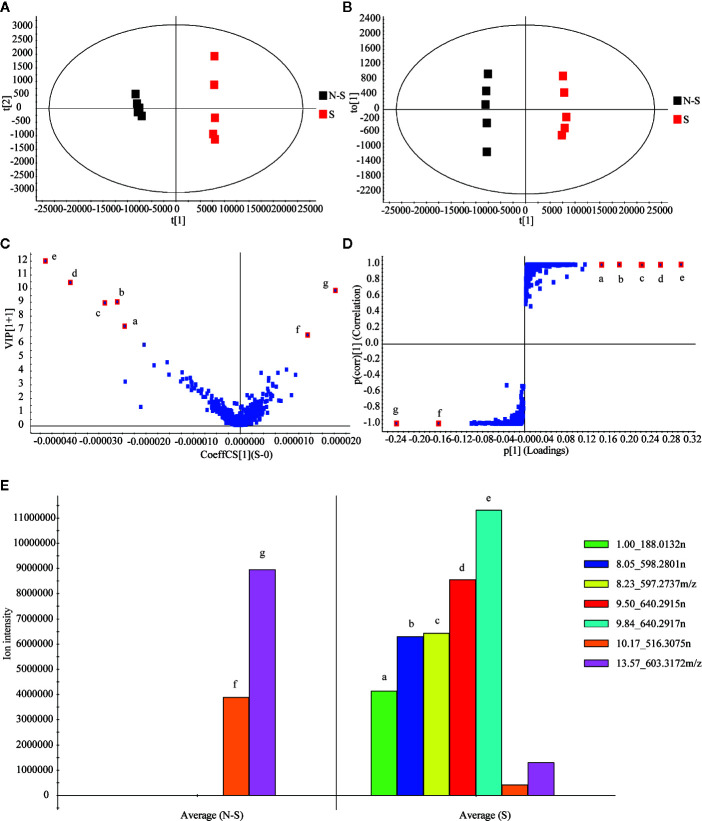
Multivariate statistical analysis of methanol extracts of non-fumigated TR (0 h) and sulfur-fumigated GR (2 h) samples: **(A)** PCA/scores plot (five samples with each group, three biological replicates); **(B)** OPLS-DA/scores plot (five samples with each group, three biological replicates); **(C)** VIP plot; **(D)** S-plot; **(E)** column plot of the ion intensity (compounds a–g are significantly different markers in TR samples).

Then, MassLynx software was used to obtain the MS data within 23 min, which was aligned by Progenesis QI software. After the removal of the background (Lin et al., 2015), 2,787 ions of all samples were obtained and aligned. Subsequently, 1648 ions with statistical significance were filtered based on the parameters of ANOVA *p*-value (*p* ≤ 0.05), minimum coefficient of variation (the value ≥ 2), and max fold change (the value ≥ 2) (Kang et al., 2017). Later, EZinfo software was operated to analyze the PCA, whose score plots showed a clearly differential trend between the non-fumigated and sulfur-fumigated samples, and OPLS-DA analysis of 1648 ions (**Figure 2A**). Next, the OPLS-DA model (R^2^X = 0.969, Q^2^ = 0.998) was established to identify potential markers (**Figure 2B**). The permutations number of Cross-Validation was set to 200 and all blue Q^2^-values were lower than the original points (**Figure S2**). Ultimately, at the bottom right of the VIP plot (VIP > 6.0), seven potential marker ions including **a** (t_R_ 1.00, m/z 187.0059), **b** (t_R_ 8.05, m/z 597.2736), **c** (t_R_ 8.23, m/z 595.2737), **d** (t_R_ 9.50, m/z 639.2842), **e** (t_R_ 9.84, m/z 639.2844), **f** (t_R_ 10.17, m/z 561.3066) and **g** (t_R_ 13.57, m/z 603.3172) and the S-plot (**Figures 2C, D** **)** were selected. Among all the objects of the test, the ions **a**-**e** were only detected in sulfur-fumigated TR samples, and the intensity of ions **f** and **g** in non-fumigated samples were higher than sulfur-fumigated samples (**Figure 2E**).

**Figure 3:**
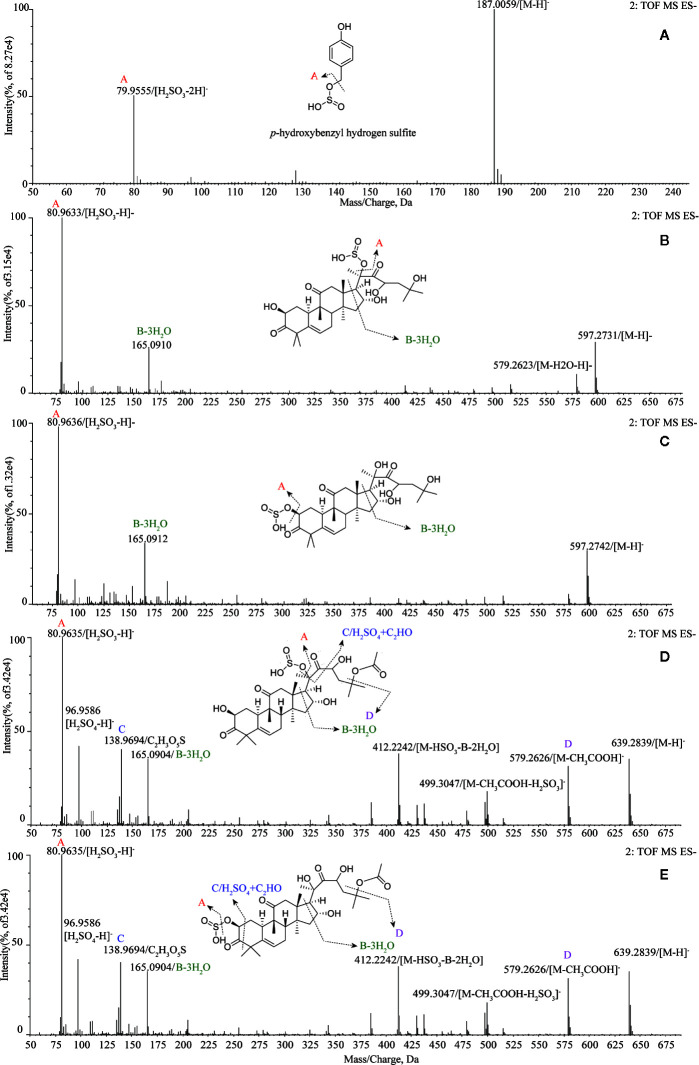
Structural analysis of five sulfur-fumigation markers for TR. **(A)** pyrolysis debris of *p*-Hydroxybenzyl hydrogen sulfite; **(B)** pyrolysis debris of cucurbitacin D sulfiteI; **(C)** pyrolysis debris of cucurbitacin D sulfiteII; **(D)** pyrolysis debris of cucurbitacin B sulfiteI; **(E)** pyrolysis debris of cucurbitacin B sulfiteII.

Tentatively, the chemical structures and fragmentation regularities of these seven markers were clarified by retention times and secondary cleavage diagram (Zha et al., 2016). At 1.00 min, the fragment ion of marker **a** (*m/z* 187.0059) was detected and identified as quasi-molecular [M-H]^-^. And there was an obvious characteristic fragment of the sulfonic group (SO_3_, m/z 79.9555) in the structure of the marker without any other fragment ions (**Figure 3A**). In addition, referring to the previous study (Kang et al., 2017), this marker was also found in the study of the sulfur-fumigated Gastrodia Rhizoma, whose molecular ion peak and fragmentation law are consistent. Consequently, marker **a** was tentatively identified as *p*-Hydroxybenzyl hydrogen sulfite. Next, from the accurate *m*/*z* and MS/MS information, marker **b** and **c**, **d** and **e** were identified as isomers respectively, and these four markers were supposed to be newly generated compounds with *m/z* 80.9635 ([H_2_SO_3_-H]^-^) after sulfur-fumigation (**Figure 3** and **Table 1**). By comparison with reference substance, marker **f** was identified as cucurbitacin D and **g** was identified as cucurbitin B (**Figure 1**). Correspondingly, the markers **b** and **c** are assumed to be sulfur-containing derivatives of cucurbitacin D, while **d** and **e** are considered to be sulfur-containing derivatives of cucurbitacin B. The fragment ions information of cucurbitacin B and cucurbitacin D showed that they both underwent sulfonation and addition reactions during sulfur-fumigation. Then, the binding position of sulfonic was deduced according to previous studies (Zhang et al., 2012; Zhu et al., 2015) and the specific structure and fragmentation regularities of markers a-e were finally determined with clog*P* value (**b** (clog*P* = 0.98), **c** (clog*P* = 1.15), **d** (clog*P* = 1.93) and **e** (clog*P* = 2.10)) and fragment ions. (see **Figure 3**).

**Table 1:** Seven sulfur-fumigation markers of TR.

Primary ID	Retention time (min)	Expected mass	m/z	Error	Formula	Identification	VIP	Factor of Change	MS/MS
a	1.00	188.0143	187.0059	−0.6	C_7_H_8_O_4_S	*p*-Hydroxybenzyl hydrogen sulfite	7.27	4566.4	187.0059, 128.0336, 79.9555
b	8.05	598.2811	597.2736	−0.2	C_30_H_46_O_10_S	Cucurbitacin D sulfiteI	8.96	6293.0	597.2731, 579.2623, 165.0910, 80.9633, 79.9553
c	8.23	598.2811	597.2737	0.9	C_30_H_46_O_10_S	Cucurbitacin D sulfiteII	9.04	6428.9	597.2742, 165.0912, 80.9636
d	9.50	640.2917	639.2842	−0.2	C_32_H_48_O_11_S	Cucurbitacin B sulfiteI	10.44	8547.3	639.2837, 579.2636, 412.2239, 165.0910, 138.9695, 96.9588, 80.9636
e	9.84	640.2917	639.2844	0.0	C_32_H4_8_O_11_S	Cucurbitacin B sulfiteII	12.02	11311.6	639.2839, 579.2626, 412.2242, 165.0904, 138.9694, 96.9586, 80.9635
f	10.17	516.3087	561.3066	2.7	C_30_H_44_O_7_	Cucurbitacin D	6.63	9.2	561.3066[M+HCOO]^−^, 551.2720, 491.1284, 325.1779, 165.0903, 137.0969, 96.9679
g	13.57	558.3192	603.3172	−2.1	C_32_H_46_O_8_	Cucurbitacin B	9.86	6.9	603.3148[M+HCOO]^−^, 527.1032, 491.1276, 295.2265, 165.0906, 116.9281

**Figure 4:**
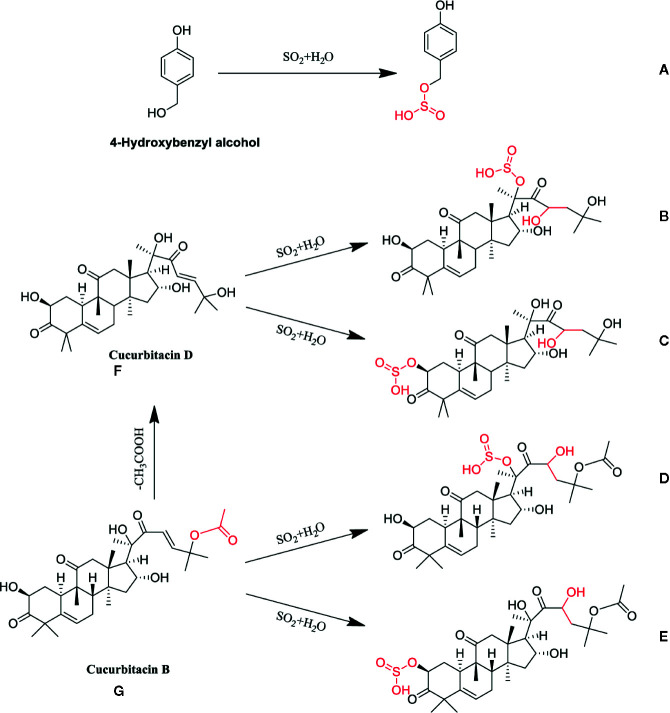
Possible chemical structure changes of the markers in TR during sulfur fumigation. **(A)** p-Hydroxybenzyl hydrogen sulfite; **(B)** Cucurbitacin D sulfite I; **(C)** Cucurbitacin D sulfite II; **(D)** Cucurbitacin B sulfite I; **(E)** Cucurbitacin B sulfite II; **(F)** Cucurbitacin D, **(G)**: Cucurbitacin B.

Moreover, markers **b**-**e** were further verified whether they were transformed from cucurbitin B and D after sulfur-fumigation. The products of sulfur-fumigated cucurbitin B and cucurbitin D were detected, and the results showed that cucurbitacin D converted into markers **b** and **c** *via* sulfonation (**Figure S3A**) and cucurbitin B undergoes a sulfonation reaction to generate markers **d** and **e** after sulfur-fumigation. Besides, a hydrolysis reaction also occurred in cucurbitin D. Eventually, the paper gives the whole transformation mechanism of *p*-Hydroxybenzyl alcohol, cucurbiten B, cucurbiten D, and markers **a**-**e** in the sulfur-fumigation process (**Figure 4**).

### Identification of Commercial TR Samples Using Sulfur Fumigation Markers

Then, the five sulfur-containing markers (**a**-**e**) were adopted to the identification of sulfur-fumigation in commercial TR samples. 30 batches of commercial TR samples collected from market were tested for sulfur dioxide residues and it was found that 13.3% (4 batches out of 30 batches) of the tested samples were fumigated. Nonetheless, while choosing markers for verification at the same time, markers **a-e** were detected in 11 batches of samples, indicating that the percentage of sulfur fumigated samples (36.7%) was much higher than the results of the sulfur dioxide residue test. This kind of situation might root in the instability of sulfur dioxide residues in sulfur-fumigated medicinal materials, which could be affected by storage time, processing methods, and so on. In addition, marker **a** was not detected in the sample No. 22 (**Figure S4** and **Table S1**). This may be due to the low content of *p*-Hydroxybenzyl alcohol in the sample No. 22 primarily, which led to a lower conversion rate of maker **a** after sulfur-fumigation. Interestingly, although sample No. 30 has a sulfur dioxide residue of 31.50 mg/kg after detection, but no marker detected, indicating that this batch of samples was not actually sulfur fumigated. It is supposed that the reason may be that the sulfur fumigation of this batch of TR samples is so insufficient that the peel could not be penetrated and preserve its chemical composition. Another possibility is that there is an operational error in the sulfur dioxide detection process. **(** **Table S1** **)**. Even though sulfur-fumigation has been banned in the processing of medicinal herbs, from a practical point of view, the sulfur-fumigation problem of changing the chemical composition and efficacy of TR still exist. Above all, these results indicated that a more scientific and reasonable evaluation of the quality of medicinal herbs affected by sulfur-fumigation needs a combination of both external sulfur dioxide test and internal sulfur-fumigation markers detection.

### Dynamic Monitor Five Major Markers During the Sulfur Fumigation Process of TR Samples

**Figure 5:**
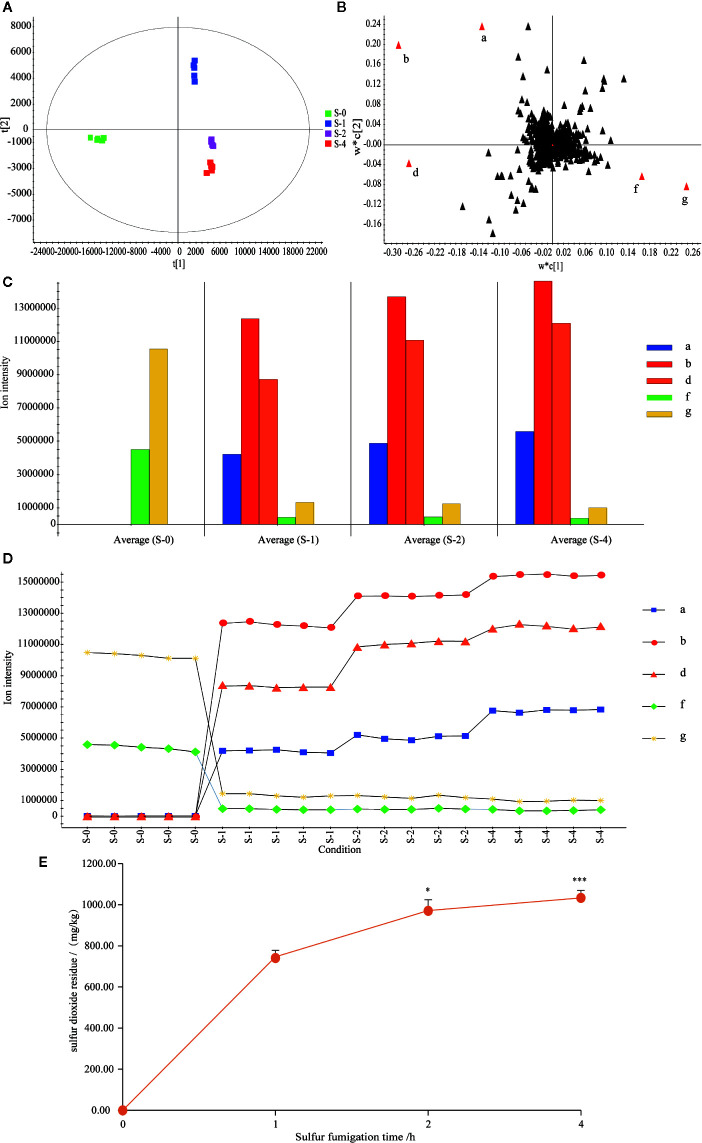
Content variations of the five markers and sulfur dioxide residue in the TR samples within 4 h sulfur-fumigation. **(A)** PCA/scores plot; **(B)** Loading score; **(C)** column plot of the ion intensity; **(D)** ion intensity variations of the five markers in 4 h; **(E)** content variations of sulfur dioxide residue in sulfur-fumigation process. ***p<0.001, compared with 1 h; *p<0.05, compared with the 1 h.

To dissect the chemical transformation mechanisms of sulfur-fumigation markers, the five major markers of sulfur-fumigated TR samples were divided into different groups (sulfur-fumigated with 0, 1, 2, and 4 h) were simultaneously determined. Moreover, the extent of sulfur-fumigation TR was evaluated by PCA and Loading plot analysis (**Figure 5**). The PCA plot (**Figure 5A**) revealed that the S-0 group was designed as a single group. The chemical composition of TR changed significantly between S-1 and S-4, and the change mainly occurred at the first 1 h after sulfur-fumigation and gradually weakened during the subsequent fumigation time. Specifically, as is shown in **Figure 5B**, the markers **a**, **b**, **d**, **f,** and **g** showed a large contribution to the differences among the four groups.

From **Figures 5C, D**, it can be seen that when the sample was not sulfur-fumigated, only marker **f** (Cucurbitacin D) and marker **g** (Cucurbitacin B) were detected. After the sulfur-fumigation, markers **a** (*p*-Hydroxybenzyl hydrogen sulfite), **b** (Cucurbitacin D sulfite I), and **d** (Cucurbitacin B sulfite I) were produced. As the time of sulfur-fumigation grows, the response intensity of the three newly generated markers gradually increased, and the markers **f** and **g** significantly decreased at the same time. From the trend of sulfur dioxide residues (**Figure 5E**), with the rising of sulfur-fumigation time, the sulfur dioxide residue of TR gradually increased and the rise velocity obviously slowed after 2 h. At the 1, 2, and 4 h of sulfur-fumigation, the sulfur dioxide residue reached 747.04 mg/kg, 971.40 mg/kg (*P*<0.05, compared with 1 h) and 1033.13 mg/kg (*P*<0.001, compared with 1 h) respectively. The results above indicated that sulfur-containing markers of TR samples were mainly produced in the initial process of sulfur-fumigation. With the continuous increase of sulfur dioxide residues, the quality of TR has not been significantly affected (Kang et al., 2017). And with the continued increase of sulfur dioxide residues, the quality of TR was not significantly affected (Kang et al., 2017). Therefore, the sulfur fumigation time should be controlled less than 1 h, so that the impact of sulfur fumigation on the quality of herbal medicines can be minimized, and sulfur dioxide residues can be under the maximum risk limit (750 mg/kg) (Kang et al., 2017).

### Targeted Metabolomics Analysis of Sulfur-Fumigated TR With ESI-Q TRAP-MS/MS

#### ESI-QTRAP-MS/MS Analysis of Chemical Constituents of TR

To compare the differences of all detected metabolites in TR samples, the mass spectrum peaks of each metabolite were corrected based on the retention time and peak type information. (**Figures S5A, B**). And the data repeatability and reliability of the analysis of the chemical constituents of TR was verificated by overlaying and analyzing the total ion current (TIC) chromatograms of QC samples (**Figures S5C, D**). Furthermore, Pearson’s Correlation Coefficient was adopted as the evaluation index of biological repeated correlation. As shown in **Figure S6**, r^2^ was close to 1, indicating that the replicates were well correlated.

Analyst 1.6.3 and MultiaQuant software was operated to identify and analyze the chemical constituents of TR samples based on a local metabolic database. The TIC chromatograms and MRM metabolite detection multimodality (Multi-Substance Extraction Ion Chromatogram, XIC) was obtained in both negative and positive mode (**Figure S7**). Ultimately, a total of 426 metabolites were detected based on the UPLC- ESI-QTRAP-MS/MS platform and MWDB (metware database) from TR samples, including 70 amino acids and their derivatives, 67 phenolic acids, 38 nucleotides and their derivatives, 17 flavonoids, 15 lignin and coumarins, 29 alkaloids (including phenolamines, alkaloids, and indole alkaloids), 19 terpenes (including diterpenes, triterpenes, and triterpenoid saponins), 44 organic acids, 74 lipids (including sphingolipids, glycerides, free fatty acids, phosphatidylcholine (PC) lysophosphatidylcholine (LPC), lysophosphatidylethanolamine (LPE)), and 53 other types compounds such as vitamins, sugars, and alcohols. Among the metabolites mentioned above, the main ingredients of TR samples are amino acids, phenolic acids, lipids, and organic acids (**Table S2**).

### Dynamic Monitor the Overall Quality of the Sulfur Fumigation Process of TR Samples

**Figure 6:**
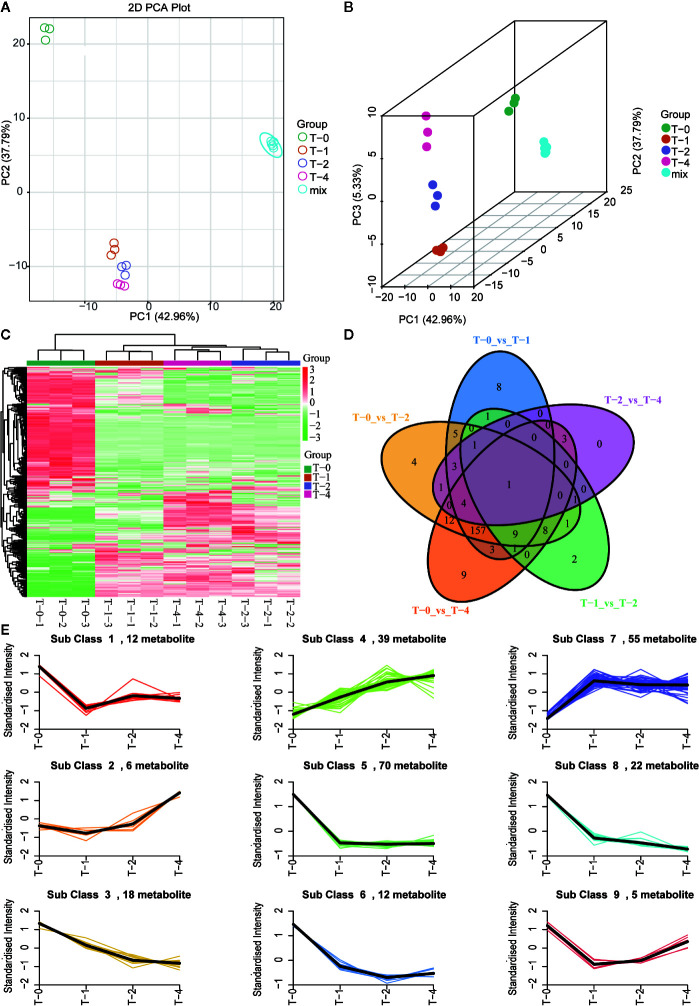
Multivariate statistical analysis of methanol extracts of TR samples in different sulfur-fumigation time. **(A)** PCA plot; **(B)** 3D OPLS-DA plot; **(C)** heatmap; **(D)** venn; **(E)** relative content trend of metabolites in different sulfur-fumigation time.

For dynamic monitoring, the overall quality changes of TR samples during the sulfur fumigation process, the 426 metabolites identified above were determined, and multivariate statistical analysis was performed. As is shown in **Figure 6**, the QC sample (mix) exhibited a tight clustering, indicating that the model has a high degree of reliability. Both the PCA score plots (**Figure 6A**) and 3D OPLS-DA plots (**Figure 6B**) showed a distinction among the samples with different sulfur-fumigation time. Specifically, compared to T-0 samples, group T-1, T-2, and T-4 clustered together which indicated that they were similar in chemical compositions. Meanwhile, among T-1, T-2, and T-4 samples, it is found that the metabolites of TR also changed significantly at different point of sulfur-fumigation times especially as shown in 3D OPLS-DA plots. Besides, a similar result was shown in the heatmap analysis (**Figure 6C**) using the normalized relative content data of metabolites with pheatmap of the R program. The pheatmap showed that T-0 samples were one cluster alone, and T-1, T-2, and T-4 samples were in another cluster. Moreover, T-1 samples were distinguished from the other two groups. These results indicated that the chemical composition of TR changed significantly after at least 1 h of sulfur-fumigation, and then the chemical composition changed continuously along with the sulfur-fumigation time. The Venn Diagram (**Figure 6D**) showed the relationships of the differences of metabolites between different sample groups. As a consequence, a total of 239 differential metabolites were detected in all treatment groups. There were 171 differential metabolites shared among groups T-0vsT-1, T-0vsT-2, and T-0vsT-4. The number of differential metabolites among sulfur-fumigated samples (T-1, T-2, and T-4) is relatively small, indicating that metabolites mainly changed within the first hour of sulfur-fumigation.

To study the general tendency of metabolites content changes in different sulfur fumigated TR samples, firstly, the data of relative contents of 239 differential metabolites were standardized and centralized. Then a K-means clustering analysis was performed to divide the differential metabolites into 9 categories. **Table S3** presents the specific information of each differential metabolite categories and **Figure 6E** shows that most of the intense changes of these metabolites happened within 1 h. After 1 h sulfur-fumigation, most categories of the metabolites decline dramatically, including categories 1, 2, 3, 5, 6, 8, and 9. However, the relative content of class 2 metabolites strangely increased at sulfur fumigated 2 and 4 h later. The rest of 39 metabolites in class 4 (mainly including amino acids, nucleotides, and their derivatives) and 55 metabolites (predominantly involving phenolic acids, amino acids, and their derivatives) in class 7 showed an increasing trend after sulfur-fumigation, and it is assumed that it was caused by the undergone hydrolysis or esterification reactions during fumigation. Statistically, 58.16% (139 of the 239 metabolites) of the differential metabolites content significantly reduced, and 41.84% of the metabolites content increased after sulfur-fumigation. Overall, these results indicated that the effect of sulfur-fumigation on the chemical constituents of TR was complex and multi-faceted.

### UPLC-ESI-QTRAP-MS/MS-Based Metabolome for the Exploration of Marker Metabolites

**Figure 7:**
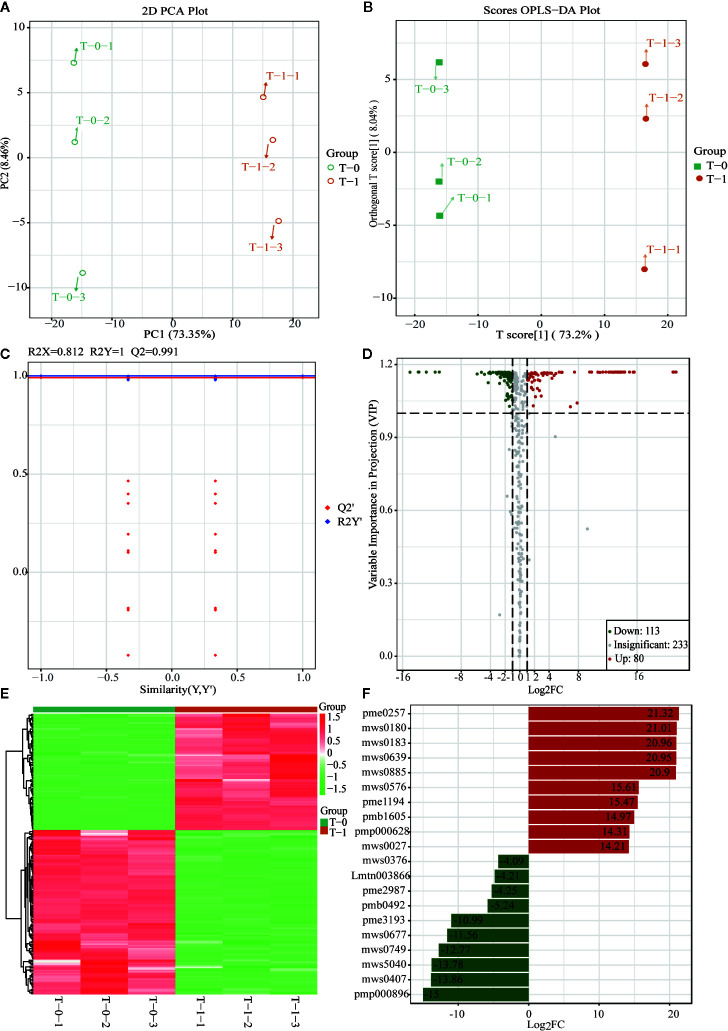
Multivariate statistical analysis of methanol extracts of non-fumigated TR (0 h) and sulfur-fumigated TR (1 h) samples. **(A)** PCA plot; **(B)** OPLS-DA plot; **(C)** validation Plot for OPLS-DA model; **(D)** volcano plot; **(E)** heatmap; **(F)** Multiple fold change of metabolites after log2 treatment.

An analysis was conducted based on the studies of global metabolites changes and difference analysis between samples with different sulfur-fumigation times mentioned above. The groups T-0 and T-1 were selected for the exploration of marker metabolites based on the data of UPLC**-**ESI-QTRAP-MS/MS. First of all, previous non-targeted metabolomics analysis demonstrated that the PCA and OPLS-DA demonstrated a clear difference between samples of T-0 and T-1 (**Figures 7A, B** **)**. The OPLS-DA model (R^2^X = 0.812, R^2^Y = 1, and Q^2^ = 0.991) was verified with the number of permutations of Cross-Validation set to 200 (Kang et al., 2017). In the model verification, R^2^Y ‘and Q^2^’ were both smaller than R^2^Y and Q^2^ of the original model (**Figure 7C**) which indicated that the model was feasible and the results could meet requirements. Subsequently, the ANOVA *p*-value (*p* ≤ 0.05), fold change (the value ≥ 2 or the value ≤ 0.5), and VIP ≥ 1 were performed to filter 193 marker metabolites with statistical significance (**Table S4**). As is shown in the volcano plot (**Figure 7D**), the green dots (including 113 metabolites) in the left mean down-regulated differential metabolites, while the red dots (including 80 metabolites) in the right represent up-regulated differential metabolites, and the grey dots in the middle stand for metabolites without significant difference. Then, after using the unit difference scaling and normalization, heat map analysis of these obviously different metabolite content data (**Figure 7E**) shows that there is a significant difference between the data of the T-0 and T-1 samples. Finally, according to fold change (log2FC) value, each top 10 differential marker metabolites were respectively screened from the increased and decreased metabolites as shown in **Figure 7F** and **Table 2**. These 20 markers were mainly phenolic acids, amino acids, lipids, and nucleotides. Notably, the content of uracil in TR most noticeably rose during the sulfur-fumigation process, and the content of cucurbitacin B and cucurbitacin D significantly declined during this process. Besides, *p*-Hydroxybenzyl alcohol was another significantly reduced metabolite, indicating that *p*-Hydroxybenzyl alcohol might be transformed to *p*-Hydroxybenzyl sulfite (marker **a**) by sulfonation (Kang et al., 2017). The results above were consistent with the three sulfur-fumigation markers *p*-Hydroxybenzyl hydrogen sulfite (marker **a**), cucurbitacin D (marker **f**), and cucurbitacin B (marker **g**) that was identified by non-targeted metabolomics with UPLC-QTOF-MS/MS from TR samples, which confirmed the accuracy and reliability of these sulfur-fumigation markers once again.

**Table 2:** 20 significant sulfur fumigation markers in TR.

NO.	Index	Formula	Name	Class I	Class II	VIP	Log2FC	Type	Substance identification level
1	pmp000896	C_32_H_46_O_8_	Cucurbitacin B	Terpenoids	Triterpene	1.17	−15.00	Down	B^a^
2	mws0407	C_30_H_44_O_7_	Cucurbitacin D	Terpenoids	Triterpene	1.17	−13.86	Down	B
3	mws5040	C_12_H_21_O_11_Na	Turanose	Others	Saccharides and Alcohols	1.17	−13.78	Down	B
4	mws0749	C_7_H_8_O_2_	4-Hydroxybenzyl alcohol	Phenolic acids	Phenolic acids	1.17	−12.77	Down	B
5	mws0677	C_12_H_14_N_2_O_2_	N-acetyl-5-hydroxytryptamine	Alkaloids	Alkaloids	1.17	−11.56	Down	B
6	pme3193	C_4_H_7_NO_3_	N-acetylglycine	Amino acids and derivatives	Amino acids and derivatives	1.17	−10.99	Down	A^b^
7	pmb0492	C_34_H_37_N_3_O_6_	N′,N″,N‴-p-Coumaroyl-cinnamoyl-caffeoyl spermidine	Alkaloids	Phenolamine	1.17	−5.24	Down	B
8	pme2987	C_9_H_8_O_2_	3,4-Dihydrocoumarin	Lignans and coumarins	Coumarins	1.17	−4.25	Down	B
9	Lmtn003866	C_9_H_8_O_2_	Trans-Cinnamic acid	Phenolic acids	Phenolic acids	1.17	−4.21	Down	B
10	mws0376	C_4_H_4_O_4_	Fumaric acid	Organic acids	Organic acids	1.17	−4.09	Down	A
11	mws0027	C_9_H_10_O_5_	Syringic acid	Phenolic acids	Phenolic acids	1.17	14.21	Up	A
12	pmp000628	C_15_H_20_N_2_O_2_	9α-Hydroxysophoramine	Alkaloids	Alkaloids	1.17	14.31	Up	B
13	pmb1605	C_21_H_36_O_4_	MAG(18:3)isomer3	Lipids	Glycerol ester	1.17	14.97	Up	A
14	pme1194	C_9_H_13_N_3_O_4_	Deoxycytidine	Nucleotides and derivatives	Nucleotides and derivatives	1.17	15.47	Up	B
15	mws0576	C_4_H_8_O_3_	3-Hydroxybutyrate	Organic acids	Organic acids	1.17	15.61	Up	B
16	mws0885	C_7_H_6_O_4_	2,4-Dihydroxy benzoic acid	Phenolic acids	Phenolic acids	1.17	20.90	Up	B
17	mws0639	C_7_H_6_O_4_	2,3-Dihydroxybenzoic Acid	Organic acids	Organic acids	1.17	20.95	Up	B
18	mws0183	C_7_H_6_O_4_	Protocatechuic acid	Flavonoids	Flavanols	1.17	20.96	Up	A
19	mws0180	C_7_H_6_O_4_	2,5-Dihydroxybenzoic acid	Phenolic acids	Phenolic acids	1.17	21.01	Up	A
20	pme0257	C_4_H_4_N_2_O_2_	Uracil	Nucleotides and derivatives	Nucleotides and derivatives	1.17	21.32	Up	A

B^a^: The parameters of Q1, Q3, RT, DP and CE of the substance are consistent with the database; A^b^: The second-level mass spectrometry and RT of the substance are consistent with the database.

In addition, it is found that the 14 terpenoids (cucurbitacin D, cucurbitacin B, cucurbitacin A, cucurbitacin F, etc.) in TR samples belonged to class 5, all of which showed a downward trend during sulfur-fumigation, as is shown in **Table S5** and **Figure 6E**. It is speculated that the active hydroxyl groups in this kind of metabolites can lead to sulfonation or esterification reactions. For instance, cucurbitoids can transform into derivatives products such as the sulfur-fumigation markers Cucurbitacin D sulfite I, Cucurbitacin D sulfite II, Cucurbitacin B sulfite I, and Cucurbitacin B sulfite II.

## Conclusion

In this study, a UPLC-QTOF-MS/MS-based non-targeted metabolomics combined with UPLC-QTRAP-MS/MS-based targeted metabolomics method was developed to identify characteristic sulfur-fumigated markers in TR samples. Consequently, based on the non-targeted metabolome combined with multivariate statistical analysis for the first time, seven fumigation markers were tentatively identified, including five sulfur-containing markers and two main component markers. Among them, four sulfur-containing components (Cucurbitacin D sulfite I, Cucurbitacin D sulfite II, Cucurbitacin B sulfite I, and Cucurbitacin B sulfite II) significantly accumulated in the first hour of sulfur-fumigation were highly correlated with the decrease of major constituent markers Cucurbitacin D and B. Besides, the maker *p*-Hydroxybenzyl hydrogen sulfite was obviously correlated with *p*-Hydroxybenzyl alcohol, and this marker is also present in sulfur-fumigated Gastrodia Rhizoma (Kang et al., 2017) which indicated that it has the potential to be developed as a relatively common sulfur-fumigation marker for other herbs. Further, based on the targeted metabolomics platform with a local database, a total of 426 metabolites in TR samples were detected and the chemical transformation mechanisms of 239 differential metabolites were dissected based on the relative quantitative analysis in the sulfur-fumigation process. Among them, the response intensity of 58.16% differential metabolites (139 out of the 239 metabolites), especially 14 terpenoids (Cucurbitacin D, Cucurbitacin B, Cucurbitacin A, Cucurbitacin F, etc.), significantly declined in sulfur-fumigated TR samples, which demonstrated that the chemical reaction of sulfur-fumigation to the terpenoids of TR presents the similar transformation mechanism. Moreover, during the sulfonation reaction process, the hydroxyl positions in terpenoids substituted by sulfite groups are basically the same, which has certain reference value for sulfur fumigants of the same chemical type. Furthermore, 20 marker metabolites, predominantly including phenolic acids, amino acids, lipids, and nucleotides, in non-fumigated (0 h) and sulfur-fumigated (1 h) TR were detected based on widely targeted metabolomics coupled with multivariate statistical analysis and the result was consistent with the non-targeted study. Hence, this study provides a practical solution for comprehensively assessing the quality control of sulfur-fumigated herbal medicines with characteristic chemical markers that combine non-targeted and targeted metabolomics methods.

## Data Availability Statement

The raw data supporting the conclusions of this article will be made available by the authors, without undue reservation.

## Author Contributions

CK performed data investigation and experiments. CL, JY, LK, WZ, SW, TW, JS, YG, and JL collected and organized the data. L-QH and LG designed this experiment. All authors contributed to the article and approved the submitted version.

## Funding

This work was supported by the National Key Research and Development Program of China (2017YFC1700701), National Natural Science Foundation of China (81891014), Ministry of Finance Central Level of the Special (2060302), Development and Reform Commission Standardization Project (ZYBZH-C-HLJ-17, ZYBZH-C-GD-07), Fundamental Research Funds for the Central public welfare research institutes (ZZ13-YQ-096, ZZXT201806).

## Conflict of Interest

The authors declare that the research was conducted in the absence of any commercial or financial relationships that could be construed as a potential conflict of interest.

The reviewer WX declared a past co-authorship with one of the authors with several of the authors L-QH, CK, WZ, SW, LG to the handling Editor.
