# Supplementary material for: A Practical Protocol for a Comprehensive Evaluation of Sulfur Fumigation of Trichosanthis Radix Based on Both Non-Targeted and Widely Targeted Metabolomics
Source: Front Plant Sci. 2020 Sep 17;11:578086. doi: 10.3389/fpls.2020.578086 (PMC7527402; doi:10.3389/fpls.2020.578086)
Supplement: Supplementary file 1 [file Table_1.docx]

# A practical protocol for a comprehensive evaluation of sulfur-fumigation of Trichosanthis Radix based on both non-targeted and widely targeted metabolomics

Chuanzhi Kang, Chaogeng Lv, Jian Yang, Liping Kang, Wenqi Ma, Wenjin Zhang, Sheng Wang, Tielin Wang, Jiahui Sun, Yang Ge, Lu-Qi Huang^*^, Lanping Guo^*^

National Resource Center for Chinese Materia Medica, China Academy of Chinese Medical Sciences, State Key Laboratory Breeding Base of Dao-di Herbs, Beijng, 100700, PR China

*Corresponding authors.

E-mail addresses: huangluqi01@126.com (L-Q. Huang), glp01@126.com (L. Guo).

**
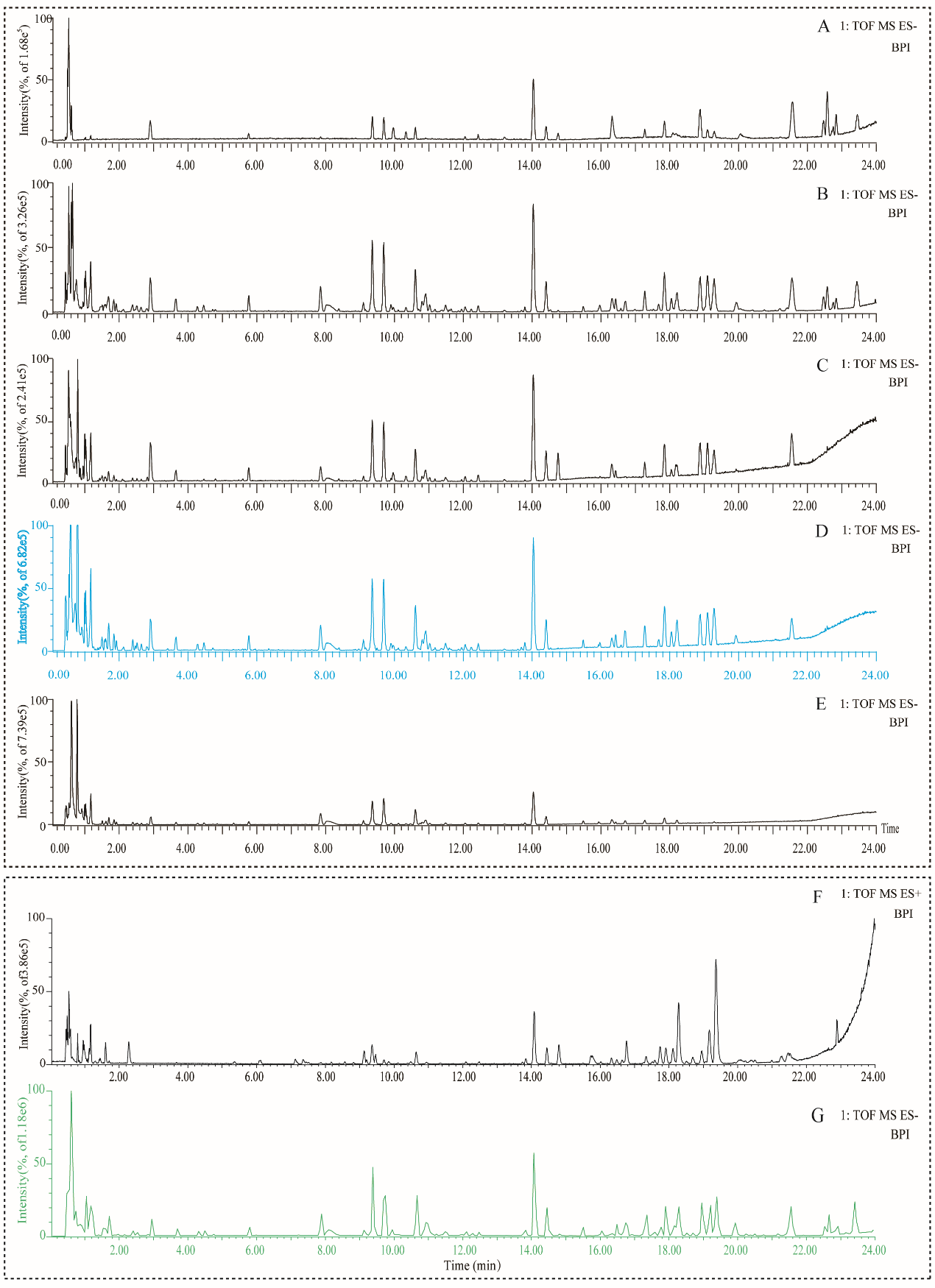
**

**FIGURE S1** Optimized extraction conditions selected from four extraction solvents and two analysis mode with ESI (‒) and ESI (+) ion data. A: ethanol; B: 80% ethanol; C: methanol; D: 80% methanol; E: 50% methanol; F: positive mode; G: negative mode


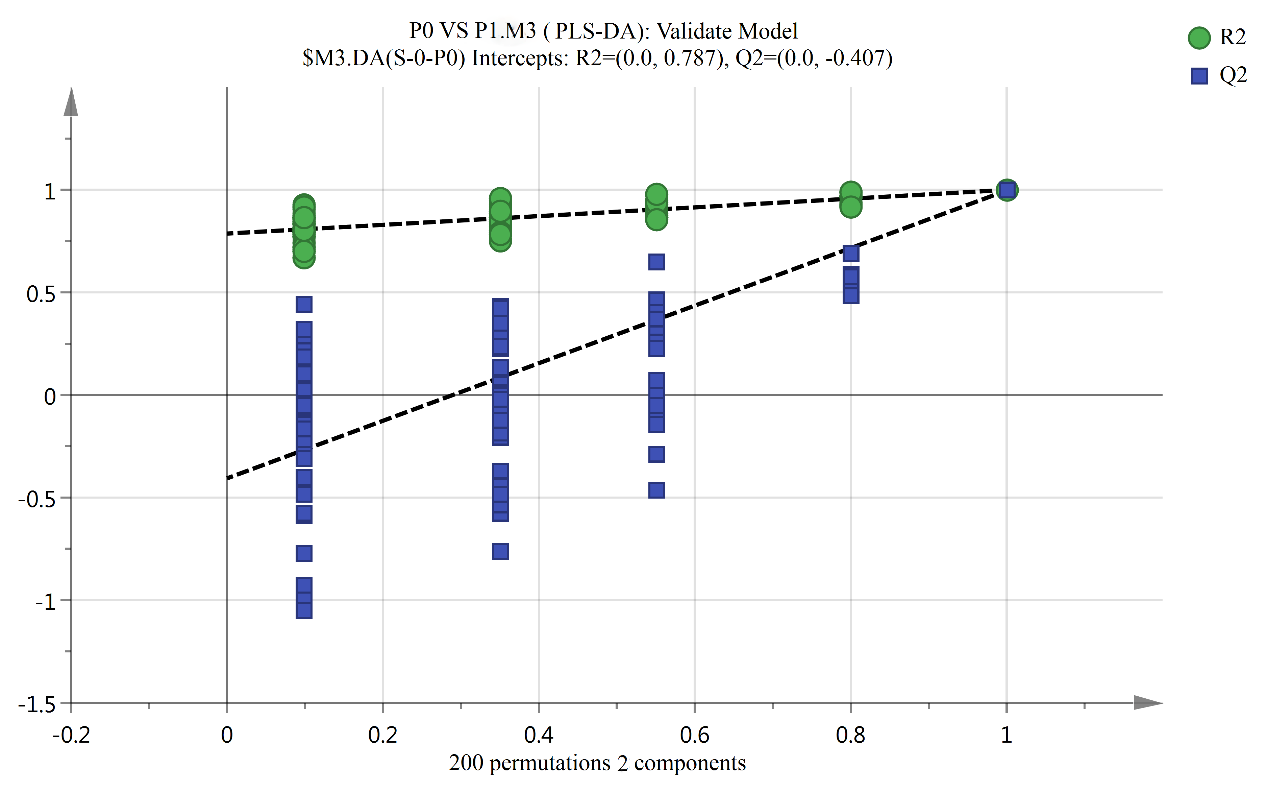


**FIGURE S2** Validation Plot for OPLS-DA Models. (All blue Q^2^-values to the left are lower than the original points to the right.)


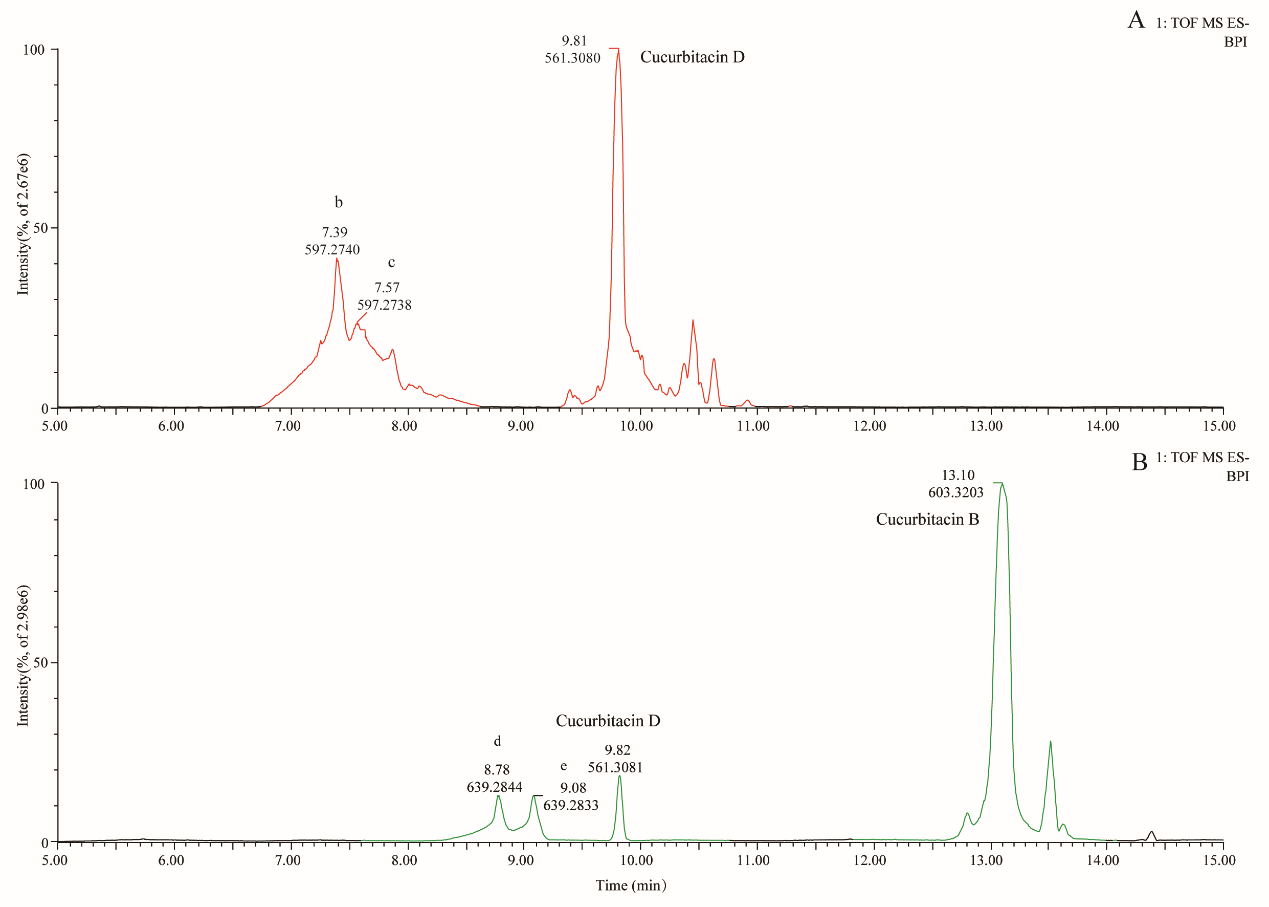


**FIGURE S3** Structural transformation of cucurbitacin B and cucurbitacin D standards after sulfur-fumigation. (A: cucurbitacin B; B: cucurbitacin D; b, c, d and e are sulfur-fumigation markers in TR samples.)


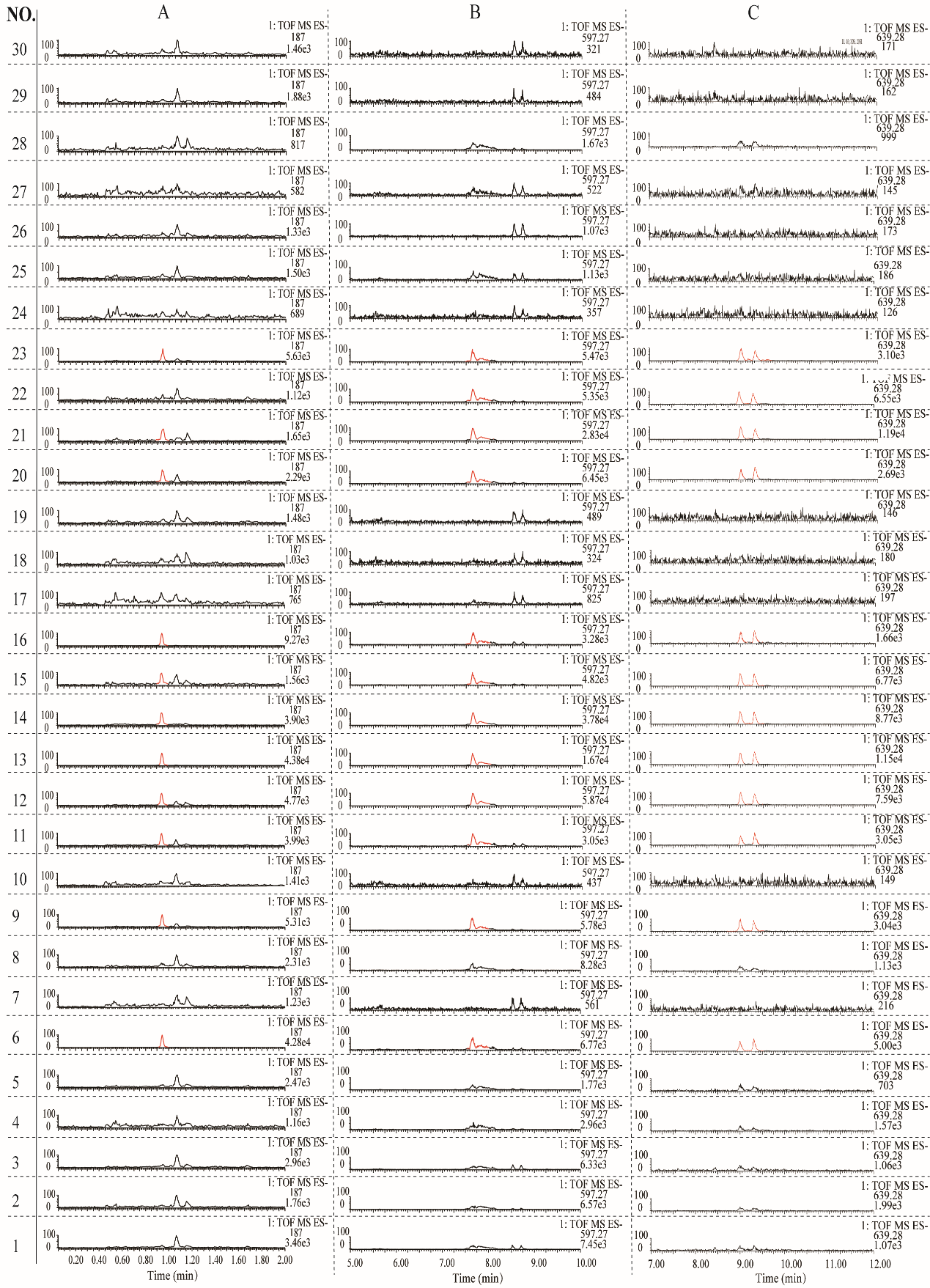


**FIGURE S4** EIC chromatograms of thirty batches of commercial TR samples. (A: marker a, m/z 187.0059; B: marker b and c, m/z 597.2736; C: marker d and e, m/z 639.2842)


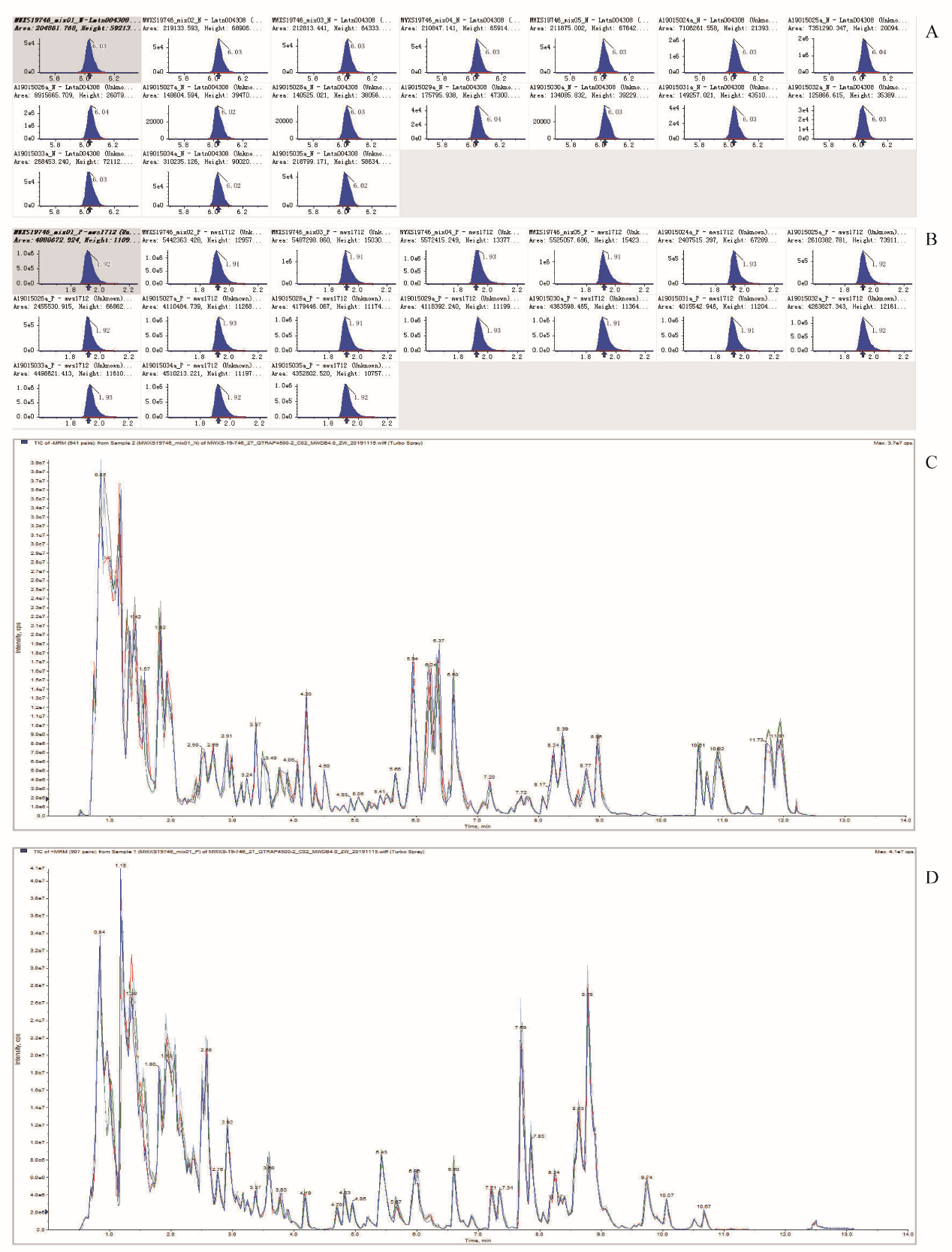


**FIGURE S5** Quality control (QC) sample verification of TR. (A: Integral correction chart for quantitative analysis of metabolites in negative ion mode; B: Integral correction chart for quantitative analysis of metabolites in positive ion mode; C: Overlay of TIC in negative ion mode of QC sample; D: Overlay of TIC in positive ion mode of QC sample.)


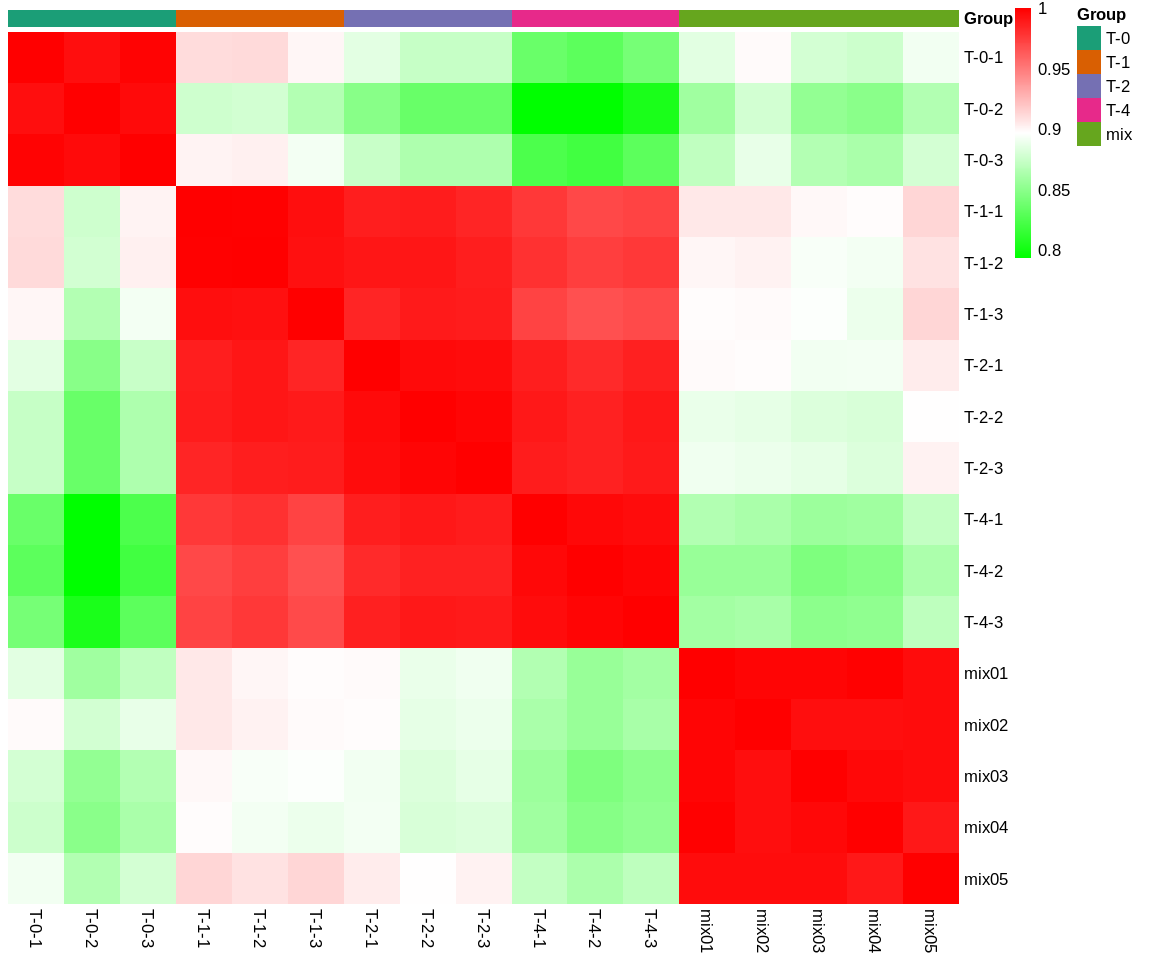


**FIGURE S6** Correlation heatmap of sulfur-fumigation samples. (T-0: non-fumigated sample; T-1: sulfur-fumigated 1 hour; T-2: sulfur-fumigated 2 hours; T-4: sulfur-fumigated 4 hours.)


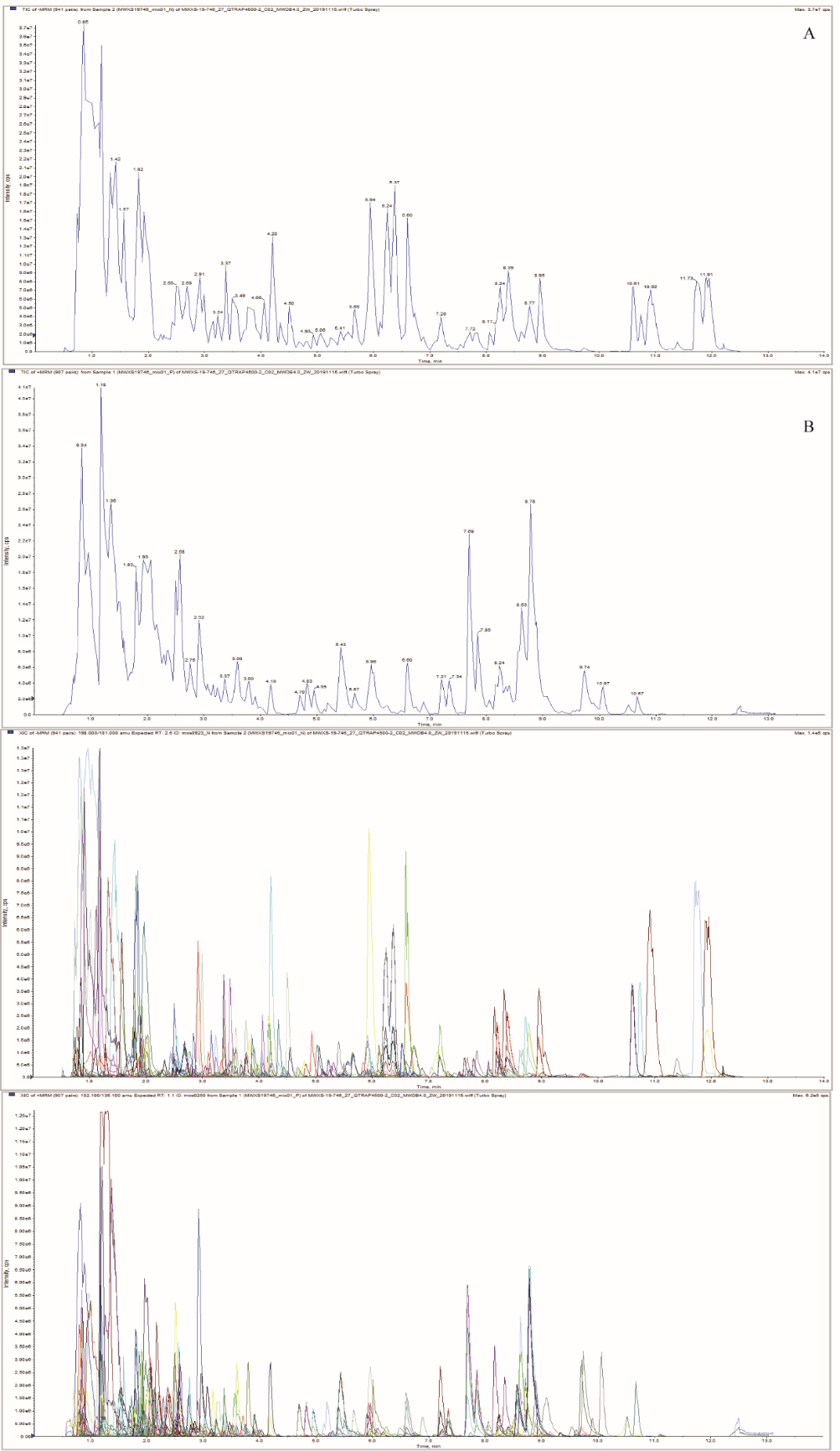


**FIGURE S7** MRM chromatograms of mix samples of TR extraction. (A: mixed sample total ion chromatogram in negative ion mode; B: Mixed sample total ion chromatogram in positive ion mode; C: multimodality plots for MRM metabolite detection in negative ion mode; D: multimodality plots for MRM metabolite detection in negative ion mode.)

**Table S1** sulfur-fumigation markers detection of thirty batches of commercial TR samples.

| Code number | Origin | Sulfur dioxide residues（mg/kg） | Marker a | Marker b | Marker c | Marker d | Marker e | Marker f | Marker g |
| --- | --- | --- | --- | --- | --- | --- | --- | --- | --- |
| 1 | Bozhou market, Anhui Province | - | - | - | - | - | - | + | + |
| 2 | Bozhou market, Anhui Province | - | - | - | - | - | - | + | + |
| 3 | Bozhou market, Anhui Province | - | - | - | - | - | - | + | + |
| 4 | Bozhou market, Anhui Province | - | - | - | - | - | - | + | + |
| 5 | Bozhou market, Anhui Province | - | - | - | - | - | - | + | + |
| 6 | Bozhou market, Anhui Province | - | + | + | + | + | + | + | + |
| 7 | Bozhou market, Anhui Province | - | - | - | - | - | - | + | + |
| 8 | Bozhou market, Anhui Province | - | - | - | - | - | - | + | + |
| 9 | Bozhou market, Anhui Province | - | + | + | + | + | + | + | + |
| 10 | Bozhou market, Anhui Province | - | - | - | - | - | - | + | + |
| 11 | Bozhou market, Anhui Province | - | + | + | + | + | + | + | + |
| 12 | Bozhou market, Anhui Province | - | + | + | + | + | + | + | + |
| 13 | Bozhou market, Anhui Province | - | + | + | + | + | + | + | + |
| 14 | Bozhou market, Anhui Province | 57.71±3.53 | + | + | + | + | + | + | + |
| 15 | Bozhou market, Anhui Province | - | + | + | + | + | + | + | + |
| 16 | Anguo market, Hebei Province | 563.31±19.38 | + | + | + | + | + | + | + |
| 17 | Anguo market, Hebei Province | - | - | - | - | - | - | + | + |
| 18 | Anguo market, Hebei Province | - | - | - | - | - | - | + | + |
| 19 | Anguo market, Hebei Province | - | - | - | - | - | - | + | + |
| 20 | Anguo market, Hebei Province | - | + | + | + | + | + | + | + |
| 21 | Anguo market, Hebei Province | - | + | + | + | + | + | + | + |
| 22 | Anguo market, Hebei Province | - | — | + | + | + | + | + | + |
| 23 | Anguo market, Hebei Province | 369.37±21.33 | + | + | + | + | + | + | + |
| 24 | Anguo market, Hebei Province | - | - | - | - | - | - | + | + |
| 25 | Anguo market, Hebei Province | - | - | - | - | - | - | + | + |
| 26 | Anguo market, Hebei Province | - | - | - | - | - | - | + | + |
| 27 | Anguo market, Hebei Province | - | - | - | - | - | - | + | + |
| 28 | Anguo market, Hebei Province | - | - | - | - | - | - | + | + |
| 29 | Anguo market, Hebei Province | - | - | - | - | - | - | + | + |
| 30 | Anguo market, Hebei Province | 31.50±0.79 | - | - | - | - | - | + | + |

- Not detected.

**Table S2** 426 chemical components of TR samples identified by ESI-Q TRAP-MS/MS.

| Code | Index | Q1 (Da) | Molecular Weight (Da) | Formula | Ionization model | Compounds | Class I | Class II | Level |
| --- | --- | --- | --- | --- | --- | --- | --- | --- | --- |
| 1 | Xigua_cub013 | 457.37 | 456.33 | C_30_H_48_O_3_ | [M+H]+ | 11-Carbonyl-20β-hydroxycucurbitadienol | Terpenoids | Triterpene | A^a^ |
| 2 | Xigua_mab0299 | 677.36 | 678.32 | C_36_H_54_O_12_ | [M-H]- | Dihydroisocucurbitacin I-Hex | Terpenoids | Triterpene Saponin | A |
| 3 | Xigua_mab0310 | 677.36 | 678.32 | C_36_H_54_O_12_ | [M-H]- | Cucurbitacin D O-glucoside | Terpenoids | Triterpene Saponin | A |
| 4 | Xigua_mab0406 | 719.37 | 720.33 | C_38_H_56_O_13_ | [M-H]- | Cucurbitacin D-aceGlu | Terpenoids | Triterpene Saponin | A |
| 5 | Xigua_mab0535 | 473.36 | 472.32 | C_30_H_48_O_4_ | [M+H]+ | 11-Carbonyl-2β,20β-dihydroxycucurbitadienol | Terpenoids | Triterpene | A |
| 6 | Xigua_mab0543 | 719.37 | 720.33 | C_38_H_56_O_13_ | [M-H]- | 23,24-Dihydro cucurbitacin E O-glucoside | Terpenoids | Triterpene Saponin | A |
| 7 | mws0407 | 515.30 | 516.28 | C_30_H_44_O_7_ | [M-H]- | Cucurbitacin D | Terpenoids | Triterpene | A |
| 8 | mws1629 | 345.20 | 346.11 | C_15_H_22_O_9_ | [M-H]- | Aucubin | Terpenoids | Sesquiterpenoids | A |
| 9 | mws4017 | 285.22 | 286.21 | C_20_H_30_O | [M-H]- | Ferruginol | Terpenoids | Ditepenoids | A |
| 10 | Xigua_cub060 | 557.31 | 558.29 | C_32_H_46_O_8_ | [M-H]- | 23,24-Dihydro cucurbitacin E | Terpenoids | Triterpene | B^b^ |
| 11 | pmn001429 | 573.18 | 574.28 | C_32_H_46_O_9_ | [M-H]- | Cucurbitacin A | Terpenoids | Triterpene | B |
| 12 | pmn001433 | 517.20 | 518.29 | C_30_H_46_O_7_ | [M-H]- | Cucurbitacin F | Terpenoids | Triterpene | B |
| 13 | pmn001705 | 471.35 | 472.32 | C_30_H_48_O_4_ | [M-H]- | 3,24-Dihydroxy-17,21-semiacetal-12(13)oleanolic fruit | Terpenoids | Triterpene | B |
| 14 | pmp000270 | 501.32 | 500.28 | C_30_H_44_O_6_ | [M+H]+ | Lup-12-en-15α,19β-diol-3,11-dioxo-28-oic acid | Terpenoids | Triterpene | B |
| 15 | pmp000438 | 443.39 | 442.35 | C_30_H_50_O_2_ | [M+H]+ | Betulin | Terpenoids | Triterpene | B |
| 16 | pmp000896 | 1134.66 | 558.29 | C_32_H_46_O_8_ | [2M+NH4]+ | Isocucurbitacin B | Terpenoids | Triterpene | B |
| 17 | pmp000898 | 663.37 | 662.33 | C_36_H_54_O_11_ | [M+H]+ | Deacetoxycucurbitacin B 3-O-glucoside | Terpenoids | Triterpene Saponin | B |
| 18 | pmp000899 | 681.37 | 680.34 | C_36_H_56_O_12_ | [M+H]+ | Cucurbitacin F O-glucoside | Terpenoids | Triterpene Saponin | B |
| 19 | pmp000901 | 721.38 | 720.33 | C_38_H_56_O_13_ | [M+H]+ | Isocucurbitacin B 2-O-glucoside | Terpenoids | Triterpene Saponin | B |
| 20 | Hmjp006575 | 677.37 | 676.36 | C_33_H_56_O_14_ | [M+H]+ | Glyceryl linoleate-O-Hexoside-O-Hexoside 1 | Lipids | Glycerol ester | A |
| 21 | Qingke_Rfmb089-2-3 | 313.24 | 314.22 | C_18_H_34_O_4_ | [M-H]- | 9,10-Dihydroxy-12-octadecenoic acid | Lipids | Free fatty acids | A |
| 22 | Qingke_Rfmb090-1-3 | 295.23 | 296.21 | C_18_H_32_O_3_ | [M-H]- | 13-Hydroxy-9,11-octadecadienoic acid | Lipids | Free fatty acids | A |
| 23 | Qingke_Rfmb091-1-1 | 295.23 | 296.21 | C_18_H_32_O_3_ | [M-H]- | 9-Hydroxy-10,12-octadecadienoic acid | Lipids | Free fatty acids | A |
| 24 | mws0119 | 227.20 | 228.19 | C_14_H_28_O_2_ | [M-H]- | Myristic Acid | Lipids | Free fatty acids | A |
| 25 | mws0126 | 524.37 | 523.33 | C_26_H_54_NO_7_P | [M+H]+ | 1-Stearoyl-sn-glycero-3-phosphocholine | Lipids | LPC | A |
| 26 | mws0289 | 478.10 | 479.27 | C_23_H_46_NO_7_P | [M-H]- | LysoPE 18:1 | Lipids | LPE | A |
| 27 | mws0359 | 241.22 | 242.21 | C_15_H_30_O_2_ | [M-H]- | Pentadecanoic Acid | Lipids | Free fatty acids | A |
| 28 | mws0361 | 253.22 | 254.21 | C_16_H_30_O_2_ | [M-H]- | Palmitoleic Acid | Lipids | Free fatty acids | A |
| 29 | mws0366 | 277.22 | 278.21 | C_18_H_30_O_2_ | [M-H]- | γ-Linolenic Acid | Lipids | Free fatty acids | A |
| 30 | mws0367 | 277.10 | 278.21 | C_18_H_30_O_2_ | [M-H]- | α-Linolenic Acid | Lipids | Free fatty acids | A |
| 31 | mws0383 | 267.23 | 268.22 | C_17_H_32_O_2_ | [M-H]- | Cis-10-Heptadecenoic Acid | Lipids | Free fatty acids | A |
| 32 | mws0396 | 281.25 | 282.23 | C_18_H_34_O_2_ | [M-H]- | Elaidic Acid | Lipids | Free fatty acids | A |
| 33 | mws0752 | 185.16 | 186.15 | C_11_H_22_O_2_ | [M-H]- | Undecylic Acid | Lipids | Free fatty acids | A |
| 34 | mws1489 | 283.26 | 284.25 | C_18_H_36_O_2_ | [M-H]- | Stearic Acid | Lipids | Free fatty acids | A |
| 35 | mws2623 | 281.25 | 282.23 | C_18_H_34_O_2_ | [M-H]- | 11-Octadecanoic acid(Vaccenic acid) | Lipids | Free fatty acids | A |
| 36 | mws5045 | 215.17 | 216.16 | C_12_H_24_O_3_ | [M-H]- | 12-Hydroxydodecanoic acid | Lipids | Free fatty acids | A |
| 37 | pmb0160 | 353.30 | 352.24 | C_21_H_36_O_4_ | [M+H]+ | MAG(18:3)isomer5 | Lipids | Glycerol ester | A |
| 38 | pmb0164 | 355.10 | 354.25 | C_21_H_38_O_4_ | [M+H]+ | MAG(18:2)isomer1 | Lipids | Glycerol ester | A |
| 39 | pmb0296 | 357.20 | 356.27 | C_21_H_40_O_4_ | [M+H]+ | MAG(18:1)isomer2 | Lipids | Glycerol ester | A |
| 40 | pmb0854 | 518.30 | 517.28 | C_26_H_48_NO_7_P | [M+H]+ | LysoPC 18:3 | Lipids | LPC | A |
| 41 | pmb0863 | 492.30 | 491.27 | C_24_H_46_NO_7_P | [M+H]+ | LysoPC 16:2(2n isomer) | Lipids | LPC | A |
| 42 | pmb0864 | 426.30 | 425.22 | C_19_H_40_NO_7_P | [M+H]+ | LysoPE 14:0 | Lipids | LPE | A |
| 43 | pmb0865 | 518.20 | 517.28 | C_26_H_48_NO_7_P | [M+H]+ | LysoPC 18:3(2n isomer) | Lipids | LPC | A |
| 44 | pmb0874 | 478.60 | 477.25 | C_23_H_44_NO_7_P | [M+H]+ | LysoPE 18:2(2n isomer) | Lipids | LPE | A |
| 45 | pmb0876 | 454.20 | 453.25 | C_21_H_44_NO_7_P | [M+H]+ | LysoPE 16:0 | Lipids | LPE | A |
| 46 | pmb0889 | 279.30 | 278.21 | C_18_H_30_O_2_ | [M+H]+ | Punicic acid | Lipids | Free fatty acids | A |
| 47 | pmb1562 | 351.00 | 350.22 | C_21_H_34_O_4_ | [M+H]+ | MAG(18:4)isomer3 | Lipids | Glycerol ester | A |
| 48 | pmb1605 | 353.30 | 352.24 | C_21_H_36_O_4_ | [M+H]+ | MAG(18:3)isomer3 | Lipids | Glycerol ester | A |
| 49 | pmb2221 | 318.30 | 317.27 | C_18_H_39_NO_3_ | [M+H]+ | 4-Hydroxysphinganine | Lipids | Sphingolipids | A |
| 50 | pmb2260 | 480.31 | 479.27 | C_23_H_46_NO_7_P | [M+H]+ | LysoPC 15:1 | Lipids | LPC | A |
| 51 | pmb2319 | 482.30 | 481.28 | C_23_H_48_NO_7_P | [M+H]+ | LysoPC 15:0 | Lipids | LPC | A |
| 52 | pmb2325 | 353.10 | 352.24 | C_21_H_36_O_4_ | [M+H]+ | MAG(18:3)isomer2 | Lipids | Glycerol ester | A |
| 53 | pmb2363 | 357.20 | 356.27 | C_21_H_40_O_4_ | [M+H]+ | MAG(18:1)isomer1 | Lipids | Glycerol ester | A |
| 54 | pmb2406 | 510.10 | 509.31 | C_25_H_52_NO_7_P | [M+H]+ | LysoPC 17:0 | Lipids | LPC | A |
| 55 | pmb2640 | 199.00 | 200.16 | C_12_H_24_O_2_ | [M-H]- | Lauric acid | Lipids | Free fatty acids | A |
| 56 | pmb2778 | 295.00 | 296.21 | C_18_H_32_O_3_ | [M-H]- | 9,10-EODE | Lipids | Free fatty acids | A |
| 57 | pmb2784 | 293.20 | 294.20 | C_18_H_30_O_3_ | [M-H]- | 13-HOTrE | Lipids | Free fatty acids | A |
| 58 | pmb2786 | 293.00 | 294.20 | C_18_H_30_O_3_ | [M-H]- | 9-HOTrE | Lipids | Free fatty acids | A |
| 59 | pmb2787 | 293.20 | 294.20 | C_18_H_30_O_3_ | [M-H]- | 9-KODE | Lipids | Free fatty acids | A |
| 60 | pmb2792 | 293.00 | 294.20 | C_18_H_30_O_3_ | [M-H]- | 13-HOTrE(r) | Lipids | Free fatty acids | A |
| 61 | pmb2799 | 295.00 | 296.21 | C_18_H_32_O_3_ | [M-H]- | 12,13-EODE | Lipids | Free fatty acids | A |
| 62 | pmd0130 | 468.30 | 467.27 | C_22_H_46_NO_7_P | [M+H]+ | LysoPC 14:0(2n isomer) | Lipids | LPC | A |
| 63 | pmd0132 | 496.33 | 495.30 | C_24_H_50_NO_7_P | [M+H]+ | LysoPC 16:0(2n isomer) | Lipids | LPC | A |
| 64 | pmd0136 | 524.36 | 523.33 | C_26_H_54_NO_7_P | [M+H]+ | LysoPC 18:0 | Lipids | LPC | A |
| 65 | pmd0160 | 452.30 | 453.25 | C_21_H_44_NO_7_P | [M-H]- | LysoPE 16:0(2n isomer) | Lipids | LPE | A |
| 66 | pmn001495 | 329.20 | 330.25 | C_19_H_38_O_4_ | [M-H]- | Hexadecanoic acid 2,3-dihydroxypropyl ester | Lipids | Glycerol ester | A |
| 67 | pmn001688 | 295.23 | 296.21 | C_18_H_32_O_3_ | [M-H]- | 9S-Hyroxy-10E,12E-octadecadienoic acid | Lipids | Free fatty acids | A |
| 68 | pmn001689 | 311.22 | 312.21 | C_18_H_32_O_4_ | [M-H]- | 9-Hydroxy-12-oxo-10-octadecenoic acid | Lipids | Free fatty acids | A |
| 69 | pmn001691 | 327.22 | 328.20 | C_18_H_32_O_5_ | [M-H]- | 9,12,13-Trihyroxy-10,15-octadecadienoic acid | Lipids | Free fatty acids | A |
| 70 | pmn001694 | 329.23 | 330.22 | C_18_H_34_O_5_ | [M-H]- | 9,10,13-Trihyroxy-11-octadecadienoic acid | Lipids | Free fatty acids | A |
| 71 | pmp001250 | 520.34 | 520.30 | C_26_H_51_NO_7_P+ | [M]+ | PC(18:2) | Lipids | PC | A |
| 72 | pmp001251 | 520.34 | 520.30 | C_26_H_51_NO_7_P+ | [M]+ | PC(18:2)isomer | Lipids | PC | A |
| 73 | pmp001264 | 274.28 | 273.24 | C_16_H_35_NO_2_ | [M+H]+ | Hexadecylsphingosine | Lipids | Sphingolipids | A |
| 74 | pmp001271 | 679.39 | 678.34 | C_33_H_58_O_14_ | [M+H]+ | 2,3-Dihydroxypropyl-9,12-octadecadienoate-hexose-hexose | Lipids | Free fatty acids | A |
| 75 | pmp001273 | 520.34 | 519.30 | C_26_H_50_NO_7_P | [M+H]+ | LysoPC(18:2) | Lipids | LPC | A |
| 76 | pmp001276 | 677.37 | 676.32 | C_33_H_56_O_14_ | [M+H]+ | 2,3-Dihydroxypropyl-9,12,15-octadecatrienoate-hexose-hexose | Lipids | Free fatty acids | A |
| 77 | pmp001278 | 496.34 | 495.30 | C_24_H_50_NO_7_P | [M+H]+ | LysoPC(16:0) | Lipids | LPC | A |
| 78 | pmp001281 | 522.36 | 521.31 | C_26_H_52_NO_7_P | [M+H]+ | LysoPC(18:1) | Lipids | LPC | A |
| 79 | pmp001286 | 524.37 | 523.33 | C_26_H_54_NO_7_P | [M+H]+ | LysoPC(18:0) | Lipids | LPC | A |
| 80 | Hmjp007381 | 515.31 | 514.30 | C_27_H_46_O_9_ | [M+H]+ | Glyceryl linoleate-O-Hexoside 1 | Lipids | Glycerol ester | B |
| 81 | Hmjp007544 | 515.31 | 514.30 | C_27_H_46_O_9_ | [M+H]+ | Glyceryl linoleate-O-Hexoside 2 | Lipids | Glycerol ester | B |
| 82 | Hmyn005706 | 653.38 | 654.38 | C_31_H_58_O_14_ | [M-H]- | DMGM(16:0) | Lipids | Glycerol ester | B |
| 83 | Hmyn007081 | 481.26 | 482.26 | C_22_H_43_O_9_P | [M-H]- | LysoPG(16:1) | Lipids | Glycerol ester | B |
| 84 | Hmyn007168 | 483.27 | 484.28 | C_22_H_45_O_9_P | [M-H]- | LysoPG(16:0) | Lipids | Glycerol ester | B |
| 85 | Qingke_Rfmb087-1-1 | 311.22 | 312.21 | C_18_H_32_O_4_ | [M-H]- | 13-Oxo-9-hydroxy-10-octadecenoic acid | Lipids | Free fatty acids | B |
| 86 | mws0120 | 258.11 | 257.10 | C_8_H_20_NO_6_P | [M+H]+ | Choline alfoscerate | Lipids | PC | B |
| 87 | mws0371 | 327.20 | 328.22 | C_22_H_32_O_2_ | [M-H]- | Cis-4,7,10,13,16,19-Docosahexaenoic Acid(C22:6n3) | Lipids | Free fatty acids | B |
| 88 | pmb0856 | 480.31 | 479.27 | C_23_H_46_NO_7_P | [M+H]+ | LysoPE 18:1(2n isomer) | Lipids | LPE | B |
| 89 | pmb0890 | 355.10 | 354.25 | C_21_H_38_O_4_ | [M+H]+ | MAG(18:2) | Lipids | Glycerol ester | B |
| 90 | pmb1656 | 353.10 | 352.24 | C_21_H_36_O_4_ | [M+H]+ | MAG(18:3)isomer4 | Lipids | Glycerol ester | B |
| 91 | pmb2444 | 353.20 | 352.24 | C_21_H_36_O_4_ | [M+H]+ | MAG(18:3)isomer1 | Lipids | Glycerol ester | B |
| 92 | pmn001610 | 307.30 | 308.25 | C_20_H_36_O_2_ | [M-H]- | Eicosadienoic acid | Lipids | Free fatty acids | B |
| 93 | pmp001283 | 355.29 | 354.25 | C_21_H_38_O_4_ | [M+H]+ | Glyceryl linoleate | Lipids | Glycerol ester | B |
| 94 | Lmtn006600 | 277.22 | 278.23 | C_18_H_30_O_2_ | [M-H]- | Trichosanic Acid | Organic acids | Organic acids | A |
| 95 | mws0159 | 163.05 | 164.04 | C_9_H_8_O_3_ | [M-H]- | Phenylpyruvic acid | Organic acids | Organic acids | A |
| 96 | mws0192 | 117.03 | 118.02 | C_4_H_6_O_4_ | [M-H]- | Succinic acid | Organic acids | Organic acids | A |
| 97 | mws0237 | 187.10 | 188.09 | C_9_H_16_O_4_ | [M-H]- | Anchoic Acid | Organic acids | Organic acids | A |
| 98 | mws0242 | 173.10 | 174.08 | C_8_H_14_O_4_ | [M-H]- | SubericAcid | Organic acids | Organic acids | A |
| 99 | mws0275 | 133.00 | 134.02 | C_4_H_6_O_5_ | [M-H]- | L-(-)-Malic acid | Organic acids | Organic acids | A |
| 100 | mws0281 | 191.00 | 192.02 | C_6_H_8_O_7_ | [M-H]- | Citric Acid | Organic acids | Organic acids | A |
| 101 | mws0344 | 165.05 | 166.04 | C_5_H_10_O_6_ | [M-H]- | D-Xylonic acid | Organic acids | Organic acids | A |
| 102 | mws0376 | 115.00 | 116.01 | C_4_H_4_O_4_ | [M-H]- | Fumaric acid | Organic acids | Organic acids | A |
| 103 | mws0425 | 129.00 | 130.02 | C_5_H_6_O_4_ | [M-H]- | Citraconic acid | Organic acids | Organic acids | A |
| 104 | mws0474 | 229.15 | 230.14 | C_12_H_22_O_4_ | [M-H]- | Dodecanedioic aicd | Organic acids | Organic acids | A |
| 105 | pme0266 | 201.12 | 202.11 | C_10_H_18_O_4_ | [M-H]- | Sebacic acid | Organic acids | Organic acids | A |
| 106 | mws0147 | 117.10 | 118.06 | C_5_H_10_O_3_ | [M-H]- | 3-Hydroxy-3-methyl butyric acid | Organic acids | Organic acids | B |
| 107 | mws0154 | 173.05 | 174.04 | C_7_H_10_O_5_ | [M-H]- | Shikimic acid | Organic acids | Organic acids | B |
| 108 | mws0177 | 111.02 | 112.01 | C_5_H_4_O_3_ | [M-H]- | 2-Furanoic acid | Organic acids | Organic acids | B |
| 109 | mws0206 | 103.05 | 104.04 | C_4_H_8_O_3_ | [M-H]- | (S)-2-Hydroxybutanoicacid | Organic acids | Organic acids | B |
| 110 | mws0208 | 145.06 | 146.05 | C_6_H_10_O_4_ | [M-H]- | Adipic Acid | Organic acids | Organic acids | B |
| 111 | mws0236 | 124.00 | 125.01 | C_2_H_7_NO_3_S | [M-H]- | 2-Aminoethanesulfonic acid | Organic acids | Organic acids | B |
| 112 | mws0277 | 191.00 | 192.05 | C_7_H_12_O_6_ | [M-H]- | Kinic acid | Organic acids | Organic acids | B |
| 113 | mws0341 | 131.08 | 132.07 | C_6_H_12_O_3_ | [M-H]- | (S)-(-)-2-Hydroxyisocaproic acid | Organic acids | Organic acids | B |
| 114 | mws0345 | 128.07 | 129.07 | C_6_H_11_NO_2_ | [M-H]- | Pipecolinic acid | Organic acids | Organic acids | B |
| 115 | mws0373 | 129.06 | 130.06 | C_6_H_10_O_3_ | [M-H]- | 4-Methyl-2-oxovalerate | Organic acids | Organic acids | B |
| 116 | mws0470 | 117.02 | 118.02 | C_4_H_6_O_4_ | [M-H]- | Methylmalonic acid | Organic acids | Organic acids | B |
| 117 | mws0473 | 131.04 | 132.04 | C_5_H_8_O_4_ | [M-H]- | 2-Methylsuccinic acid | Organic acids | Organic acids | B |
| 118 | mws0489 | 149.03 | 150.03 | C_8_H_6_O_3_ | [M-H]- | Benzoylformic acid | Organic acids | Organic acids | B |
| 119 | mws0567 | 146.09 | 145.08 | C_5_H_11_N_3_O_2_ | [M+H]+ | 4-Guanidinobutyric acid | Organic acids | Organic acids | B |
| 120 | mws0574 | 103.05 | 104.04 | C_4_H_8_O_3_ | [M-H]- | α-Hydroxyisobutyric acid | Organic acids | Organic acids | B |
| 121 | mws0576 | 103.05 | 104.04 | C_4_H_8_O_3_ | [M-H]- | 3-Hydroxybutyrate | Organic acids | Organic acids | B |
| 122 | mws0639 | 153.20 | 154.02 | C_7_H_6_O_4_ | [M-H]- | 2,3-Dihydroxybenzoic Acid | Organic acids | Organic acids | B |
| 123 | mws0823 | 115.00 | 116.04 | C_5_H_8_O_3_ | [M-H]- | 3-Methyl-2-Oxobutanoic acid | Organic acids | Organic acids | B |
| 124 | mws0851 | 143.11 | 166.09 | C_8_H_15_NaO_2_ | [M-Na]- | Sodium Valproate | Organic acids | Organic acids | B |
| 125 | mws0924 | 145.06 | 146.05 | C_6_H_10_O_4_ | [M-H]- | 2-Methylglutaric acid | Organic acids | Organic acids | B |
| 126 | mws0972 | 131.08 | 132.07 | C_6_H_12_O_3_ | [M-H]- | 5-Hydroxyhexanoic acid | Organic acids | Organic acids | B |
| 127 | mws1189 | 193.10 | 194.03 | C_6_H_10_O_7_ | [M-H]- | D-Galacturonic acid(Gal A) | Organic acids | Organic acids | B |
| 128 | mws1213 | 193.10 | 194.03 | C_6_H_10_O_7_ | [M-H]- | Aldehydo-D-galacturonate | Organic acids | Organic acids | B |
| 129 | pmb3099 | 153.00 | 154.03 | C_4_H_11_O_4_P | [M-H]- | Diethyl phosphate | Organic acids | Organic acids | B |
| 130 | pme0274 | 132.09 | 131.09 | C_6_H_13_NO_2_ | [M+H]+ | 6-Aminocaproic acid | Organic acids | Organic acids | B |
| 131 | pme0282 | 165.03 | 166.02 | C_8_H_6_O_4_ | [M-H]- | Phthalic acid | Organic acids | Organic acids | B |
| 132 | pme0295 | 146.07 | 145.07 | C_6_H_11_NO_3_ | [M+H]+ | 4-Acetamidobutyric acid | Organic acids | Organic acids | B |
| 133 | pme2049 | 103.00 | 104.04 | C_4_H_8_O_3_ | [M-H]- | 2-Hydroxybutanoic acid | Organic acids | Organic acids | B |
| 134 | pme3009 | 175.00 | 174.01 | C_6_H_6_O_6_ | [M+H]+ | Trans-Citridic acid | Organic acids | Organic acids | B |
| 135 | pme3011 | 104.06 | 103.06 | C_4_H_9_NO_2_ | [M+H]+ | γ-Aminobutyric acid | Organic acids | Organic acids | B |
| 136 | pme3154 | 147.07 | 148.06 | C_6_H_12_O_4_ | [M-H]- | (Rs)-Mevalonic acid | Organic acids | Organic acids | B |
| 137 | pme3207 | 141.03 | 142.02 | C_6_H_6_O_4_ | [M-H]- | Trans,trans-Muconic acid | Organic acids | Organic acids | B |
| 138 | mws0102 | 160.05 | 161.04 | C_9_H_7_NO_2_ | [M-H]- | Indole-5-carboxylic acid | Alkaloids | Plumerane | A |
| 139 | mws0103 | 146.10 | 145.05 | C_9_H_7_NO | [M+H]+ | Indole-3-carboxaldehyde | Alkaloids | Plumerane | A |
| 140 | mws1383 | 243.10 | 242.07 | C_12_H_10_N_4_O_2_ | [M+H]+ | Lumichrome | Alkaloids | Alkaloids | A |
| 141 | mws1417 | 160.00 | 161.04 | C_9_H_7_NO_2_ | [M-H]- | Indole-3-carboxylic acid | Alkaloids | Plumerane | A |
| 142 | pmb1912 | 474.20 | 473.14 | C_20_H_23_N_7_O_7_ | [M+H]+ | 10-Formyl-THF | Alkaloids | Alkaloids | A |
| 143 | pmp001198 | 132.10 | 131.09 | C_6_H_13_NO_2_ | [M+H]+ | 6-Deoxyfagomine | Alkaloids | Alkaloids | A |
| 144 | pmp001272 | 478.29 | 477.25 | C_23_H_44_NO_7_P | [M+H]+ | 3-{(2-Aminoethoxy)(hydroxy)phosphoryl]oxy}-2-12-octadecadienoate | Alkaloids | Alkaloids | A |
| 145 | pmp001274 | 542.32 | 541.28 | C_28_H_48_NO_7_P | [M+H]+ | 2-Hydroxy-5,8,11,14,17-icosapentaenoyloxy ]propyl-2-(trimethylammonio)ethyl phosphate | Alkaloids | Alkaloids | A |
| 146 | pmp001275 | 672.42 | 671.36 | C_31_H_61_O_14_N | [M+H]+ | 3-Hydroxypropyl palmitate glc-glucosamine | Alkaloids | Alkaloids | A |
| 147 | pmp001277 | 454.30 | 453.25 | C_21_H_44_NO_7_P | [M+H]+ | 3-{(2-Aminoethoxy)(hydroxy)phosphoryl]oxy}-2-hydroxypropyl palmitate | Alkaloids | Alkaloids | A |
| 148 | pmp001287 | 120.08 | 119.07 | C_8_H_9_N | [M+H]+ | N-Benzylmethylene isomethylamine | Alkaloids | Alkaloids | A |
| 149 | mws0005 | 161.10 | 160.09 | C_10_H_12_N_2_ | [M+H]+ | Tryptamine | Alkaloids | Plumerane | B |
| 150 | mws0017 | 146.20 | 145.14 | C_7_H_19_N_3_ | [M+H]+ | Spermidine | Alkaloids | Phenolamine | B |
| 151 | mws0018 | 203.00 | 202.20 | C_10_H_26_N_4_ | [M+H]+ | Spermine | Alkaloids | Phenolamine | B |
| 152 | mws0146 | 138.05 | 137.04 | C_7_H_7_NO_2_ | [M+H]+ | Nicotinic Acid Methyl Ester(Methyl Nicotinate) | Alkaloids | Alkaloids | B |
| 153 | mws0677 | 219.00 | 218.10 | C_12_H_14_N_2_O_2_ | [M+H]+ | N-Acetyl-5-hydroxytryptamine | Alkaloids | Alkaloids | B |
| 154 | mws1320 | 160.08 | 161.08 | C_10_H_11_NO | [M-H]- | Tryptophol | Alkaloids | Plumerane | B |
| 155 | mws1346 | 162.07 | 161.06 | C_6_H_11_NO_4_ | [M+H]+ | DL-2-Aminoadipic acid | Alkaloids | Alkaloids | B |
| 156 | mws1640 | 205.13 | 204.12 | C_12_H_16_N_2_O | [M+H]+ | N-Methylcytisine | Alkaloids | Alkaloids | B |
| 157 | pmb0484 | 104.10 | 103.09 | C_5_H_13_NO | [M+H]+ | Choline | Alkaloids | Alkaloids | B |
| 158 | pmb0492 | 584.20 | 583.27 | C_34_H_37_N_3_O_6_ | [M+H]+ | N',N'',N'''-p-Coumaroyl-cinnamoyl-caffeoyl spermidine | Alkaloids | Phenolamine | B |
| 159 | pmb0501 | 131.10 | 130.11 | C_5_H_14_N_4_ | [M+H]+ | Agmatine | Alkaloids | Phenolamine | B |
| 160 | pme1137 | 140.00 | 139.02 | C_6_H_5_NO_3_ | [M+H]+ | 6-Hydroxynicotinic acid | Alkaloids | Alkaloids | B |
| 161 | pme1691 | 147.12 | 146.11 | C_7_H_16_NO_2_ | [M+H]+ | Acetylcholine | Alkaloids | Alkaloids | B |
| 162 | pme2693 | 131.00 | 130.10 | C_6_H_14_N_2_O | [M+H]+ | N-Acetylputrescine | Alkaloids | Alkaloids | B |
| 163 | pmp000479 | 252.20 | 251.21 | C_16_H_29_NO | [M+H]+ | N-isobutyl-2E,4E-dodedienamide | Alkaloids | Alkaloids | B |
| 164 | pmp000628 | 261.16 | 260.14 | C_15_H_20_N_2_O_2_ | [M+H]+ | 9α-Hydroxysophoramine | Alkaloids | Alkaloids | B |
| 165 | pmp001248 | 428.19 | 428.17 | C_20_H_30_NO_9_+ | [M]+ | Caffeoylcholine 5-glucoside | Alkaloids | Alkaloids | B |
| 166 | pmp001249 | 428.19 | 428.17 | C_20_H_30_NO_9_+ | [M]+ | Caffeoylcholine 6-glucoside | Alkaloids | Alkaloids | B |
| 167 | YC512118 | 282.28 | 281.25 | C_18_H_35_NO | [M+H]+ | Octadecenoic amide | Others | Others | A |
| 168 | mws0133 | 123.10 | 122.04 | C_6_H_6_N_2_O | [M+H]+ | Nicotinamide | Others | Vitamin | A |
| 169 | mws0198 | 179.00 | 180.05 | C_6_H_12_O_6_ | [M-H]- | D-(+)-Glucose | Others | Saccharides and Alcohols | A |
| 170 | mws0232 | 377.00 | 376.12 | C_17_H_20_N_4_O_6_ | [M+H]+ | Riboflavin | Others | Vitamin | A |
| 171 | mws0264 | 341.00 | 342.10 | C_12_H_22_O_11_ | [M-H]- | D-(+)-TrehaloseAnhydrous | Others | Saccharides and Alcohols | A |
| 172 | mws0866 | 259.00 | 260.02 | C_6_H_13_O_9_P | [M-H]- | D-Glucose 6-phosphate | Others | Saccharides and Alcohols | A |
| 173 | mws1038 | 277.13 | 278.11 | C_11_H_22_N_2_O_4_S | [M-H]- | (R)-Pantetheine | Others | Others | A |
| 174 | mws1080 | 341.10 | 342.10 | C_12_H_22_O_11_ | [M-H]- | Galactinol | Others | Saccharides and Alcohols | A |
| 175 | mws1090 | 259.00 | 260.02 | C_6_H_13_O_9_P | [M-H]- | Glucose-1-phosphate | Others | Saccharides and Alcohols | A |
| 176 | mws1333 | 341.12 | 342.10 | C_12_H_22_O_11_ | [M-H]- | Melibiose | Others | Saccharides and Alcohols | A |
| 177 | mws1337 | 220.20 | 219.10 | C_9_H_17_NO_5_ | [M+H]+ | D-Pantothenic Acid | Others | Vitamin | A |
| 178 | mws4170 | 179.06 | 180.05 | C_6_H_12_O_6_ | [M-H]- | D-Glucose | Others | Saccharides and Alcohols | A |
| 179 | mws5038 | 341.11 | 342.10 | C_12_H_22_O_11_ | [M-H]- | Isomaltulose | Others | Saccharides and Alcohols | A |
| 180 | pme0490 | 124.00 | 123.03 | C_6_H_5_NO_2_ | [M+H]+ | Nicotinic acid | Others | Vitamin | A |
| 181 | pme0519 | 341.00 | 342.10 | C_12_H_22_O_11_ | [M-H]- | D-(+)-Sucrose | Others | Saccharides and Alcohols | A |
| 182 | pme0534 | 195.16 | 196.05 | C_6_H_12_O_7_ | [M-H]- | Gluconic acid | Others | Saccharides and Alcohols | A |
| 183 | pme1383 | 170.10 | 169.07 | C_8_H_11_NO_3_ | [M+H]+ | Pyridoxine | Others | Vitamin | A |
| 184 | pmn001423 | 385.18 | 386.17 | C_19_H_30_O_8_ | [M-H]- | Roseoside | Others | Others | A |
| 185 | Hmfn00531 | 175.03 | 176.03 | C_6_H_8_O_6_ | [M-H]- | L-Ascorbic acid | Others | Vitamin | B |
| 186 | mws0213 | 151.00 | 152.06 | C_5_H_12_O_5_ | [M-H]- | Ribitol | Others | Saccharides and Alcohols | B |
| 187 | mws0214 | 181.00 | 182.07 | C_6_H_14_O_6_ | [M-H]- | D-Sorbitol | Others | Saccharides and Alcohols | B |
| 188 | mws0437 | 151.15 | 152.06 | C_5_H_12_O_5_ | [M-H]- | D-Arabitol | Others | Saccharides and Alcohols | B |
| 189 | mws0491 | 122.10 | 121.08 | C_8_H_11_N | [M+H]+ | Phenethylamine | Others | Others | B |
| 190 | mws0601 | 110.10 | 111.03 | C_5_H_5_NO_2_ | [M-H]- | Pyrrole-2-carboxylic acid | Others | Others | B |
| 191 | mws0889 | 135.00 | 136.03 | C_4_H_8_O_5_ | [M-H]- | Threonate | Others | Saccharides and Alcohols | B |
| 192 | mws1155 | 181.20 | 182.07 | C_6_H_14_O_6_ | [M-H]- | Mannitol | Others | Saccharides and Alcohols | B |
| 193 | mws1429 | 223.10 | 224.13 | C_13_H_20_O_3_ | [M-H]- | Vomifoliol | Others | Others | B |
| 194 | mws1562 | 361.12 | 362.10 | C_15_H_22_O_10_ | [M-H]- | Catalpol | Others | Others | B |
| 195 | mws1589 | 503.20 | 504.14 | C_18_H_32_O_16_ | [M-H]- | Panose | Others | Saccharides and Alcohols | B |
| 196 | mws1593 | 665.10 | 666.18 | C_24_H_42_O_21_ | [M-H]- | Maltotetraose | Others | Saccharides and Alcohols | B |
| 197 | mws2104 | 193.07 | 194.07 | C_7_H_14_O_6_ | [M-H]- | D-Pinitol | Others | Others | B |
| 198 | mws2523 | 421.10 | 422.06 | C_12_H_23_O_14_P | [M-H]- | Trehalose 6-phosphate | Others | Saccharides and Alcohols | B |
| 199 | mws2608 | 222.10 | 221.08 | C_8_H_15_NO_6_ | [M+H]+ | N-Acetyl-D-galactosamine | Others | Others | B |
| 200 | mws4163 | 665.20 | 666.18 | C_24_H_42_O_21_ | [M-H]- | 1,1-Kestotetraose | Others | Saccharides and Alcohols | B |
| 201 | mws4174 | 222.09 | 221.08 | C_8_H_15_NO_6_ | [M+H]+ | N-Acetyl-β-D-mannosamine | Others | Others | B |
| 202 | mws4175 | 175.03 | 176.02 | C_6_H_8_O_6_ | [M-H]- | D-Glucurono-6,3-lactone | Others | Others | B |
| 203 | mws5040 | 365.11 | 364.08 | C_12_H_21_O_11_Na | [M+H]+ | Turanose | Others | Saccharides and Alcohols | B |
| 204 | pmb3079 | 300.10 | 301.04 | C_8_H_16_NO_9_P | [M-H]- | N-Acetyl-D-glucosamine 1-phosphate | Others | Others | B |
| 205 | pmb3081 | 289.10 | 289.99 | C_6_H_11_PO_11_ | [M-H]- | Glucarate O-Phosphoric acid | Others | Saccharides and Alcohols | B |
| 206 | pme0500 | 503.44 | 504.14 | C_18_H_32_O_16_ | [M-H]- | D-(+)-Melezitose | Others | Saccharides and Alcohols | B |
| 207 | pme0513 | 151.00 | 152.06 | C_5_H_12_O_5_ | [M-H]- | Xylitol | Others | Saccharides and Alcohols | B |
| 208 | pme0516 | 179.16 | 180.05 | C_6_H_12_O_6_ | [M-H]- | Inositol | Others | Saccharides and Alcohols | B |
| 209 | pme2019 | 149.00 | 150.04 | C_5_H_10_O_5_ | [M-H]- | DL-Arabinose | Others | Saccharides and Alcohols | B |
| 210 | pme2237 | 181.08 | 182.07 | C_6_H_14_O_6_ | [M-H]- | Dulcitol | Others | Saccharides and Alcohols | B |
| 211 | pme2253 | 177.14 | 178.04 | C_6_H_10_O_6_ | [M-H]- | L-Gulonic-γ-lactone | Others | Saccharides and Alcohols | B |
| 212 | pme2266 | 245.09 | 244.08 | C_10_H_16_N_2_O_3_S | [M+H]+ | Biotin | Others | Vitamin | B |
| 213 | pme2433 | 106.00 | 105.07 | C_4_H_11_NO_2_ | [M+H]+ | Diethanolamine | Others | Others | B |
| 214 | pme2596 | 184.05 | 183.05 | C_8_H_9_NO_4_ | [M+H]+ | 4-Pyridoxic acid | Others | Vitamin | B |
| 215 | pme3081 | 169.09 | 168.08 | C_8_H_12_N_2_O_2_ | [M+H]+ | 4-(Aminomethyl)-5-(hydroxymethyl)-2-methylpyridin-3-ol | Others | Others | B |
| 216 | pme3705 | 193.14 | 194.03 | C_6_H_10_O_7_ | [M-H]- | D-Glucoronic acid | Others | Saccharides and Alcohols | B |
| 217 | pmp001045 | 331.17 | 330.11 | C_15_H_22_O_8_ | [M+H]+ | Bartsioside | Others | Others | B |
| 218 | pmp001269 | 286.31 | 285.30 | C_18_H_39_NO | [M+H]+ | Hexadecyl ethanolamine | Others | Others | B |
| 219 | pmp001282 | 544.34 | 543.30 | C_28_H_50_NO_7_P | [M+H]+ | Propyl2-(trimethylammonio)ethyl phosphate | Others | Others | B |
| 220 | pmn001492 | 187.10 | 188.04 | C_11_H_8_O_3_ | [M-H]- | Ayapin | Lignans and Coumarins | Coumarins | A |
| 221 | Qingke_Rfmb262-der01-3 | 579.21 | 580.19 | C_28_H_36_O_13_ | [M-H]- | Syringaresinol-Hex | Lignans and Coumarins | Lignans | B |
| 222 | Qingke_Rfmb262-der14-1 | 621.21 | 622.20 | C_30_H_38_O_14_ | [M-H]- | Syringaresinol-aceGlu | Lignans and Coumarins | Lignans | B |
| 223 | mws0097 | 357.14 | 358.13 | C_20_H_22_O_6_ | [M-H]- | Pinoresinol | Lignans and Coumarins | Lignans | B |
| 224 | mws1014 | 209.20 | 208.03 | C_10_H_8_O_5_ | [M+H]+ | Fraxetin | Lignans and Coumarins | Coumarins | B |
| 225 | mws1015 | 339.10 | 340.07 | C_15_H_16_O_9_ | [M-H]- | Esculin(6,7-DihydroxyCoumarin-6-glucoside) | Lignans and Coumarins | Coumarins | B |
| 226 | mws1727 | 355.15 | 354.13 | C_21_H_22_O_5_ | [M+H]+ | Notopterol | Lignans and Coumarins | Coumarins | B |
| 227 | mws2625 | 359.15 | 358.13 | C_20_H_22_O_6_ | [M+H]+ | Matairesinol | Lignans and Coumarins | Lignans | B |
| 228 | mws5039 | 341.09 | 340.07 | C_15_H_16_O_9_ | [M+H]+ | Esculin Hydrate | Lignans and Coumarins | Coumarins | B |
| 229 | pme2987 | 147.10 | 148.05 | C_9_H_8_O_2_ | [M-H]- | 3,4-Dihydrocoumarin | Lignans and Coumarins | Coumarins | B |
| 230 | pme2993 | 193.10 | 192.04 | C_10_H_8_O_4_ | [M+H]+ | Scopoletin(7-Hydroxy-5-methoxycoumarin) | Lignans and Coumarins | Coumarins | B |
| 231 | pme2996 | 161.00 | 162.03 | C_9_H_6_O_3_ | [M-H]- | 4-Hydroxycoumarin | Lignans and Coumarins | Coumarins | B |
| 232 | pmn001368 | 699.25 | 700.22 | C_32_H_44_O_17_ | [M-H]- | Olivin Diglucoside | Lignans and Coumarins | Lignans | B |
| 233 | pmn001370 | 681.24 | 682.21 | C_32_H_42_O_16_ | [M-H]- | Pinoresinol diglucoside | Lignans and Coumarins | Lignans | B |
| 234 | pmn001502 | 387.14 | 388.13 | C_21_H_24_O_7_ | [M-H]- | Medioresinol | Lignans and Coumarins | Lignans | B |
| 235 | mws0183 | 153.10 | 154.02 | C_7_H_6_O_4_ | [M-H]- | Protocatechuic acid | Flavonoids | Flavanols | A |
| 236 | pme2459 | 449.10 | 448.08 | C_21_H_20_O_11_ | [M+H]+ | Luteolin 7-O-glucoside(Cynaroside) | Flavonoids | Flavonoid | A |
| 237 | pme2954 | 303.04 | 302.03 | C_15_H_10_O_7_ | [M+H]+ | Quercetin | Flavonoids | Flavonols | A |
| 238 | pmn001713 | 593.17 | 594.13 | C_27_H_30_O_15_ | [M-H]- | Luteolin-7-O-β-D-rutinoside | Flavonoids | Flavonoid | A |
| 239 | pmp000113 | 389.10 | 388.10 | C_20_H_20_O_8_ | [M+H]+ | 5-Hydroxy-6,7,8,3',4'-pentamethoxyflavone | Flavonoids | Flavonoid | A |
| 240 | GQ512002 | 623.10 | 624.14 | C_28_H_32_O_16_ | [M-H]- | Isorhamnetin-3-O-rutinoside | Flavonoids | Flavonols | B |
| 241 | mws0024 | 169.02 | 170.02 | C_7_H_6_O_5_ | [M-H]- | Gallic acid | Flavonoids | Flavanols | B |
| 242 | mws0072 | 433.11 | 432.09 | C_21_H_20_O_10_ | [M+H]+ | Apigenin 5-O-glucoside | Flavonoids | Flavonoid | B |
| 243 | mws1073 | 595.20 | 594.13 | C_27_H_30_O_15_ | [M+H]+ | Apigenin 6,8-C-diglucoside | Flavonoids | Flavonoid | B |
| 244 | pmb0623 | 625.20 | 624.14 | C_28_H_32_O_16_ | [M+H]+ | 6-C-Hexosyl chrysoeriol O-hexoside | Flavonoids | Flavonoid carbonoside | B |
| 245 | pmb3041 | 521.10 | 522.08 | C_23_H_22_O_14_ | [M-H]- | Tricin O-saccharic acid | Flavonoids | Flavonoid | B |
| 246 | pmb3894 | 329.10 | 330.06 | C_17_H_14_O_7_ | [M-H]- | Di-O-methylquercetin | Flavonoids | Flavonols | B |
| 247 | pme0444 | 493.13 | 493.14 | C_23_H_25_O_12_+ | [M]+ | Malvidin 3-O-glucoside (Oenin) | Flavonoids | Anthocyanins | B |
| 248 | pme2482 | 139.10 | 138.03 | C_7_H_6_O_3_ | [M+H]+ | Protocatechuic aldehyde | Flavonoids | Flavanols | B |
| 249 | pmn001668 | 415.10 | 416.13 | C_22_H_24_O_8_ | [M-H]- | Apigenin-3-O-α-L-rhamnoside | Flavonoids | Flavonoid | B |
| 250 | pmp000127 | 625.20 | 624.14 | C_28_H_32_O_16_ | [M+H]+ | Chysoeriol-6,8-di-C-glucoside | Flavonoids | Flavonoid carbonoside | B |
| 251 | pmp001106 | 595.17 | 594.13 | C_27_H_30_O_15_ | [M+H]+ | Vitexin-2-O-D-glucopyranoside | Flavonoids | Flavonoid carbonoside | B |
| 252 | mws0248 | 243.10 | 244.06 | C_9_H_12_N_2_O_6_ | [M-H]- | Uridine | Nucleotides and derivatives | Nucleotides and derivatives | A |
| 253 | mws0255 | 112.00 | 111.04 | C_4_H_5_N_3_O | [M+H]+ | Cytosine | Nucleotides and derivatives | Nucleotides and derivatives | A |
| 254 | mws0668 | 283.00 | 284.06 | C_10_H_12_N_4_O_6_ | [M-H]- | Xanthosine | Nucleotides and derivatives | Nucleotides and derivatives | A |
| 255 | mws0847 | 150.00 | 149.06 | C_6_H_7_N_5_ | [M+H]+ | 1-Methyladenine | Nucleotides and derivatives | Nucleotides and derivatives | A |
| 256 | mws1060 | 267.08 | 268.07 | C_10_H_12_N_4_O_5_ | [M-H]- | 9-(β-D-Arabinofuranosyl)hypoxanthine | Nucleotides and derivatives | Nucleotides and derivatives | A |
| 257 | pmb2922 | 565.10 | 566.03 | C_15_H_24_N_2_O_17_P_2_ | [M-H]- | Uridine 5'-diphospho-D-glucose | Nucleotides and derivatives | Nucleotides and derivatives | A |
| 258 | pme0040 | 136.10 | 135.05 | C_5_H_5_N_5_ | [M+H]+ | Adenine | Nucleotides and derivatives | Nucleotides and derivatives | A |
| 259 | pme0183 | 152.10 | 151.04 | C_5_H_5_N_5_O | [M+H]+ | 2-Hydroxy-6-aminopurine | Nucleotides and derivatives | Nucleotides and derivatives | A |
| 260 | pme0230 | 268.10 | 267.08 | C_10_H_13_N_5_O_4_ | [M+H]+ | Adenosine | Nucleotides and derivatives | Nucleotides and derivatives | A |
| 261 | pme0257 | 111.03 | 112.02 | C_4_H_4_N_2_O_2_ | [M-H]- | Uracil | Nucleotides and derivatives | Nucleotides and derivatives | A |
| 262 | pme1109 | 152.00 | 151.04 | C_5_H_5_N_5_O | [M+H]+ | Guanine | Nucleotides and derivatives | Nucleotides and derivatives | A |
| 263 | pme1178 | 284.09 | 283.08 | C_10_H_13_N_5_O_5_ | [M+H]+ | Guanosine | Nucleotides and derivatives | Nucleotides and derivatives | A |
| 264 | pme1474 | 298.00 | 297.08 | C_11_H_15_N_5_O_3_S | [M+H]+ | 5'-Deoxy-5'-(methylthio)adenosine | Nucleotides and derivatives | Nucleotides and derivatives | A |
| 265 | pme3337 | 384.00 | 383.09 | C_14_H_17_N_5_O_8_ | [M+H]+ | N6-Succinyl Adenosine | Nucleotides and derivatives | Nucleotides and derivatives | A |
| 266 | pme3967 | 312.00 | 311.11 | C_12_H_17_N_5_O_5_ | [M+H]+ | 2-(Dimethylamino)guanosine | Nucleotides and derivatives | Nucleotides and derivatives | A |
| 267 | mws0572 | 126.10 | 125.05 | C_5_H_7_N_3_O | [M+H]+ | 5-Methylcytosine | Nucleotides and derivatives | Nucleotides and derivatives | B |
| 268 | mws0609 | 344.20 | 345.03 | C_10_H_12_N_5_O_7_P | [M-H]- | Guanosine 3',5'-cyclic monophosphate | Nucleotides and derivatives | Nucleotides and derivatives | B |
| 269 | mws0724 | 298.10 | 299.07 | C_10_H_13_N_5_O_6_ | [M-H]- | 8-Hydroxyguanosine | Nucleotides and derivatives | Nucleotides and derivatives | B |
| 270 | mws0863 | 213.00 | 234.99 | C_5_H_9_NaO_7_P | [M-Na]- | 2-Deoxyribose 5-phosphate | Nucleotides and derivatives | Nucleotides and derivatives | B |
| 271 | mws0984 | 236.10 | 237.08 | C_9_H_11_N_5_O_3_ | [M-H]- | Sepiapterin(L-SEPIAPTERIN)COFACTOR OF NITRIC OX | Nucleotides and derivatives | Nucleotides and derivatives | B |
| 272 | pmb0530 | 664.20 | 663.08 | C_21_H_27_N_7_O_14_P_2_ | [M+H]+ | Nicotinic acid adenine dinucleotide | Nucleotides and derivatives | Nucleotides and derivatives | B |
| 273 | pmb0981 | 348.20 | 347.05 | C_10_H_14_N_5_O_7_P | [M+H]+ | Adenosine 5'-monophosphate | Nucleotides and derivatives | Nucleotides and derivatives | B |
| 274 | pmb0998 | 364.10 | 363.04 | C_10_H_14_N_5_O_8_P | [M+H]+ | Guanosine 5'-monophosphate | Nucleotides and derivatives | Nucleotides and derivatives | B |
| 275 | pme0033 | 137.00 | 136.03 | C_5_H_4_N_4_O | [M+H]+ | Hypoxanthine | Nucleotides and derivatives | Nucleotides and derivatives | B |
| 276 | pme0256 | 151.03 | 152.03 | C_5_H_4_N_4_O_2_ | [M-H]- | Xanthine | Nucleotides and derivatives | Nucleotides and derivatives | B |
| 277 | pme0264 | 243.09 | 242.08 | C_10_H_14_N_2_O_5_ | [M+H]+ | Thymidine | Nucleotides and derivatives | Nucleotides and derivatives | B |
| 278 | pme1173 | 137.00 | 136.03 | C_5_H_4_N_4_O | [M+H]+ | Allopurinol | Nucleotides and derivatives | Nucleotides and derivatives | B |
| 279 | pme1184 | 268.10 | 267.08 | C_10_H_13_N_5_O_4_ | [M+H]+ | Deoxyguanosine | Nucleotides and derivatives | Nucleotides and derivatives | B |
| 280 | pme1187 | 257.08 | 258.07 | C_10_H_14_N_2_O_6_ | [M-H]- | 5-Methyluridine | Nucleotides and derivatives | Nucleotides and derivatives | B |
| 281 | pme1194 | 228.00 | 227.08 | C_9_H_13_N_3_O_4_ | [M+H]+ | Deoxycytidine | Nucleotides and derivatives | Nucleotides and derivatives | B |
| 282 | pme1373 | 306.00 | 307.04 | C_9_H_14_N_3_O_7_P | [M-H]- | 2'-Deoxycytidine-5'-monophosphate | Nucleotides and derivatives | Nucleotides and derivatives | B |
| 283 | pme2801 | 167.00 | 166.04 | C_6_H_6_N_4_O_2_ | [M+H]+ | 7-Methylxanthine | Nucleotides and derivatives | Nucleotides and derivatives | B |
| 284 | pme3007 | 403.00 | 403.99 | C_9_H_14_N_2_O_12_P_2_ | [M-H]- | Uridine 5’-diphosphate | Nucleotides and derivatives | Nucleotides and derivatives | B |
| 285 | pme3174 | 324.00 | 323.04 | C_9_H_14_N_3_O_8_P | [M+H]+ | Cytidine 5'-monophosphate(Cytidylic acid) | Nucleotides and derivatives | Nucleotides and derivatives | B |
| 286 | pme3184 | 332.00 | 331.05 | C_10_H_14_N_5_O_6_P | [M+H]+ | 2'-Deoxyadenosine-5'-monophosphate | Nucleotides and derivatives | Nucleotides and derivatives | B |
| 287 | pme3188 | 323.00 | 324.02 | C_9_H_13_N_2_O_9_P | [M-H]- | Uridine 5'-monophosphate | Nucleotides and derivatives | Nucleotides and derivatives | B |
| 288 | pme3732 | 244.09 | 243.07 | C_9_H_13_N_3_O_5_ | [M+H]+ | Cytidine | Nucleotides and derivatives | Nucleotides and derivatives | B |
| 289 | pme3968 | 166.00 | 165.06 | C_6_H_7_N_5_O | [M+H]+ | 7-Methylguanine | Nucleotides and derivatives | Nucleotides and derivatives | B |
| 290 | Lmgn001670 | 137.03 | 138.03 | C_7_H_6_O_3_ | [M-H]- | Salicylic acid | Phenolic acids | Phenolic acids | A |
| 291 | Zmhn001926 | 299.08 | 300.08 | C_13_H_16_O_8_ | [M-H]- | Salicylic acid O-glycoside | Phenolic acids | Phenolic acids | A |
| 292 | Zmhn002301 | 325.09 | 326.10 | C_15_H_18_O_8_ | [M-H]- | p-Coumaric acid-O-glycoside | Phenolic acids | Phenolic acids | A |
| 293 | Zmxn001997 | 299.08 | 300.08 | C_13_H_16_O_8_ | [M-H]- | isosalicylic acid O-glycoside | Phenolic acids | Phenolic acids | A |
| 294 | mws0014 | 193.06 | 194.05 | C_10_H_10_O_4_ | [M-H]- | Ferulic acid | Phenolic acids | Phenolic acids | A |
| 295 | mws0027 | 197.05 | 198.04 | C_9_H_10_O_5_ | [M-H]- | Syringic acid | Phenolic acids | Phenolic acids | A |
| 296 | mws0028 | 167.00 | 168.04 | C_8_H_8_O_4_ | [M-H]- | Vanillic acid | Phenolic acids | Phenolic acids | A |
| 297 | mws0178 | 353.10 | 354.08 | C_16_H_18_O_9_ | [M-H]- | Chlorogenic acid | Phenolic acids | Phenolic acids | A |
| 298 | mws0180 | 153.00 | 154.02 | C_7_H_6_O_4_ | [M-H]- | 2,5-Dihydroxybenzoic acid | Phenolic acids | Phenolic acids | A |
| 299 | mws0458 | 151.05 | 152.04 | C_8_H_8_O_3_ | [M-H]- | Vanillin | Phenolic acids | Phenolic acids | A |
| 300 | mws0467 | 165.06 | 166.06 | C_9_H_10_O_3_ | [M-H]- | 3-(4-Hydroxyphenyl)-propionic acid | Phenolic acids | Phenolic acids | A |
| 301 | mws0628 | 121.04 | 122.03 | C_7_H_6_O_2_ | [M-H]- | 4-Hydroxybenzaldehyde | Phenolic acids | Phenolic acids | A |
| 302 | mws0749 | 123.03 | 124.05 | C7H8O2 | [M-H]- | 4-Hydroxybenzyl alcohol | Phenolic acids | Phenolic acids | A |
| 303 | mws0921 | 149.00 | 150.06 | C_9_H_10_O_2_ | [M-H]- | p-Coumaryl alcohol | Phenolic acids | Phenolic acids | A |
| 304 | mws1354 | 193.06 | 194.05 | C_10_H_10_O_4_ | [M-H]- | Trans-ferulic acid | Phenolic acids | Phenolic acids | A |
| 305 | mws2213 | 147.05 | 148.05 | C_9_H_8_O_2_ | [M-H]- | Cinnamic acid | Phenolic acids | Phenolic acids | A |
| 306 | pmb2871 | 315.10 | 316.07 | C_13_H_16_O_9_ | [M-H]- | 2,5-Dihydroxy benzoic acid O-hexside | Phenolic acids | Phenolic acids | A |
| 307 | pme1439 | 165.05 | 164.04 | C_9_H_8_O_3_ | [M+H]+ | p-Coumaric acid | Phenolic acids | Phenolic acids | A |
| 308 | pmn001367 | 315.07 | 316.07 | C_13_H_16_O_9_ | [M-H]- | Protocatechuic acid-4-glucoside | Phenolic acids | Phenolic acids | A |
| 309 | pmn001419 | 325.09 | 326.09 | C_15_H_18_O_8_ | [M-H]- | 1-O-[(E)-p-Cumaroyl]-β-D-glucopyranose | Phenolic acids | Phenolic acids | A |
| 310 | pmn001578 | 255.23 | 256.22 | C_16_H_32_O_2_ | [M-H]- | Hexadecanoic acid | Phenolic acids | Phenolic acids | A |
| 311 | pmn001710 | 521.13 | 522.11 | C_24_H_26_O_13_ | [M-H]- | Rosmarinyl Glucoside | Phenolic acids | Phenolic acids | A |
| 312 | pmp000545 | 355.10 | 354.08 | C_16_H_18_O_9_ | [M+H]+ | 4-Caffeoylquinic acid | Phenolic acids | Phenolic acids | A |
| 313 | pmp001113 | 153.05 | 152.04 | C_8_H_8_O_3_ | [M+H]+ | Dihydro-p-coumarat | Phenolic acids | Phenolic acids | A |
| 314 | pmp001285 | 149.02 | 148.01 | C_8_H_4_O_3_ | [M+H]+ | Phthalic anhydride | Phenolic acids | Phenolic acids | A |
| 315 | Huasheng_mad1395 | 311.08 | 312.04 | C_13_H_12_O_9_ | [M-H]- | Caffeoyl-pen | Phenolic acids | Phenolic acids | B |
| 316 | Huasheng_mad1424 | 427.06 | 426.03 | C_17_H_14_O_13_ | [M+H]+ | Ditartaroyl-hydroxylcoumarin | Phenolic acids | Phenolic acids | B |
| 317 | Li512113 | 451.10 | 452.08 | C_20_H_20_O_12_ | [M-H]- | Maleoyl-caffeoylquinic acid | Phenolic acids | Phenolic acids | B |
| 318 | Lmhn002926 | 279.05 | 280.06 | C_13_H_12_O_7_ | [M-H]- | p-coumaroylmalic acid | Phenolic acids | Phenolic acids | B |
| 319 | Lmhn003373 | 253.04 | 254.04 | C_11_H_10_O_7_ | [M-H]- | p-Hydroxybenzoylmalic acid | Phenolic acids | Phenolic acids | B |
| 320 | Lmlp002133 | 165.05 | 164.05 | C_9_H_8_O_3_ | [M+H]+ | Trans-p-Hydroxycinnamic acid | Phenolic acids | Phenolic acids | B |
| 321 | Lmnn002886 | 477.14 | 478.15 | C_23_H_26_O_11_ | [M-H]- | 1'-O-β-D-(3,4-dihydroxyphenethyl)-O-caffeoyl-glucoside | Phenolic acids | Phenolic acids | B |
| 322 | Lmqp001565 | 139.04 | 138.03 | C_7_H_6_O_3_ | [M+H]+ | P-Hydroxybenzoic Acid | Phenolic acids | Phenolic acids | B |
| 323 | Lmtn003866 | 147.05 | 148.05 | C_9_H_8_O_2_ | [M-H]- | trans-Cinnamic acid | Phenolic acids | Phenolic acids | B |
| 324 | Lmtp002625 | 165.05 | 164.05 | C_9_H_8_O_3_ | [M+H]+ | p-Hydroxycinnamic acid | Phenolic acids | Phenolic acids | B |
| 325 | Zmhn002227 | 385.11 | 386.12 | C_17_H_22_O_10_ | [M-H]- | Sinapic acid-hexoside | Phenolic acids | Phenolic acids | B |
| 326 | Zmhn002422 | 355.10 | 356.11 | C_16_H_20_O_9_ | [M-H]- | Feruloyl glucose | Phenolic acids | Phenolic acids | B |
| 327 | Zmhn002513 | 385.11 | 386.12 | C_17_H_22_O_10_ | [M-H]- | Isosinapic acid-hexoside | Phenolic acids | Phenolic acids | B |
| 328 | mws0008 | 149.07 | 150.06 | C_9_H_10_O_2_ | [M-H]- | Hydrocinnamic acid | Phenolic acids | Phenolic acids | B |
| 329 | mws0093 | 179.08 | 180.07 | C_10_H_12_O_3_ | [M-H]- | Coniferyl alcohol | Phenolic acids | Phenolic acids | B |
| 330 | mws0182 | 151.00 | 152.04 | C_8_H_8_O_3_ | [M-H]- | p-Hydroxyphenyl acetic acid | Phenolic acids | Phenolic acids | B |
| 331 | mws0885 | 153.00 | 154.02 | C_7_H_6_O_4_ | [M-H]- | 2,4-Dihydroxy benzoic acid | Phenolic acids | Phenolic acids | B |
| 332 | mws1200 | 177.00 | 178.06 | C_10_H_10_O_3_ | [M-H]- | Trans-4-Hydroxycinnamic Acid Methyl Ester | Phenolic acids | Phenolic acids | B |
| 333 | mws1297 | 269.10 | 270.10 | C_13_H_18_O_6_ | [M-H]- | Benzyl β-D-Glucopyranoside | Phenolic acids | Phenolic acids | B |
| 334 | mws1336 | 138.05 | 137.04 | C_7_H_7_NO_2_ | [M+H]+ | 4-Aminobenzoic acid | Phenolic acids | Phenolic acids | B |
| 335 | mws1350 | 181.06 | 182.05 | C_9_H_10_O_4_ | [M-H]- | Syringic Aldehyde | Phenolic acids | Phenolic acids | B |
| 336 | mws1521 | 285.10 | 286.09 | C_13_H_18_O_7_ | [M-H]- | Salicin | Phenolic acids | Phenolic acids | B |
| 337 | mws2212 | 179.04 | 180.04 | C_9_H_8_O_4_ | [M-H]- | Caffeic acid | Phenolic acids | Phenolic acids | B |
| 338 | mws2368 | 137.07 | 138.06 | C_8_H_10_O_2_ | [M-H]- | Tyrosol | Phenolic acids | Phenolic acids | B |
| 339 | pmb0423 | 193.10 | 194.05 | C_10_H_10_O_4_ | [M-H]- | Hydroxy-methoxycinnamate | Phenolic acids | Phenolic acids | B |
| 340 | pmb0752 | 369.10 | 368.09 | C_17_H_20_O_9_ | [M+H]+ | 3-O-Feruloyl quinic acid | Phenolic acids | Phenolic acids | B |
| 341 | pmb2940 | 385.10 | 386.10 | C_17_H_22_O_10_ | [M-H]- | 1-O-β-D-Glucopyranosyl sinapate | Phenolic acids | Phenolic acids | B |
| 342 | pmb3072 | 481.10 | 482.12 | C_22_H_26_O_12_ | [M-H]- | 3-O-p-coumaroyl shikimic acid O-hexoside | Phenolic acids | Phenolic acids | B |
| 343 | pmb3074 | 337.10 | 338.09 | C_16_H_18_O_8_ | [M-H]- | 3-O-p-Coumaroyl quinic acid | Phenolic acids | Phenolic acids | B |
| 344 | pmb3107 | 359.10 | 360.09 | C_15_H_20_O_10_ | [M-H]- | Syringic acid O-glucoside | Phenolic acids | Phenolic acids | B |
| 345 | pme0281 | 165.03 | 166.02 | C_8_H_6_O_4_ | [M-H]- | Terephthalic acid | Phenolic acids | Phenolic acids | B |
| 346 | pmf0440 | 163.10 | 162.06 | C_10_H_10_O_2_ | [M+H]+ | 4-MethoxycinnaMaldehyde | Phenolic acids | Phenolic acids | B |
| 347 | pmn001420 | 341.08 | 342.08 | C_15_H_18_O_9_ | [M-H]- | 1-O-[(E)-Caffeoyl]-β-D-glucopyranose | Phenolic acids | Phenolic acids | B |
| 348 | pmn001517 | 329.10 | 330.11 | C_15_H_22_O_8_ | [M-H]- | 3,4,5-Trimethoxyphenyl-β-D-Glucopyranoside | Phenolic acids | Phenolic acids | B |
| 349 | pmn001518 | 331.10 | 332.06 | C_13_H_16_O_10_ | [M-H]- | 1-O-Galloyl-β-D-glucose | Phenolic acids | Phenolic acids | B |
| 350 | pmn001627 | 331.10 | 332.06 | C_13_H_16_O_10_ | [M-H]- | Glucogallin | Phenolic acids | Phenolic acids | B |
| 351 | pmn001671 | 547.20 | 548.15 | C_23_H_32_O_15_ | [M-H]- | β-D-Furanofructosyl-α-D-(6-mustard acyl)glucoside | Phenolic acids | Phenolic acids | B |
| 352 | pmn001682 | 179.03 | 180.04 | C_9_H_8_O_4_ | [M-H]- | Sorbic acid | Phenolic acids | Phenolic acids | B |
| 353 | pmn001712 | 549.22 | 490.18 | C_22_H_34_O_12_ | [M+CH3COOH-H]- | 3-Hydroxy-4-isopropylbenzylalcohol 3-glucoside-glucoside | Phenolic acids | Phenolic acids | B |
| 354 | pmp000086 | 269.10 | 268.08 | C_13_H_16_O_6_ | [M+H]+ | 1-Feruloyl-sn-glycerol | Phenolic acids | Phenolic acids | B |
| 355 | pmp000087 | 269.10 | 268.08 | C_13_H_16_O_6_ | [M+H]+ | 2-Feruloyl-sn-glycerol | Phenolic acids | Phenolic acids | B |
| 356 | pmp001086 | 127.00 | 126.03 | C_6_H_6_O_3_ | [M+H]+ | 5-Hydroxymethylfurfural | Phenolic acids | Phenolic acids | B |
| 357 | mws0227 | 132.10 | 131.09 | C_6_H_13_NO_2_ | [M+H]+ | L-Leucine | Amino acids and derivatives | Amino acids and derivatives | A |
| 358 | mws0250 | 182.10 | 181.07 | C_9_H_11_NO_3_ | [M+H]+ | L-(-)-Tyrosine | Amino acids and derivatives | Amino acids and derivatives | A |
| 359 | mws0258 | 132.00 | 131.09 | C_6_H_13_NO_2_ | [M+H]+ | L-Isoleucine | Amino acids and derivatives | Amino acids and derivatives | A |
| 360 | mws0260 | 175.00 | 174.10 | C_6_H_14_N_4_O_2_ | [M+H]+ | L-(+)-Arginine | Amino acids and derivatives | Amino acids and derivatives | A |
| 361 | mws0263 | 128.03 | 129.04 | C_5_H_7_NO_3_ | [M-H]- | L-PyroglutamicAcid | Amino acids and derivatives | Amino acids and derivatives | A |
| 362 | mws0282 | 203.00 | 204.08 | C_11_H_12_N_2_O_2_ | [M-H]- | L-Tryptophan | Amino acids and derivatives | Amino acids and derivatives | A |
| 363 | mws0340 | 143.00 | 144.04 | C_6_H_8_O_4_ | [M-H]- | 2,3-Dimethylsuccinic acid | Amino acids and derivatives | Amino acids and derivatives | A |
| 364 | mws0636 | 313.40 | 312.13 | C_18_H_20_N_2_O_3_ | [M+H]+ | Phe-Phe | Amino acids and derivatives | Amino acids and derivatives | A |
| 365 | mws0712 | 130.10 | 131.05 | C_5_H_9_NO_3_ | [M-H]- | N-Propionylglycine | Amino acids and derivatives | Amino acids and derivatives | A |
| 366 | mws0736 | 189.10 | 188.10 | C_8_H_16_N_2_O_3_ | [M+H]+ | N-Glycyl-L-leucine | Amino acids and derivatives | Amino acids and derivatives | A |
| 367 | mws0813 | 128.00 | 129.04 | C_5_H_7_NO_3_ | [M-H]- | 5-Oxoproline | Amino acids and derivatives | Amino acids and derivatives | A |
| 368 | mws1587 | 132.10 | 131.09 | C_6_H_13_NO_2_ | [M+H]+ | α-Aminocaproic acid | Amino acids and derivatives | Amino acids and derivatives | A |
| 369 | mws4176 | 237.12 | 236.10 | C_12_H_16_N_2_O_3_ | [M+H]+ | Alanyl-phenylalanine | Amino acids and derivatives | Amino acids and derivatives | A |
| 370 | mws5035 | 279.17 | 278.15 | C_15_H_22_N_2_O_3_ | [M+H]+ | Leucylphenylalanine | Amino acids and derivatives | Amino acids and derivatives | A |
| 371 | mws5041 | 189.12 | 188.10 | C_8_H_16_N_2_O_3_ | [M+H]+ | Glycylisoleucine | Amino acids and derivatives | Amino acids and derivatives | A |
| 372 | mws5042 | 223.11 | 222.09 | C_11_H_14_N_2_O_3_ | [M+H]+ | Glycylphenylalanine | Amino acids and derivatives | Amino acids and derivatives | A |
| 373 | pme0006 | 116.10 | 115.06 | C_5_H_9_NO_2_ | [M+H]+ | L-Proline | Amino acids and derivatives | Amino acids and derivatives | A |
| 374 | pme0008 | 176.10 | 175.08 | C_6_H_13_N_3_O_3_ | [M+H]+ | L-Citrulline | Amino acids and derivatives | Amino acids and derivatives | A |
| 375 | pme0014 | 148.10 | 147.05 | C_5_H_9_NO_4_ | [M+H]+ | L-Glutamic acid | Amino acids and derivatives | Amino acids and derivatives | A |
| 376 | pme0021 | 166.00 | 165.07 | C_9_H_11_NO_2_ | [M+H]+ | L-Phenylalanine | Amino acids and derivatives | Amino acids and derivatives | A |
| 377 | pme0026 | 147.00 | 146.10 | C_6_H_14_N_2_O_2_ | [M+H]+ | L-(+)-Lysine | Amino acids and derivatives | Amino acids and derivatives | A |
| 378 | pme0193 | 147.07 | 146.06 | C_5_H_10_N_2_O_3_ | [M+H]+ | L-Glutamine | Amino acids and derivatives | Amino acids and derivatives | A |
| 379 | pme1210 | 150.00 | 149.04 | C_5_H_11_NO_2_S | [M+H]+ | L-Methionine | Amino acids and derivatives | Amino acids and derivatives | A |
| 380 | pme3017 | 104.00 | 103.06 | C_4_H_9_NO_2_ | [M+H]+ | 2-Aminoisobutyric acid | Amino acids and derivatives | Amino acids and derivatives | A |
| 381 | pme3033 | 104.00 | 103.06 | C_4_H_9_NO_2_ | [M+H]+ | N,N-Dimethylglycine | Amino acids and derivatives | Amino acids and derivatives | A |
| 382 | pme3193 | 116.00 | 117.04 | C_4_H_7_NO_3_ | [M-H]- | N-Acetylglycine | Amino acids and derivatives | Amino acids and derivatives | A |
| 383 | pme3382 | 160.00 | 161.06 | C_6_H_11_NO_4_ | [M-H]- | N-Acetylthreonine | Amino acids and derivatives | Amino acids and derivatives | A |
| 384 | Qingke_Rfmb319-1-2 | 130.09 | 129.07 | C_6_H_11_NO_2_ | [M+H]+ | Pipecolic acid | Amino acids and derivatives | Amino acids and derivatives | B |
| 385 | Qingke_Rfmb320-1-3 | 144.10 | 143.09 | C_7_H_13_NO_2_ | [M+H]+ | 1,2-N-Methylpipecolic acid | Amino acids and derivatives | Amino acids and derivatives | B |
| 386 | mws0193 | 190.10 | 189.10 | C_7_H_15_N_3_O_3_ | [M+H]+ | L-Homocitrulline | Amino acids and derivatives | Amino acids and derivatives | B |
| 387 | mws0216 | 132.06 | 131.05 | C_5_H_9_NO_3_ | [M+H]+ | Trans-4-Hydroxy-L-proline | Amino acids and derivatives | Amino acids and derivatives | B |
| 388 | mws0219 | 132.00 | 133.03 | C_4_H_7_NO_4_ | [M-H]- | L-AsparticAcid | Amino acids and derivatives | Amino acids and derivatives | B |
| 389 | mws0221 | 241.00 | 240.01 | C_6_H_12_N_2_O_4_S_2_ | [M+H]+ | L-(-)-Cystine | Amino acids and derivatives | Amino acids and derivatives | B |
| 390 | mws0230 | 120.00 | 119.05 | C_4_H_9_NO_3_ | [M+H]+ | L-(-)-Threonine | Amino acids and derivatives | Amino acids and derivatives | B |
| 391 | mws0254 | 156.10 | 155.06 | C_6_H_9_N_3_O_2_ | [M+H]+ | L-Histidine | Amino acids and derivatives | Amino acids and derivatives | B |
| 392 | mws0256 | 118.10 | 117.07 | C_5_H_11_NO_2_ | [M+H]+ | L-Valine | Amino acids and derivatives | Amino acids and derivatives | B |
| 393 | mws0520 | 224.10 | 223.07 | C_11_H_13_NO_4_ | [M+H]+ | N-Acetyl-L-tyrosine | Amino acids and derivatives | Amino acids and derivatives | B |
| 394 | mws0582 | 322.40 | 321.08 | C_11_H_19_N_3_O_6_S | [M+H]+ | S-(methyl)glutathione | Amino acids and derivatives | Amino acids and derivatives | B |
| 395 | mws0629 | 281.30 | 280.09 | C_13_H_16_N_2_O_5_ | [M+H]+ | Asp-phe | Amino acids and derivatives | Amino acids and derivatives | B |
| 396 | mws0805 | 308.10 | 309.09 | C_11_H_19_NO_9_ | [M-H]- | N-Acetylneuraminic acid | Amino acids and derivatives | Amino acids and derivatives | B |
| 397 | mws0890 | 104.03 | 105.04 | C_3_H_7_NO_3_ | [M-H]- | Serine | Amino acids and derivatives | Amino acids and derivatives | B |
| 398 | mws0923 | 200.05 | 199.03 | C_9_H_10_ClNO_2_ | [M+H]+ | L-2-chlorophenylalanine | Amino acids and derivatives | Amino acids and derivatives | B |
| 399 | mws1050 | 148.05 | 147.05 | C_5_H_9_NO_4_ | [M+H]+ | O-Acetylserine | Amino acids and derivatives | Amino acids and derivatives | B |
| 400 | mws1401 | 175.00 | 174.09 | C_7_H_14_N_2_O_3_ | [M+H]+ | L-theanine | Amino acids and derivatives | Amino acids and derivatives | B |
| 401 | mws4134 | 611.15 | 612.12 | C_20_H_32_N_6_O_12_S_2_ | [M-H]- | Oxidized Glutathione | Amino acids and derivatives | Amino acids and derivatives | B |
| 402 | pme0057 | 136.00 | 135.03 | C_4_H_9_NO_2_S | [M+H]+ | DL-Homocysteine | Amino acids and derivatives | Amino acids and derivatives | B |
| 403 | pme0120 | 118.20 | 117.07 | C_5_H_11_NO_2_ | [M+H]+ | 5-Aminovaleric acid | Amino acids and derivatives | Amino acids and derivatives | B |
| 404 | pme0122 | 189.20 | 188.10 | C_8_H_16_N_2_O_3_ | [M+H]+ | N6-Acetyl-L-lysine | Amino acids and derivatives | Amino acids and derivatives | B |
| 405 | pme0124 | 173.20 | 172.08 | C_7_H_12_N_2_O_3_ | [M+H]+ | Glycyl-L-proline | Amino acids and derivatives | Amino acids and derivatives | B |
| 406 | pme0128 | 159.20 | 160.08 | C_6_H_12_N_2_O_3_ | [M-H]- | AlanylAlanine | Amino acids and derivatives | Amino acids and derivatives | B |
| 407 | pme0137 | 187.20 | 188.07 | C_7_H_12_N_2_O_4_ | [M-H]- | N-α-Acetyl-L-glutamine | Amino acids and derivatives | Amino acids and derivatives | B |
| 408 | pme0170 | 217.10 | 216.11 | C_8_H_16_N_4_O_3_ | [M+H]+ | N-α-Acetyl-L-arginine | Amino acids and derivatives | Amino acids and derivatives | B |
| 409 | pme0181 | 170.10 | 169.08 | C_7_H_11_N_3_O_2_ | [M+H]+ | 1-Methylhistidine | Amino acids and derivatives | Amino acids and derivatives | B |
| 410 | pme0253 | 174.10 | 173.09 | C_8_H_15_NO_3_ | [M+H]+ | N-Acetyl-L-leucine | Amino acids and derivatives | Amino acids and derivatives | B |
| 411 | pme0278 | 191.09 | 190.08 | C_7_H_14_N_2_O_4_ | [M+H]+ | 2,6-Diaminooimelic acid | Amino acids and derivatives | Amino acids and derivatives | B |
| 412 | pme1086 | 306.00 | 307.07 | C_10_H_17_N_3_O_6_S | [M-H]- | Glutathione reduced form | Amino acids and derivatives | Amino acids and derivatives | B |
| 413 | pme1228 | 221.10 | 220.08 | C_11_H_12_N_2_O_3_ | [M+H]+ | 5-Hydroxy-L-tryptophan | Amino acids and derivatives | Amino acids and derivatives | B |
| 414 | pme1419 | 164.00 | 199.03 | C_6_H_14_ClNO_2_S | [M-Cl]+ | L-Methionine methyl ester | Amino acids and derivatives | Amino acids and derivatives | B |
| 415 | pme2122 | 112.08 | 111.07 | C_5_H_9_N_3_ | [M+H]+ | Histamine | Amino acids and derivatives | Amino acids and derivatives | B |
| 416 | pme2566 | 217.00 | 218.08 | C_8_H_14_N_2_O_5_ | [M-H]- | (5-L-Glutamyl)-L-amino acid | Amino acids and derivatives | Amino acids and derivatives | B |
| 417 | pme2602 | 184.10 | 185.00 | C_3_H_8_NO_6_P | [M-H]- | O-Phospho-L-serine | Amino acids and derivatives | Amino acids and derivatives | B |
| 418 | pme2617 | 166.00 | 165.04 | C_5_H_11_NO_3_S | [M+H]+ | Methionine sulfoxide | Amino acids and derivatives | Amino acids and derivatives | B |
| 419 | pme2634 | 118.00 | 117.07 | C_5_H_11_NO_2_ | [M+H]+ | DL-Norvaline | Amino acids and derivatives | Amino acids and derivatives | B |
| 420 | pme2743 | 194.10 | 193.07 | C_10_H_11_NO_3_ | [M+H]+ | N-Phenylacetylglycine | Amino acids and derivatives | Amino acids and derivatives | B |
| 421 | pme2758 | 162.00 | 163.04 | C_5_H_9_NO_5_ | [M-H]- | 4-Hydroxy-L-glutamic acid | Amino acids and derivatives | Amino acids and derivatives | B |
| 422 | pme2853 | 172.10 | 173.09 | C_8_H_15_NO_3_ | [M-H]- | Hexanoyl glycine | Amino acids and derivatives | Amino acids and derivatives | B |
| 423 | pme2890 | 269.00 | 268.04 | C_8_H_16_N_2_O_4_S_2_ | [M+H]+ | L-Homocystine | Amino acids and derivatives | Amino acids and derivatives | B |
| 424 | pme2914 | 161.00 | 162.04 | C_6_H_10_O_5_ | [M-H]- | 3-Hydroxy-3-methylpentane-1,5-dioic acid | Amino acids and derivatives | Amino acids and derivatives | B |
| 425 | pme3388 | 189.00 | 188.11 | C_7_H_16_N_4_O_2_ | [M+H]+ | H-HomoArg-OH | Amino acids and derivatives | Amino acids and derivatives | B |
| 426 | pme3827 | 198.20 | 197.06 | C_9_H_11_NO_4_ | [M+H]+ | 3,4-Dihydroxy-DL-phenylalanine | Amino acids and derivatives | Amino acids and derivatives | B |

A^a^: The second-level mass spectrometry and RT of the substance are consistent with the database; B^b^: The parameters of Q1, Q3, RT, DP and CE of the substance are consistent with the database.

**Table S3** 239 differential metabolites and its peak areas of TR in different treatment groups.

| Code | Index | Group | Formula | Compounds | Class I | T-0-1 | T-0-2 | T-0-3 | T-1-1 | T-1-2 | T-1-3 | T-2-1 | T-2-2 | T-2-3 | T-4-1 | T-4-2 | T-4-3 | Level |
| --- | --- | --- | --- | --- | --- | --- | --- | --- | --- | --- | --- | --- | --- | --- | --- | --- | --- | --- |
| 1 | Xigua_mab0299 | 1 | C_36_H_54_O_12_ | Dihydroisocucurbitacin I-Hex | Terpenoids | 57812 | 76704 | 55806 | 13902 | 15492 | 29279 | 52881 | 54592 | 73124 | 27134 | 41295 | 45193 | A^a^ |
| 2 | Xigua_mab0310 | 1 | C_36_H_54_O_12_ | Cucurbitacin D O-glucoside | Terpenoids | 26577 | 52874 | 47872 | 10760 | 12493 | 15878 | 27952 | 16017 | 26929 | 19350 | 20596 | 14894 | A |
| 3 | Xigua_mab0406 | 1 | C_38_H_56_O_13_ | Cucurbitacin D-aceGlu | Terpenoids | 52499 | 70488 | 66917 | 13559 | 7650 | 9466 | 15706 | 17876 | 21220 | 18898 | 29263 | 19993 | A |
| 4 | Xigua_mab0543 | 1 | C_38_H_56_O_13_ | 23,24-Dihydro cucurbitacin E O-glucoside | Terpenoids | 57821 | 54877 | 49580 | 17465 | 10944 | 9419 | 14864 | 27794 | 17952 | 18011 | 30791 | 30047 | A |
| 5 | mws0677 | 1 | C_12_H_14_N_2_O_2_ | N-Acetyl-5-hydroxytryptamine | Alkaloids | 29133 | 29370 | 22867 | 9 | 9 | 9 | 9814 | 3988 | 15949 | 14785 | 10229 | 11235 | B^b^ |
| 6 | pmb0530 | 1 | C_21_H_27_N_7_O_14_P_2_ | Nicotinic acid adenine dinucleotide | Nucleotides and derivatives | 18872 | 23875 | 16441 | 2533 | 7517 | 5408 | 5976 | 8988 | 9022 | 5056 | 8288 | 5513 | B |
| 7 | pmd0130 | 1 | C_22_H_46_NO_7_P | LysoPC 14:0(2n isomer) | Lipids | 40593 | 24436 | 39376 | 8191 | 14441 | 16380 | 11341 | 17803 | 27108 | 22237 | 13993 | 14425 | A |
| 8 | pmn001368 | 1 | C_32_H_44_O_17_ | Olivin Diglucoside | Lignans and Coumarins | 32748 | 30460 | 24888 | 9056 | 8302 | 9089 | 16939 | 16494 | 17982 | 13982 | 15330 | 16000 | B |
| 9 | pmn001370 | 1 | C_32_H_42_O_16_ | Pinoresinol diglucoside | Lignans and Coumarins | 30011 | 32276 | 24740 | 5704 | 7973 | 6536 | 10825 | 10408 | 10927 | 7595 | 14352 | 8329 | B |
| 10 | pmp000898 | 1 | C_36_H_54_O_11_ | Deacetoxycucurbitacin B 3-O-glucoside | Terpenoids | 411020 | 634550 | 486040 | 57088 | 62301 | 60542 | 175670 | 117840 | 199010 | 136570 | 163710 | 130800 | B |
| 11 | pmp000899 | 1 | C_36_H_56_O_12_ | Cucurbitacin F O-glucoside | Terpenoids | 51150 | 106390 | 82399 | 3968 | 6316 | 1729 | 19478 | 16706 | 22410 | 12792 | 20767 | 20829 | B |
| 12 | pmp000901 | 1 | C_38_H_56_O_13_ | Isocucurbitacin B 2-O-glucoside | Terpenoids | 189120 | 156090 | 213050 | 50777 | 55128 | 44207 | 57660 | 83429 | 78694 | 65892 | 76029 | 74559 | B |
| 13 | Hmyn007081 | 2 | C_22_H_43_O_9_P | LysoPG(16:1) | Lipids | 742 | 873 | 473 | 701 | 419 | 509 | 731 | 866 | 617 | 1211 | 1070 | 1393 | B |
| 14 | mws0120 | 2 | C_8_H_20_NO_6_P | Choline alfoscerate | Lipids | 73995 | 77056 | 63706 | 55206 | 59874 | 58992 | 67182 | 82104 | 102640 | 86904 | 154390 | 163060 | B |
| 15 | mws0133 | 2 | C_6_H_6_N_2_O | Nicotinamide | Others | 2551500 | 2615000 | 2531500 | 1548000 | 1877800 | 1747300 | 3249800 | 3195300 | 3161800 | 3819300 | 4364400 | 4003600 | A |
| 16 | mws0458 | 2 | C_8_H_8_O_3_ | Vanillin | Phenolic acids | 1626700 | 1759800 | 1541800 | 981930 | 995610 | 1098100 | 917830 | 1009900 | 1053000 | 3267900 | 4716800 | 4165100 | A |
| 17 | mws1350 | 2 | C_9_H_10_O_4_ | Syringic Aldehyde | Phenolic acids | 15493 | 23630 | 17053 | 20815 | 27790 | 25689 | 31780 | 28863 | 23007 | 88772 | 171440 | 130450 | B |
| 18 | pmp001113 | 2 | C_8_H_8_O_3_ | Dihydro-p-coumarat | Phenolic acids | 7038 | 6207 | 7432 | 5142 | 4644 | 5250 | 6469 | 4395 | 4867 | 15612 | 18477 | 17787 | A |
| 19 | Hmjp006575 | 3 | C_33_H_56_O_14_ | Glyceryl linoleate-O-Hexoside-O-Hexoside 1 | Lipids | 109660 | 132400 | 89767 | 85147 | 71070 | 54702 | 27015 | 29390 | 33160 | 24415 | 20091 | 13549 | A |
| 20 | Hmyn005706 | 3 | C_31_H_58_O_14_ | DMGM(16:0) | Lipids | 2256100 | 2797000 | 3597500 | 1646600 | 1881900 | 1393600 | 741300 | 626520 | 661100 | 570610 | 696060 | 677260 | B |
| 21 | mws0289 | 3 | C_23_H_46_NO_7_P | LysoPE 18:1 | Lipids | 341860 | 392580 | 344550 | 269660 | 237770 | 243460 | 200230 | 177270 | 256220 | 130740 | 158840 | 160760 | A |
| 22 | pmb0296 | 3 | C_21_H_40_O_4_ | MAG(18:1)isomer2 | Lipids | 496890 | 532110 | 566580 | 274520 | 306670 | 264550 | 156330 | 156240 | 172920 | 131620 | 9 | 9 | A |
| 23 | pmb0876 | 3 | C_21_H_44_NO_7_P | LysoPE 16:0 | Lipids | 14003000 | 14649000 | 11775000 | 9878700 | 8885600 | 7770000 | 5571100 | 5621900 | 6216400 | 5440200 | 6130000 | 5503500 | A |
| 24 | pmb2260 | 3 | C_23_H_46_NO_7_P | LysoPC 15:1 | Lipids | 26650 | 27061 | 32164 | 18696 | 20257 | 9 | 9 | 9 | 9 | 9 | 9 | 9 | A |
| 25 | pmb2363 | 3 | C_21_H_40_O_4_ | MAG(18:1)isomer1 | Lipids | 449700 | 400910 | 390650 | 205720 | 202070 | 189920 | 79076 | 95350 | 95656 | 85184 | 71644 | 56154 | A |
| 26 | pme0120 | 3 | C_5_H_11_NO_2_ | 5-Aminovaleric acid | Amino acids and derivatives | 215730 | 235510 | 251810 | 187470 | 139660 | 172440 | 142620 | 112990 | 80625 | 113970 | 113850 | 137810 | B |
| 27 | pme0490 | 3 | C_6_H_5_NO_2_ | Nicotinic acid | Others | 96566 | 96784 | 105320 | 74216 | 88118 | 99953 | 59058 | 63656 | 70121 | 47931 | 44928 | 44541 | A |
| 28 | pme3011 | 3 | C_4_H_9_NO_2_ | γ-Aminobutyric acid | Organic acids | 35289000 | 35473000 | 34457000 | 24616000 | 24164000 | 22232000 | 18005000 | 17358000 | 19594000 | 15704000 | 16263000 | 16640000 | B |
| 29 | pme3033 | 3 | C_4_H_9_NO_2_ | N,N-Dimethylglycine | Amino acids and derivatives | 13170000 | 12560000 | 12772000 | 9084200 | 8862500 | 8342400 | 6598100 | 6488900 | 7030400 | 5833900 | 6172400 | 6336300 | A |
| 30 | pme3337 | 3 | C_14_H_17_N_5_O_8_ | N6-Succinyl Adenosine | Nucleotides and derivatives | 1365800 | 1403100 | 1447500 | 749190 | 799780 | 929840 | 604760 | 574750 | 521740 | 521560 | 577000 | 543010 | A |
| 31 | pmn001671 | 3 | C_23_H_32_O_15_ | β-D-Furanofructosyl-α-D-(6-mustard acyl)glucoside | Phenolic acids | 22546 | 17899 | 9985 | 15391 | 7833 | 12243 | 5937 | 8120 | 9458 | 4985 | 7128 | 3148 | B |
| 32 | pmp001248 | 3 | C_20_H_30_NO_9_+ | Caffeoylcholine 5-glucoside | Alkaloids | 17079 | 12442 | 14546 | 13319 | 10694 | 10334 | 7653 | 8380 | 4972 | 8426 | 8949 | 9961 | B |
| 33 | pmp001275 | 3 | C_31_H_61_O_14_N | 3-Hydroxypropyl palmitate glc-glucosamine | Alkaloids | 11611000 | 11132000 | 11617000 | 7411000 | 6804300 | 5447700 | 3434700 | 3190900 | 3485700 | 3068400 | 3206500 | 2928800 | A |
| 34 | pmp001276 | 3 | C_33_H_56_O_14_ | 2,3-Dihydroxypropyl-9,12,15-octadecatrienoate-hexose-hexose | Lipids | 4634200 | 4135600 | 4707500 | 2906500 | 2606900 | 2576700 | 1149900 | 1446600 | 1432700 | 1043600 | 994920 | 1182100 | A |
| 35 | pmp001277 | 3 | C_21_H_44_NO_7_P | 3-{(2-Aminoethoxy)(hydroxy)phosphoryl]oxy}-2-hydroxypropyl palmitate | Alkaloids | 14182000 | 14780000 | 14337000 | 9112900 | 8893900 | 7513200 | 5491100 | 4971100 | 6479900 | 5893200 | 6024200 | 5227200 | A |
| 36 | pmp001282 | 3 | C_28_H_50_NO_7_P | Propyl2-(trimethylammonio)ethyl phosphate | Others | 59530 | 62335 | 54656 | 37797 | 33844 | 9 | 9 | 9 | 9 | 9 | 9 | 9 | B |
| 37 | Hmyn007168 | 4 | C_22_H_45_O_9_P | LysoPG(16:0) | Lipids | 1132700 | 1094200 | 1033300 | 2042800 | 2098100 | 2673200 | 2445200 | 2405200 | 2822600 | 3008700 | 3636600 | 3629900 | B |
| 38 | Lmhn002926 | 4 | C_13_H_12_O_7_ | p-coumaroylmalic acid | Phenolic acids | 9 | 9 | 9 | 14152 | 13741 | 25128 | 17697 | 21320 | 24630 | 29103 | 29531 | 28236 | B |
| 39 | mws0024 | 4 | C_7_H_6_O_5_ | Gallic acid | Flavonoids | 9 | 9 | 9 | 24274 | 21757 | 23104 | 20904 | 34087 | 45705 | 76922 | 45565 | 38677 | B |
| 40 | mws0180 | 4 | C_7_H_6_O_4_ | 2,5-Dihydroxybenzoic acid | Phenolic acids | 9 | 9 | 9 | 19034000 | 19123000 | 18863000 | 15498000 | 17398000 | 19194000 | 28769000 | 32543000 | 25560000 | A |
| 41 | mws0183 | 4 | C_7_H_6_O_4_ | Protocatechuic acid | Flavonoids | 9 | 9 | 9 | 18075000 | 18843000 | 18241000 | 15821000 | 18073000 | 20137000 | 28647000 | 31433000 | 26229000 | A |
| 42 | mws0219 | 4 | C_4_H_7_NO_4_ | L-AsparticAcid | Amino acids and derivatives | 1287300 | 1343400 | 1311300 | 3928500 | 4289100 | 3753900 | 4091300 | 4202900 | 4200300 | 6581900 | 6083600 | 6267800 | B |
| 43 | mws0255 | 4 | C_4_H_5_N_3_O | Cytosine | Nucleotides and derivatives | 244630 | 255610 | 246860 | 601020 | 656640 | 799890 | 1020700 | 1008000 | 1057800 | 1544000 | 1394200 | 1345700 | A |
| 44 | mws0572 | 4 | C_5_H_7_N_3_O | 5-Methylcytosine | Nucleotides and derivatives | 9 | 9 | 9 | 32027 | 47972 | 43895 | 67540 | 67469 | 97330 | 81630 | 88711 | 68447 | B |
| 45 | mws0609 | 4 | C_10_H_12_N_5_O_7_P | Guanosine 3',5'-cyclic monophosphate | Nucleotides and derivatives | 704230 | 515200 | 402060 | 5235800 | 4978900 | 4256900 | 10298000 | 8556600 | 8156800 | 7197100 | 7976900 | 6916500 | B |
| 46 | mws0636 | 4 | C_18_H_20_N_2_O_3_ | Phe-Phe | Amino acids and derivatives | 9 | 9 | 6502 | 519190 | 478310 | 478800 | 656130 | 636930 | 625870 | 903670 | 934660 | 858080 | A |
| 47 | mws0639 | 4 | C_7_H_6_O_4_ | 2,3-Dihydroxybenzoic Acid | Organic acids | 9 | 9 | 9 | 18723000 | 18056000 | 17831000 | 15253000 | 17123000 | 18982000 | 27684000 | 30729000 | 24912000 | B |
| 48 | mws0847 | 4 | C_6_H_7_N_5_ | 1-Methyladenine | Nucleotides and derivatives | 87396 | 73016 | 63052 | 74098 | 115160 | 70539 | 154750 | 139440 | 225050 | 195940 | 211120 | 215400 | A |
| 49 | mws0885 | 4 | C_7_H_6_O_4_ | 2,4-Dihydroxy benzoic acid | Phenolic acids | 9 | 9 | 9 | 18233000 | 18193000 | 16397000 | 14634000 | 17652000 | 18260000 | 27618000 | 30120000 | 24046000 | B |
| 50 | mws4176 | 4 | C_12_H_16_N_2_O_3_ | Alanyl-phenylalanine | Amino acids and derivatives | 41241 | 42021 | 51318 | 2201000 | 2617700 | 2528300 | 3204800 | 3293800 | 3749800 | 5622300 | 5763700 | 5034600 | A |
| 51 | mws5035 | 4 | C_15_H_22_N_2_O_3_ | Leucylphenylalanine | Amino acids and derivatives | 40697 | 34847 | 36988 | 6116100 | 6865500 | 6917000 | 8707900 | 8490500 | 8263200 | 15157000 | 16400000 | 14845000 | A |
| 52 | pmb0484 | 4 | C_5_H_13_NO | Choline | Alkaloids | 219190 | 124760 | 170210 | 306380 | 370790 | 236020 | 792220 | 687670 | 930440 | 956920 | 881500 | 879020 | B |
| 53 | pmb0752 | 4 | C_17_H_20_O_9_ | 3-O-Feruloyl quinic acid | Phenolic acids | 2666 | 1390 | 2116 | 11283 | 17938 | 11574 | 12821 | 13468 | 22573 | 17487 | 24043 | 15226 | B |
| 54 | pmb0856 | 4 | C_23_H_46_NO_7_P | LysoPE 18:1(2n isomer) | Lipids | 87098 | 67705 | 42770 | 114670 | 97078 | 168600 | 217710 | 177350 | 248690 | 197610 | 269910 | 312110 | B |
| 55 | pmb0863 | 4 | C_24_H_46_NO_7_P | LysoPC 16:2(2n isomer) | Lipids | 25636 | 21642 | 23723 | 248450 | 271160 | 424000 | 582810 | 494160 | 873560 | 615170 | 883670 | 906420 | A |
| 56 | pmb0865 | 4 | C_26_H_48_NO_7_P | LysoPC 18:3(2n isomer) | Lipids | 3570300 | 3432600 | 3150000 | 30314000 | 33192000 | 40216000 | 51275000 | 47288000 | 58526000 | 53378000 | 64094000 | 64038000 | A |
| 57 | pmb0998 | 4 | C_10_H_14_N_5_O_8_P | Guanosine 5'-monophosphate | Nucleotides and derivatives | 3528 | 5798 | 9062 | 15366 | 18748 | 31371 | 58238 | 76901 | 125200 | 34080 | 55763 | 59488 | B |
| 58 | pmb2792 | 4 | C_18_H_30_O_3_ | 13-HOTrE(r) | Lipids | 29959 | 32866 | 44376 | 155620 | 83292 | 133530 | 158200 | 234050 | 120420 | 168420 | 194370 | 158010 | A |
| 59 | pmb2922 | 4 | C_15_H_24_N_2_O_17_P_2_ | Uridine 5'-diphospho-D-glucose | Nucleotides and derivatives | 42526 | 33209 | 35043 | 109990 | 121050 | 151340 | 281480 | 225590 | 366700 | 332690 | 371990 | 390650 | A |
| 60 | pmb3041 | 4 | C_23_H_22_O_14_ | Tricin O-saccharic acid | Flavonoids | 9 | 9 | 9 | 71887 | 89569 | 109920 | 257630 | 154030 | 247660 | 89628 | 95230 | 99202 | B |
| 61 | pmb3079 | 4 | C_8_H_16_NO_9_P | N-Acetyl-D-glucosamine 1-phosphate | Others | 72045 | 59952 | 61110 | 79782 | 96913 | 83717 | 182990 | 148350 | 136590 | 197450 | 185540 | 164290 | B |
| 62 | pme0014 | 4 | C_5_H_9_NO_4_ | L-Glutamic acid | Amino acids and derivatives | 394510 | 459900 | 499780 | 1145700 | 1012200 | 1233000 | 1490400 | 1531200 | 1475200 | 1744200 | 1478100 | 1650900 | A |
| 63 | pme1194 | 4 | C_9_H_13_N_3_O_4_ | Deoxycytidine | Nucleotides and derivatives | 9 | 9 | 9 | 298760 | 421970 | 505360 | 595360 | 623660 | 657330 | 665420 | 646000 | 643140 | B |
| 64 | pme1210 | 4 | C_5_H_11_NO_2_S | L-Methionine | Amino acids and derivatives | 3027 | 3814 | 5663 | 3803 | 6613 | 4597 | 11761 | 11807 | 18475 | 13272 | 13725 | 15645 | A |
| 65 | pme1419 | 4 | C_6_H_14_ClNO_2_S | L-Methionine methyl ester | Amino acids and derivatives | 10228 | 9 | 9 | 9 | 9380 | 9 | 17287 | 17780 | 30125 | 24431 | 28772 | 24658 | B |
| 66 | pme2122 | 4 | C_5_H_9_N_3_ | Histamine | Amino acids and derivatives | 153700 | 154580 | 152950 | 376650 | 469110 | 461190 | 640590 | 635420 | 675060 | 877100 | 796010 | 860080 | B |
| 67 | pme2890 | 4 | C_8_H_16_N_2_O_4_S_2_ | L-Homocystine | Amino acids and derivatives | 15533 | 8563 | 11509 | 384870 | 341690 | 352080 | 441040 | 412830 | 400580 | 614950 | 614410 | 622080 | B |
| 68 | pme2993 | 4 | C_10_H_8_O_4_ | Scopoletin(7-Hydroxy-5-methoxycoumarin) | Lignans and Coumarins | 11066 | 13156 | 16449 | 20333 | 23796 | 22049 | 47999 | 30259 | 86266 | 26141 | 21250 | 20207 | B |
| 69 | pme3081 | 4 | C_8_H_12_N_2_O_2_ | 4-(Aminomethyl)-5-(hydroxymethyl)-2-methylpyridin-3-ol | Others | 120420 | 118440 | 95514 | 114090 | 114190 | 93304 | 232030 | 194200 | 285700 | 259780 | 198330 | 200120 | B |
| 70 | pme3188 | 4 | C_9_H_13_N_2_O_9_P | Uridine 5'-monophosphate | Nucleotides and derivatives | 147160 | 127110 | 121990 | 102370 | 82245 | 105390 | 238580 | 225960 | 231780 | 221280 | 210170 | 183590 | B |
| 71 | pme3732 | 4 | C_9_H_13_N_3_O_5_ | Cytidine | Nucleotides and derivatives | 4269900 | 4546200 | 4833700 | 13035000 | 13718000 | 14983000 | 20328000 | 20881000 | 20159000 | 28126000 | 27507000 | 27284000 | B |
| 72 | pmp000628 | 4 | C_15_H_20_N_2_O_2_ | 9α-Hydroxysophoramine | Alkaloids | 9 | 9 | 9 | 186150 | 190870 | 173140 | 182460 | 175960 | 212660 | 296210 | 348010 | 340480 | B |
| 73 | pmp001251 | 4 | C_26_H_51_NO_7_P+ | PC(18:2)isomer | Lipids | 465800 | 431250 | 388510 | 3942500 | 4350800 | 5538800 | 7888100 | 6993800 | 9301400 | 8607000 | 10578000 | 11984000 | A |
| 74 | pmp001269 | 4 | C_18_H_39_NO | Hexadecyl ethanolamine | Others | 9 | 9 | 9 | 9 | 9 | 16095 | 34112 | 12903 | 18958 | 22731 | 13183 | 12354 | B |
| 75 | pmp001281 | 4 | C_26_H_52_NO_7_P | LysoPC(18:1) | Lipids | 955600 | 886280 | 776510 | 4040600 | 4639300 | 5956900 | 8154500 | 7579100 | 9469600 | 10219000 | 13846000 | 14345000 | A |
| 76 | mws0749 | 5 | C_7_H_8_O_2_ | 4-Hydroxybenzyl alcohol | Phenolic acids | 68026 | 65375 | 54772 | 9 | 9 | 9 | 9 | 9 | 9 | 9 | 9 | 9 | B |
| 77 | Lmlp002133 | 5 | C_9_H_8_O_3_ | Trans-p-Hydroxycinnamic acid | Phenolic acids | 1552200 | 1429400 | 1343100 | 187590 | 183270 | 179490 | 107450 | 100290 | 105180 | 92015 | 83861 | 74611 | B |
| 78 | Lmtn003866 | 5 | C_9_H_8_O_2_ | trans-Cinnamic acid | Phenolic acids | 590270 | 675530 | 551610 | 31842 | 27553 | 38882 | 35062 | 33577 | 33026 | 6787 | 7529 | 9796 | B |
| 79 | Lmtp002625 | 5 | C_9_H_8_O_3_ | p-Hydroxycinnamic acid | Phenolic acids | 1502600 | 1410000 | 1216000 | 183990 | 168680 | 170040 | 107100 | 99489 | 107120 | 79976 | 77337 | 79972 | B |
| 80 | Qingke_Rfmb089-2-3 | 5 | C_18_H_34_O_4_ | 9,10-Dihydroxy-12-octadecenoic acid | Lipids | 138630 | 130280 | 123160 | 19851 | 19510 | 16141 | 12215 | 11237 | 14747 | 15191 | 16076 | 18240 | A |
| 81 | Qingke_Rfmb090-1-3 | 5 | C_18_H_32_O_3_ | 13-Hydroxy-9,11-octadecadienoic acid | Lipids | 5693100 | 5800600 | 5546400 | 1962900 | 1931800 | 1740200 | 1485000 | 1312600 | 1827200 | 1560400 | 2032200 | 1974200 | A |
| 82 | Qingke_Rfmb091-1-1 | 5 | C_18_H_32_O_3_ | 9-Hydroxy-10,12-octadecadienoic acid | Lipids | 5780900 | 6082000 | 6000300 | 2182700 | 2057000 | 1829100 | 1596600 | 1394600 | 1890100 | 1603800 | 2116700 | 2026700 | A |
| 83 | Xigua_cub060 | 5 | C_32_H_46_O_8_ | 23,24-Dihydro cucurbitacin E | Terpenoids | 456120 | 266570 | 398030 | 63772 | 52086 | 59558 | 39643 | 45529 | 64268 | 48446 | 70828 | 48037 | B |
| 84 | Zmhn001926 | 5 | C_13_H_16_O_8_ | Salicylic acid O-glycoside | Phenolic acids | 7895000 | 7442400 | 8064100 | 1621100 | 1656500 | 1383300 | 1351600 | 1391400 | 1393500 | 1391500 | 1685200 | 1505400 | A |
| 85 | Zmhn002301 | 5 | C_15_H_18_O_8_ | p-Coumaric acid-O-glycoside | Phenolic acids | 836440 | 887310 | 852680 | 285360 | 302680 | 267200 | 274030 | 288220 | 315760 | 250490 | 236110 | 243960 | A |
| 86 | Zmhn002422 | 5 | C_16_H_20_O_9_ | Feruloyl glucose | Phenolic acids | 128540 | 130770 | 156950 | 45474 | 50124 | 46309 | 41443 | 51853 | 43299 | 49207 | 53059 | 44692 | B |
| 87 | Zmxn001997 | 5 | C_13_H_16_O_8_ | isosalicylic acid O-glycoside | Phenolic acids | 7331600 | 6795500 | 7506700 | 1530500 | 1429100 | 1295900 | 1272800 | 1291500 | 1248300 | 1268100 | 1491200 | 1489900 | A |
| 88 | mws0407 | 5 | C_30_H_44_O_7_ | Cucurbitacin D | Terpenoids | 138010 | 130010 | 133850 | 9 | 9 | 9 | 9 | 9 | 9 | 9 | 9 | 9 | B |
| 89 | mws0008 | 5 | C_9_H_10_O_2_ | Hydrocinnamic acid | Phenolic acids | 66058 | 87268 | 53717 | 11506 | 5867 | 13103 | 19774 | 16842 | 8656 | 11672 | 10821 | 7767 | B |
| 90 | mws0072 | 5 | C_21_H_20_O_10_ | Apigenin 5-O-glucoside | Flavonoids | 11202 | 10498 | 9448 | 3391 | 2804 | 2364 | 4536 | 3339 | 1162 | 4645 | 2441 | 1036 | B |
| 91 | mws0237 | 5 | C_9_H_16_O_4_ | Anchoic Acid | Organic acids | 32305000 | 33472000 | 31156000 | 12214000 | 11501000 | 11907000 | 9964100 | 10815000 | 10103000 | 12318000 | 14924000 | 12425000 | A |
| 92 | mws0264 | 5 | C12H22O11 | D-(+)-TrehaloseAnhydrous | Others | 1422600 | 955750 | 1322300 | 402280 | 421100 | 396950 | 336310 | 333890 | 298180 | 316310 | 296650 | 301260 | A |
| 93 | mws0282 | 5 | C_11_H_12_N_2_O_2_ | L-Tryptophan | Amino acids and derivatives | 8357700 | 7752700 | 7937800 | 162720 | 177570 | 82837 | 1167800 | 467650 | 1759200 | 1566900 | 976460 | 1278900 | A |
| 94 | mws0376 | 5 | C_4_H_4_O_4_ | Fumaric acid | Organic acids | 914350 | 1001100 | 1023800 | 54799 | 56432 | 61217 | 60288 | 41929 | 41480 | 53212 | 39241 | 24462 | A |
| 95 | mws0474 | 5 | C_12_H_22_O_4_ | Dodecanedioic aicd | Organic acids | 13223 | 12057 | 10750 | 2068 | 3056 | 2779 | 2594 | 3068 | 1592 | 3338 | 3688 | 3990 | A |
| 96 | mws0489 | 5 | C_8_H_6_O_3_ | Benzoylformic acid | Organic acids | 183120 | 182490 | 202200 | 24310 | 31734 | 27944 | 25106 | 19547 | 24144 | 27317 | 35035 | 28555 | B |
| 97 | mws0520 | 5 | C_11_H_13_NO_4_ | N-Acetyl-L-tyrosine | Amino acids and derivatives | 116970 | 114610 | 107070 | 31303 | 42364 | 31395 | 30615 | 29424 | 33680 | 28187 | 32366 | 24340 | B |
| 98 | mws0567 | 5 | C_5_H_11_N_3_O_2_ | 4-Guanidinobutyric acid | Organic acids | 1242000 | 1177100 | 1122200 | 524960 | 555790 | 661470 | 577440 | 593360 | 522930 | 559560 | 548330 | 495840 | B |
| 99 | mws0601 | 5 | C_5_H_5_NO_2_ | Pyrrole-2-carboxylic acid | Others | 527630 | 551700 | 517860 | 101800 | 112930 | 105590 | 98691 | 97848 | 118150 | 89531 | 106940 | 98130 | B |
| 100 | mws0972 | 5 | C_6_H_12_O_3_ | 5-Hydroxyhexanoic acid | Organic acids | 196190 | 182020 | 184820 | 61768 | 63106 | 52368 | 54797 | 59520 | 43799 | 75747 | 76230 | 63649 | B |
| 101 | mws1050 | 5 | C_5_H_9_NO_4_ | O-Acetylserine | Amino acids and derivatives | 346270 | 365220 | 376390 | 145760 | 135850 | 131440 | 120280 | 145290 | 112830 | 108010 | 146810 | 160130 | B |
| 102 | mws1060 | 5 | C_10_H_12_N_4_O_5_ | 9-(β-D-Arabinofuranosyl) hypoxanthine | Nucleotides and derivatives | 2972500 | 2983100 | 2342300 | 298100 | 327240 | 274730 | 306130 | 275230 | 289550 | 256560 | 293610 | 288620 | A |
| 103 | mws1080 | 5 | C_12_H_22_O_11_ | Galactinol | Others | 7485800 | 6200900 | 6289900 | 1771600 | 1706700 | 1734900 | 1433400 | 1543700 | 1444200 | 1354900 | 1453100 | 1219600 | A |
| 104 | mws1200 | 5 | C_10_H_10_O_3_ | Trans-4-Hydroxycinnamic Acid Methyl Ester | Phenolic acids | 26779 | 25224 | 22151 | 4497 | 3530 | 4701 | 3554 | 2078 | 5701 | 3155 | 4861 | 4600 | B |
| 105 | mws2213 | 5 | C_9_H_8_O_2_ | Cinnamic acid | Phenolic acids | 1379100 | 1487000 | 1112500 | 80352 | 68143 | 99685 | 72456 | 82309 | 76964 | 21706 | 21045 | 25387 | A |
| 106 | mws4134 | 5 | C_20_H_32_N_6_O_12_S_2_ | Oxidized Glutathione | Amino acids and derivatives | 93412 | 77668 | 91060 | 2232 | 4374 | 2635 | 5780 | 2758 | 1544 | 3595 | 1923 | 1656 | B |
| 107 | mws4163 | 5 | C_24_H_42_O_21_ | 1,1-Kestotetraose | Others | 352600 | 500210 | 360150 | 105660 | 105860 | 98454 | 116090 | 91347 | 80675 | 101040 | 83768 | 70745 | B |
| 108 | mws5040 | 5 | C_12_H_21_O_11_Na | Turanose | Others | 118520 | 135270 | 125000 | 9 | 9 | 9 | 9 | 9 | 9 | 9 | 9 | 9 | B |
| 109 | mws5045 | 5 | C_12_H_24_O_3_ | 12-Hydroxydodecanoic acid | Lipids | 126810 | 115590 | 98402 | 24093 | 24966 | 28569 | 23700 | 28644 | 28352 | 29947 | 35698 | 37771 | A |
| 110 | pmb0492 | 5 | C_34_H_37_N_3_O_6_ | N',N'',N'''-p-Coumaroyl-cinnamoyl-caffeoyl spermidine | Alkaloids | 491740 | 500080 | 442710 | 12246 | 11426 | 14233 | 11143 | 11139 | 13995 | 12392 | 13813 | 15171 | B |
| 111 | pmb0889 | 5 | C_18_H_30_O_2_ | Punicic acid | Lipids | 869390 | 844890 | 885030 | 293310 | 303430 | 281790 | 226780 | 223750 | 266140 | 251160 | 288480 | 265690 | A |
| 112 | pmb2325 | 5 | C_21_H_36_O_4_ | MAG(18:3)isomer2 | Lipids | 115300 | 113080 | 114030 | 34548 | 43024 | 40480 | 33534 | 35699 | 51120 | 28571 | 37100 | 38395 | A |
| 113 | pmb2778 | 5 | C_18_H_32_O_3_ | 9,10-EODE | Lipids | 8135200 | 8759100 | 8298300 | 2640400 | 2406000 | 2454700 | 1994600 | 1858200 | 2252400 | 2138700 | 2415800 | 2414200 | A |
| 114 | pmb2784 | 5 | C_18_H_30_O_3_ | 13-HOTrE | Lipids | 21547 | 23436 | 23603 | 4715 | 4806 | 5701 | 4959 | 4813 | 4867 | 4595 | 4331 | 4360 | A |
| 115 | pmb2786 | 5 | C_18_H_30_O_3_ | 9-HOTrE | Lipids | 925660 | 979260 | 918720 | 399910 | 379550 | 323310 | 261570 | 258230 | 339110 | 324810 | 379110 | 371730 | A |
| 116 | pmb2787 | 5 | C_18_H_30_O_3_ | 9-KODE | Lipids | 91279 | 91807 | 74327 | 29256 | 30722 | 36105 | 21548 | 21540 | 38620 | 21983 | 31225 | 25813 | A |
| 117 | pmb3894 | 5 | C_17_H_14_O_7_ | Di-O-methylquercetin | Flavonoids | 12297000 | 12227000 | 12164000 | 3141900 | 2873500 | 2871900 | 1862800 | 1768200 | 2309900 | 1842200 | 2599300 | 2484200 | B |
| 118 | pme0170 | 5 | C_8_H_16_N_4_O_3_ | N-α-Acetyl-L-arginine | Amino acids and derivatives | 1248500 | 1211500 | 1320900 | 244070 | 258230 | 181780 | 192810 | 247850 | 206830 | 170920 | 192350 | 210790 | B |
| 119 | pme0253 | 5 | C_8_H_15_NO_3_ | N-Acetyl-L-leucine | Amino acids and derivatives | 271320 | 232100 | 242240 | 50453 | 52712 | 53774 | 42235 | 43914 | 45624 | 36302 | 42289 | 35267 | B |
| 120 | pmp000896 | 5 | C_32_H_46_O_8_ | cucurbitacin B | Terpenoids | 356690 | 308040 | 217980 | 9 | 9 | 9 | 9 | 9 | 9 | 9 | 9 | 9 | B |
| 121 | pme0295 | 5 | C_6_H_11_NO_3_ | 4-Acetamidobutyric acid | Organic acids | 2218400 | 2226900 | 2324000 | 294480 | 288280 | 319360 | 175730 | 173440 | 191910 | 92687 | 117660 | 101220 | B |
| 122 | pme0500 | 5 | C_18_H_32_O_16_ | D-(+)-Melezitose | Others | 226140 | 268160 | 228260 | 87349 | 81093 | 100630 | 71944 | 85085 | 63423 | 73545 | 76308 | 81774 | B |
| 123 | pme0519 | 5 | C_12_H_22_O_11_ | D-(+)-Sucrose | Others | 5049900 | 3658300 | 3836600 | 867610 | 786110 | 845740 | 669680 | 707920 | 672790 | 653810 | 684550 | 655970 | A |
| 124 | pme1109 | 5 | C_5_H_5_N_5_O | Guanine | Nucleotides and derivatives | 1585000 | 1373400 | 1335200 | 398740 | 319620 | 332530 | 311480 | 230200 | 432140 | 233280 | 229030 | 249990 | A |
| 125 | pme1137 | 5 | C_6_H_5_NO_3_ | 6-Hydroxynicotinic acid | Alkaloids | 24196 | 42610 | 32317 | 11106 | 9681 | 11581 | 7359 | 9127 | 9106 | 10848 | 12323 | 9553 | B |
| 126 | pme1173 | 5 | C_5_H_4_N_4_O | Allopurinol | Nucleotides and derivatives | 547830 | 485870 | 472610 | 229640 | 247080 | 260100 | 241630 | 247700 | 251110 | 248360 | 261540 | 244990 | B |
| 127 | pme1439 | 5 | C_9_H_8_O_3_ | p-Coumaric acid | Phenolic acids | 4700300 | 4414300 | 3981000 | 568550 | 524270 | 541150 | 348700 | 283390 | 342020 | 244950 | 227750 | 198810 | A |
| 128 | pme2049 | 5 | C_4_H_8_O_3_ | 2-Hydroxybutanoic acid | Organic acids | 464670 | 507390 | 532390 | 214080 | 204700 | 258410 | 214240 | 267210 | 273150 | 278600 | 309260 | 220380 | B |
| 129 | pme2266 | 5 | C_10_H_16_N_2_O_3_S | Biotin | Others | 18398 | 31456 | 30362 | 6618 | 11397 | 5829 | 8637 | 9317 | 10607 | 8583 | 7685 | 10848 | B |
| 130 | pme2987 | 5 | C_9_H_8_O_2_ | 3,4-Dihydrocoumarin | Lignans and Coumarins | 64137 | 86525 | 78000 | 3659 | 3621 | 4738 | 5120 | 6485 | 3334 | 493 | 1706 | 565 | B |
| 131 | pme3193 | 5 | C_4_H_7_NO_3_ | N-Acetylglycine | Amino acids and derivatives | 16256 | 19433 | 19309 | 9 | 9 | 9 | 9 | 9 | 9 | 9 | 9 | 9 | A |
| 132 | pme3207 | 5 | C_6_H_6_O_4_ | Trans,trans-Muconic acid | Organic acids | 91377 | 95885 | 105170 | 42397 | 44942 | 46903 | 45411 | 44931 | 35174 | 58512 | 55663 | 52829 | B |
| 133 | pmn001429 | 5 | C_32_H_46_O_9_ | Cucurbitacin A | Terpenoids | 259620 | 291800 | 316190 | 28467 | 30072 | 31905 | 22143 | 21271 | 26248 | 22103 | 24160 | 16316 | B |
| 134 | pmn001433 | 5 | C_30_H_46_O_7_ | Cucurbitacin F | Terpenoids | 5191100 | 5544600 | 5155300 | 1042900 | 1214200 | 1163600 | 925600 | 948340 | 1211500 | 1064100 | 986900 | 977660 | B |
| 135 | pmn001492 | 5 | C_11_H_8_O_3_ | Ayapin | Lignans and Coumarins | 1498200 | 1639800 | 1453000 | 495790 | 514570 | 536160 | 422040 | 443760 | 436590 | 508640 | 650780 | 502610 | A |
| 136 | pmn001495 | 5 | C_19_H_38_O_4_ | Hexadecanoic acid 2,3-dihydroxypropyl ester | Lipids | 12201000 | 12447000 | 11975000 | 3121100 | 2820800 | 2790300 | 1774600 | 1705100 | 2303900 | 1837300 | 2571300 | 2494700 | A |
| 137 | pmn001517 | 5 | C_15_H_22_O_8_ | 3,4,5-Trimethoxyphenyl-β-D-Glucopyranoside | Phenolic acids | 5045600 | 5954400 | 5523500 | 1115000 | 1130900 | 1251100 | 1580600 | 1412400 | 1456000 | 1219200 | 1512800 | 1373400 | B |
| 138 | pmn001668 | 5 | C_22_H_24_O_8_ | Apigenin-3-O-α-L-rhamnoside | Flavonoids | 39593 | 46495 | 32066 | 9016 | 13760 | 12644 | 15242 | 12991 | 9962 | 14037 | 8890 | 14042 | B |
| 139 | pmn001688 | 5 | C_18_H_32_O_3_ | 9S-Hyroxy-10E,12E-octadecadienoic acid | Lipids | 5663800 | 5909000 | 5846000 | 2123900 | 2047200 | 1883100 | 1573200 | 1411200 | 2004000 | 1665900 | 2126900 | 1993300 | A |
| 140 | pmn001689 | 5 | C_18_H_32_O_4_ | 9-Hydroxy-12-oxo-10-octadecenoic acid | Lipids | 244200 | 226050 | 227960 | 41004 | 37517 | 39824 | 30197 | 30262 | 36320 | 37645 | 44012 | 46880 | A |
| 141 | pmn001691 | 5 | C_18_H_32_O_5_ | 9,12,13-Trihyroxy-10,15-octadecadienoic acid | Lipids | 944150 | 953700 | 902620 | 143760 | 132380 | 134160 | 61584 | 62423 | 88945 | 80504 | 118280 | 102370 | A |
| 142 | pmn001694 | 5 | C_18_H_34_O_5_ | 9,10,13-Trihyroxy-11-octadecadienoic acid | Lipids | 12610000 | 12896000 | 12201000 | 3277200 | 2955200 | 2981000 | 1832800 | 1717300 | 2434900 | 1888800 | 2733400 | 2719100 | A |
| 143 | pmp000270 | 5 | C_30_H_44_O_6_ | Lup-12-en-15α,19β-diol-3,11-dioxo-28-oic acid | Terpenoids | 100040 | 124750 | 117720 | 52623 | 52014 | 54663 | 49263 | 46430 | 66521 | 56284 | 53348 | 51536 | B |
| 144 | pmp000545 | 5 | C_16_H_18_O_9_ | 4-Caffeoylquinic acid | Phenolic acids | 57976 | 56174 | 58587 | 14590 | 15171 | 17173 | 12739 | 19128 | 11769 | 7892 | 14811 | 17567 | A |
| 145 | pme0256 | 5 | C_5_H_4_N_4_O_2_ | Xanthine | Nucleotides and derivatives |  | 1007000 | 917230 | 78484 | 67432 | 78049 | 58574 | 76147 | 111410 | 63285 | 88379 | 73119 | B |
| 146 | Qingke_Rfmb087-1-1 | 6 | C_18_H_32_O_4_ | 13-Oxo-9-hydroxy-10-octadecenoic acid | Lipids | 523770 | 552180 | 598610 | 337280 | 321780 | 325740 | 205820 | 177130 | 241760 | 207520 | 274970 | 266220 | B |
| 147 | mws0467 | 6 | C_9_H_10_O_3_ | 3-(4-Hydroxyphenyl)-propionic acid | Phenolic acids | 321270 | 280680 | 237780 | 34572 | 37593 | 36226 | 9 | 9 | 9 | 18372 | 22202 | 9 | A |
| 148 | mws0628 | 6 | C_7_H_6_O_2_ | 4-Hydroxybenzaldehyde | Phenolic acids | 52119000 | 53053000 | 47752000 | 17563000 | 17468000 | 19456000 | 12612000 | 11941000 | 12257000 | 13478000 | 14349000 | 13023000 | A |
| 149 | mws0863 | 6 | C_5_H_9_NaO_7_P | 2-Deoxyribose 5-phosphate | Nucleotides and derivatives | 271530 | 195680 | 200220 | 125370 | 70800 | 85197 | 64762 | 63558 | 64050 | 65504 | 86687 | 58096 | B |
| 150 | mws1593 | 6 | C_24_H_42_O_21_ | Maltotetraose | Others | 77652 | 73093 | 94670 | 36584 | 27587 | 26466 | 21178 | 21630 | 17826 | 24649 | 21599 | 23215 | B |
| 151 | pmb2799 | 6 | C_18_H_32_O_3_ | 12,13-EODE | Lipids | 216980 | 221770 | 228250 | 87458 | 79246 | 83821 | 56203 | 56513 | 62074 | 56339 | 70444 | 63120 | A |
| 152 | pmb2871 | 6 | C_13_H_16_O_9_ | 2,5-Dihydroxy benzoic acid O-hexside | Phenolic acids | 16488000 | 14524000 | 16483000 | 5944500 | 7986900 | 7535800 | 5002000 | 5073900 | 5881200 | 7122800 | 7822600 | 7574400 | A |
| 153 | pmb3072 | 6 | C_22_H_26_O_12_ | 3-O-p-coumaroyl shikimic acid O-hexoside | Phenolic acids | 9363 | 9295 | 12858 | 5067 | 912 | 5563 | 1384 | 4098 | 2911 | 2381 | 2489 | 3658 | B |
| 154 | pmd0160 | 6 | C_21_H_44_NO_7_P | LysoPE 16:0(2n isomer) | Lipids | 2772700 | 2694000 | 2914000 | 1128900 | 1000800 | 880190 | 571580 | 540770 | 342770 | 435680 | 445860 | 445840 | A |
| 155 | pmn001367 | 6 | C_13_H_16_O_9_ | Protocatechuic acid-4-glucoside | Phenolic acids | 17950000 | 16356000 | 18240000 | 8612000 | 9011600 | 8256400 | 5524600 | 6636300 | 6660800 | 7946900 | 8832700 | 8663000 | A |
| 156 | pmp001249 | 6 | C_20_H_30_NO_9_+ | Caffeoylcholine 6-glucoside | Alkaloids | 17470 | 15520 | 13704 | 8911 | 12589 | 10803 | 7521 | 9400 | 5307 | 9204 | 8092 | 9727 | B |
| 157 | pmp001271 | 6 | C_33_H_58_O_14_ | 2,3-Dihydroxypropyl-9,12-octadecadienoate-hexose-hexose | Lipids | 567730 | 429180 | 447520 | 242080 | 110430 | 129550 | 47382 | 74535 | 78212 | 64467 | 43991 | 68869 | A |
| 158 | Lmgn001670 | 7 | C_7_H_6_O_3_ | Salicylic acid | Phenolic acids | 9 | 9 | 9 | 68406 | 58006 | 78056 | 44364 | 46045 | 48152 | 78512 | 73847 | 77025 | A |
| 159 | Lmnn002886 | 7 | C_23_H_26_O_11_ | 1'-O-β-D-(3,4-dihydroxyphenethyl)-O-caffeoyl-glucoside | Phenolic acids | 2709 | 3991 | 2802 | 69404 | 64101 | 58111 | 48769 | 60484 | 36903 | 75448 | 43973 | 31450 | B |
| 160 | Qingke_Rfmb262-der01-3 | 7 | C_28_H_36_O_13_ | Syringaresinol-Hex | Lignans and Coumarins | 8807 | 9629 | 10105 | 32401 | 37526 | 37369 | 40012 | 32097 | 34084 | 32167 | 34619 | 36292 | B |
| 161 | Qingke_Rfmb262-der14-1 | 7 | C_30_H_38_O_14_ | Syringaresinol-aceGlu | Lignans and Coumarins | 9 | 9 | 9 | 99007 | 84397 | 116850 | 88155 | 83399 | 71673 | 92574 | 90101 | 79760 | B |
| 162 | mws0014 | 7 | C_10_H_10_O_4_ | Ferulic acid | Phenolic acids | 9 | 9 | 9 | 69831 | 100130 | 106020 | 99247 | 95274 | 92760 | 112890 | 89400 | 102950 | A |
| 163 | mws0027 | 7 | C_9_H_10_O_5_ | Syringic acid | Phenolic acids | 9 | 9 | 9 | 177110 | 172840 | 160720 | 130540 | 148470 | 204340 | 173050 | 177230 | 167450 | A |
| 164 | mws0028 | 7 | C_8_H_8_O_4_ | Vanillic acid | Phenolic acids | 199890 | 196410 | 178690 | 1359800 | 1511300 | 1877100 | 1430600 | 1454500 | 1702700 | 1470000 | 1453600 | 1269900 | A |
| 165 | mws0093 | 7 | C_10_H_12_O_3_ | Coniferyl alcohol | Phenolic acids | 4176 | 5464 | 1191 | 20742 | 30060 | 31084 | 40435 | 29301 | 45838 | 29993 | 30270 | 27311 | B |
| 166 | mws0097 | 7 | C_20_H_22_O_6_ | Pinoresinol | Lignans and Coumarins | 9 | 9 | 9 | 31436 | 30922 | 30524 | 22446 | 26924 | 20918 | 30795 | 19996 | 20822 | B |
| 167 | mws0159 | 7 | C_9_H_8_O_3_ | Phenylpyruvic acid | Organic acids | 9 | 9 | 9 | 105130 | 104780 | 111210 | 81279 | 109540 | 88451 | 117530 | 98364 | 96058 | A |
| 168 | mws0213 | 7 | C_5_H1_2_O_5_ | Ribitol | Others | 13206 | 13662 | 10106 | 160130 | 172240 | 165830 | 152570 | 174770 | 155340 | 141260 | 147650 | 131970 | B |
| 169 | mws0236 | 7 | C_2_H_7_NO_3_S | 2-Aminoethanesulfonic acid | Organic acids | 6742 | 4672 | 4212 | 11949 | 18297 | 16481 | 12912 | 10783 | 13199 | 11855 | 11489 | 11833 | B |
| 170 | mws0340 | 7 | C_6_H_8_O_4_ | 2,3-Dimethylsuccinic acid | Amino acids and derivatives | 104290 | 106140 | 103580 | 173820 | 189180 | 212770 | 218750 | 223370 | 217600 | 209250 | 182870 | 183470 | A |
| 171 | mws0437 | 7 | C_5_H_12_O_5_ | D-Arabitol | Others | 9 | 9 | 9 | 128080 | 142000 | 156970 | 135110 | 160420 | 129310 | 125130 | 144090 | 98257 | B |
| 172 | mws0576 | 7 | C_4_H_8_O_3_ | 3-Hydroxybutyrate | Organic acids | 9 | 9 | 9 | 438390 | 391930 | 521140 | 513750 | 518930 | 599110 | 491100 | 469510 | 517350 | B |
| 173 | mws0629 | 7 | C_13_H_16_N_2_O_5_ | Asp-phe | Amino acids and derivatives | 9 | 9 | 9 | 123940 | 113690 | 113760 | 108900 | 96196 | 102130 | 137400 | 129360 | 111680 | B |
| 174 | mws0724 | 7 | C_10_H_13_N_5_O_6_ | 8-Hydroxyguanosine | Nucleotides and derivatives | 21326 | 22397 | 21158 | 91407 | 96633 | 116310 | 58981 | 94127 | 84664 | 71939 | 86615 | 65402 | B |
| 175 | mws0736 | 7 | C_8_H_16_N_2_O_3_ | N-Glycyl-L-leucine | Amino acids and derivatives | 89463 | 129310 | 110930 | 2166800 | 1860200 | 1810800 | 1835600 | 1709900 | 2535000 | 2017800 | 2313300 | 2212500 | A |
| 176 | mws0890 | 7 | C_3_H_7_NO_3_ | Serine | Amino acids and derivatives | 7080 | 8163 | 11102 | 28129 | 31632 | 23726 | 26977 | 27034 | 30134 | 32393 | 29012 | 35648 | B |
| 177 | mws0921 | 7 | C_9_H_10_O_2_ | p-Coumaryl alcohol | Phenolic acids | 600700 | 561660 | 496970 | 2620200 | 2673300 | 2124400 | 2712000 | 2429200 | 2260700 | 1076000 | 1127600 | 1008700 | A |
| 178 | mws1015 | 7 | C_15_H_16_O_9_ | Esculin(6,7-DihydroxyCoumarin-6-glucoside) | Lignans and Coumarins | 856 | 1033 | 746 | 47243 | 42822 | 45297 | 39151 | 41488 | 36187 | 35563 | 34753 | 32053 | B |
| 179 | mws1038 | 7 | C_11_H_22_N_2_O_4_S | (R)-Pantetheine | Others | 9 | 9 | 5866 | 241760 | 260390 | 202310 | 187420 | 221990 | 186970 | 175530 | 139510 | 113910 | A |
| 180 | mws1297 | 7 | C_13_H_18_O_6_ | Benzyl β-D-Glucopyranoside | Phenolic acids | 9 | 9 | 9 | 126430 | 106570 | 137210 | 58523 | 80193 | 80008 | 25828 | 52656 | 57372 | B |
| 181 | mws1354 | 7 | C_10_H_10_O_4_ | Trans-ferulic acid | Phenolic acids | 9 | 9 | 9 | 99542 | 84307 | 107570 | 77424 | 68681 | 132050 | 116110 | 105580 | 79995 | A |
| 182 | mws1562 | 7 | C_15_H_22_O_10_ | Catalpol | Others | 9 | 9 | 9 | 43805 | 48839 | 56765 | 37111 | 52770 | 53258 | 50993 | 48573 | 50456 | B |
| 183 | mws2212 | 7 | C_9_H_8_O_4_ | Caffeic acid | Phenolic acids | 9 | 9 | 9 | 109310 | 110870 | 164740 | 102890 | 90461 | 100820 | 105350 | 106640 | 111050 | B |
| 184 | mws2608 | 7 | C_8_H_15_NO_6_ | N-Acetyl-D-galactosamine | Others | 3114 | 5760 | 5878 | 13317 | 13765 | 8185 | 8943 | 17182 | 8621 | 11170 | 14209 | 11711 | B |
| 185 | mws2625 | 7 | C_20_H_22_O_6_ | Matairesinol | Lignans and Coumarins | 9 | 9 | 9 | 11315 | 16187 | 15979 | 12625 | 11243 | 14434 | 14666 | 17072 | 14720 | B |
| 186 | mws5039 | 7 | C_15_H_16_O_9_ | Esculin Hydrate | Lignans and Coumarins | 1192 | 898 | 488 | 19961 | 22604 | 23301 | 14271 | 14335 | 17189 | 12224 | 17852 | 15481 | B |
| 187 | mws5042 | 7 | C_11_H_14_N_2_O_3_ | Glycylphenylalanine | Amino acids and derivatives | 148570 | 152940 | 163440 | 6443000 | 6228200 | 6571400 | 5478600 | 4914100 | 8178500 | 6424400 | 7486400 | 7016900 | A |
| 188 | pmb0890 | 7 | C_21_H_38_O_4_ | MAG(18:2) | Lipids | 557040 | 536480 | 512960 | 1157400 | 1127800 | 1294200 | 1001000 | 837360 | 1217900 | 928750 | 1110800 | 1218900 | B |
| 189 | pmb1605 | 7 | C_21_H_36_O_4_ | MAG(18:3)isomer3 | Lipids | 9 | 9 | 9 | 274340 | 321380 | 270700 | 122430 | 169070 | 162370 | 153890 | 206400 | 172460 | A |
| 190 | pmb1656 | 7 | C_21_H_36_O_4_ | MAG(18:3)isomer4 | Lipids | 16392 | 18125 | 17273 | 43146 | 38254 | 43527 | 32846 | 29031 | 38797 | 32130 | 33521 | 39794 | B |
| 191 | pmb2221 | 7 | C_18_H_39_NO_3_ | 4-Hydroxysphinganine | Lipids | 3247 | 2239 | 1639 | 11111 | 14207 | 14671 | 10717 | 9841 | 12966 | 18620 | 19522 | 14342 | A |
| 192 | pmb3074 | 7 | C_16_H_18_O_8_ | 3-O-p-Coumaroyl quinic acid | Phenolic acids | 77938 | 102230 | 77689 | 181150 | 212440 | 194550 | 175220 | 176440 | 171490 | 162840 | 203910 | 179880 | B |
| 193 | pmb3081 | 7 | C_6_H_11_PO_11_ | Glucarate O-Phosphoric acid | Others | 8198900 | 8594800 | 8485600 | 33205000 | 27718000 | 28336000 | 25753000 | 25138000 | 24023000 | 27028000 | 27177000 | 26699000 | B |
| 194 | pme0124 | 7 | C_7_H_12_N_2_O_3_ | Glycyl-L-proline | Amino acids and derivatives | 36929 | 45767 | 46280 | 372660 | 342710 | 303660 | 345110 | 354450 | 386590 | 265600 | 326850 | 359390 | B |
| 195 | pme0128 | 7 | C_6_H_12_N_2_O_3_ | AlanylAlanine | Amino acids and derivatives | 2944 | 5033 | 3244 | 16295 | 13620 | 14985 | 12828 | 13538 | 12878 | 17422 | 19068 | 20128 | B |
| 196 | pme0137 | 7 | C_7_H_12_N_2_O_4_ | N-α-Acetyl-L-glutamine | Amino acids and derivatives | 9 | 9 | 9 | 89897 | 98100 | 122610 | 112470 | 77179 | 105830 | 125980 | 112550 | 101280 | B |
| 197 | pme0257 | 7 | C_4_H_4_N_2_O_2_ | Uracil | Nucleotides and derivatives | 9 | 9 | 9 | 22335000 | 22233000 | 26344000 | 11353000 | 16199000 | 10490000 | 8303900 | 11275000 | 10809000 | A |
| 198 | pme0264 | 7 | C_10_H_14_N_2_O_5_ | Thymidine | Nucleotides and derivatives | 320130 | 354920 | 317480 | 1367100 | 1461700 | 1343300 | 1585700 | 1492400 | 1643600 | 1349600 | 1470300 | 1430200 | B |
| 199 | pme1086 | 7 | C_10_H_17_N_3_O_6_S | Glutathione reduced form | Amino acids and derivatives | 3644 | 2325 | 1230 | 51802 | 59643 | 38913 | 32215 | 38777 | 21811 | 23404 | 9772 | 11941 | B |
| 200 | pme1184 | 7 | C_10_H_13_N_5_O_4_ | Deoxyguanosine | Nucleotides and derivatives | 467170 | 488110 | 398170 | 4008500 | 4525500 | 4917200 | 5016500 | 4509800 | 5780500 | 4470400 | 4900100 | 5283000 | B |
| 201 | pme1228 | 7 | C_11_H_12_N_2_O_3_ | 5-Hydroxy-L-tryptophan | Amino acids and derivatives | 11359 | 19992 | 19784 | 45683 | 56801 | 52049 | 50393 | 57697 | 63413 | 35526 | 64129 | 62552 | B |
| 202 | pme1373 | 7 | C_9_H_14_N_3_O_7_P | 2'-Deoxycytidine-5'-monophosphate | Nucleotides and derivatives | 5554 | 4849 | 5136 | 13400 | 15274 | 13122 | 18878 | 23835 | 24259 | 12683 | 15222 | 11258 | B |
| 203 | pme2482 | 7 | C_7_H_6_O_3_ | Protocatechuic aldehyde | Flavonoids | 41671 | 42106 | 53830 | 78051 | 83094 | 85318 | 88225 | 61678 | 48200 | 104850 | 103050 | 70781 | B |
| 204 | pme2602 | 7 | C_3_H_8_NO_6_P | O-Phospho-L-serine | Amino acids and derivatives | 5465 | 3649 | 9541 | 27967 | 41178 | 29167 | 26540 | 31142 | 34708 | 19933 | 26682 | 38423 | B |
| 205 | pme2954 | 7 | C_15_H_10_O_7_ | Quercetin | Flavonoids | 13936 | 11180 | 11521 | 35275 | 32402 | 39263 | 45728 | 44170 | 41104 | 46176 | 39097 | 34154 | A |
| 206 | pme3009 | 7 | C_6_H_6_O_6_ | Trans-Citridic acid | Organic acids | 24648 | 23440 | 27774 | 51589 | 48897 | 47195 | 44760 | 42934 | 52617 | 57257 | 52003 | 56372 | B |
| 207 | pmn001502 | 7 | C_21_H_24_O_7_ | Medioresinol | Lignans and Coumarins | 687 | 302 | 892 | 3320 | 3312 | 5379 | 2203 | 2999 | 4265 | 3140 | 2759 | 3936 | B |
| 208 | pmn001518 | 7 | C_13_H_16_O_10_ | 1-O-Galloyl-β-D-glucose | Phenolic acids | 9 | 9 | 9 | 6575 | 6696 | 6726 | 5252 | 6380 | 4922 | 4429 | 9 | 9 | B |
| 209 | pmn001627 | 7 | C_13_H_16_O_10_ | Glucogallin | Phenolic acids | 9 | 9 | 9 | 74251 | 44361 | 61566 | 56198 | 59287 | 50660 | 63822 | 76278 | 62970 | B |
| 210 | pmn001682 | 7 | C_9_H_8_O_4_ | Sorbic acid | Phenolic acids | 9 | 9 | 9 | 73002 | 77685 | 83000 | 76919 | 70537 | 54204 | 66377 | 69922 | 63654 | B |
| 211 | pmn001712 | 7 | C_22_H_34_O_12_ | 3-Hydroxy-4-isopropylbenzylalcohol 3-glucoside-glucoside | Phenolic acids | 9 | 9 | 9 | 8515 | 7886 | 7532 | 7676 | 7172 | 11024 | 6853 | 7477 | 11938 | B |
| 212 | pmp001045 | 7 | C_15_H_22_O_8_ | Bartsioside | Others | 3790 | 2039 | 6558 | 15426 | 17527 | 21387 | 20939 | 21396 | 24392 | 16885 | 21275 | 19443 | B |
| 213 | mws0178 | 8 | C_16_H_18_O_9_ | Chlorogenic acid | Phenolic acids | 48283 | 57286 | 47767 | 26931 | 11231 | 12716 | 9236 | 10104 | 13261 | 11844 | 6654 | 6212 | A |
| 214 | mws0192 | 8 | C_4_H_6_O_4_ | Succinic acid | Organic acids | 99506000 | 109780000 | 98253000 | 47913000 | 47708000 | 46529000 | 43769000 | 39001000 | 43081000 | 32762000 | 36827000 | 39612000 | A |
| 215 | mws0470 | 8 | C_4_H_6_O_4_ | Methylmalonic acid | Organic acids | 97877000 | 109890000 | 96117000 | 47501000 | 47472000 | 46925000 | 43994000 | 38515000 | 43574000 | 32087000 | 36661000 | 39633000 | B |
| 216 | mws0574 | 8 | C_4_H_8_O_3_ | α-Hydroxyisobutyric acid | Organic acids | 9 | 535790 | 9 | 72023 | 42741 | 45838 | 31501 | 34434 | 65529 | 23389 | 24175 | 25557 | B |
| 217 | mws0712 | 8 | C_5_H_9_NO_3_ | N-Propionylglycine | Amino acids and derivatives | 467010 | 508980 | 453860 | 216900 | 183540 | 195720 | 144470 | 131930 | 132220 | 84375 | 106950 | 69018 | A |
| 218 | mws0805 | 8 | C_11_H_19_NO_9_ | N-Acetylneuraminic acid | Amino acids and derivatives | 63484 | 45246 | 44399 | 24042 | 27126 | 31255 | 34809 | 29280 | 20861 | 21152 | 20747 | 22541 | B |
| 219 | mws1155 | 8 | C_6_H_14_O_6_ | Mannitol | Others | 62606 | 61114 | 63753 | 34832 | 34831 | 22859 | 21970 | 27143 | 31506 | 14734 | 26477 | 24146 | B |
| 220 | mws1333 | 8 | C_12_H_22_O_11_ | Melibiose | Others | 1347800 | 1061500 | 1001600 | 481370 | 473930 | 471880 | 359030 | 395730 | 360030 | 371880 | 351050 | 329820 | A |
| 221 | mws4175 | 8 | C_6_H_8_O_6_ | D-Glucurono-6,3-lactone | Others | 94175 | 67126 | 75051 | 41191 | 33949 | 40522 | 35855 | 35434 | 35295 | 31343 | 33979 | 33402 | B |
| 222 | mws5038 | 8 | C_12_H_22_O_11_ | Isomaltulose | Others | 3410600 | 2926300 | 3094300 | 1312800 | 1180400 | 1281400 | 1084800 | 1144000 | 978060 | 983020 | 974720 | 953670 | A |
| 223 | pmb0164 | 8 | C_21_H_38_O_4_ | MAG(18:2)isomer1 | Lipids | 657230 | 676260 | 692570 | 300490 | 274810 | 261180 | 135780 | 163370 | 134060 | 103660 | 108110 | 93597 | A |
| 224 | pmb0423 | 8 | C_10_H_10_O_4_ | Hydroxy-methoxycinnamate | Phenolic acids | 24119 | 27613 | 18460 | 10031 | 10481 | 14509 | 7481 | 21924 | 15174 | 11906 | 9562 | 8953 | B |
| 225 | pmb0864 | 8 | C_19_H_40_NO_7_P | LysoPE 14:0 | Lipids | 25189 | 25586 | 23793 | 12372 | 11026 | 10771 | 8831 | 7768 | 11078 | 5864 | 10572 | 8395 | A |
| 226 | pmb1562 | 8 | C_21_H_34_O_4_ | MAG(18:4)isomer3 | Lipids | 192510 | 229490 | 184010 | 77140 | 79608 | 58349 | 64109 | 42398 | 57515 | 67504 | 9 | 9 | A |
| 227 | pmb2319 | 8 | C_23_H_48_NO_7_P | LysoPC 15:0 | Lipids | 597840 | 574660 | 571280 | 346300 | 328660 | 288470 | 303980 | 236660 | 446010 | 240430 | 277310 | 227450 | A |
| 228 | pmd0132 | 8 | C_24_H_50_NO_7_P | LysoPC 16:0(2n isomer) | Lipids | 10330000 | 9983400 | 9744100 | 4696300 | 4559900 | 3798000 | 3332800 | 3188400 | 3878500 | 2368500 | 3265900 | 2972300 | A |
| 229 | pmd0136 | 8 | C_26_H_54_NO_7_P | LysoPC 18:0 | Lipids | 318670 | 331180 | 314650 | 179210 | 162100 | 144000 | 108380 | 109190 | 157960 | 99717 | 103110 | 102660 | A |
| 230 | pme2237 | 8 | C_6_H_14_O_6_ | Dulcitol | Others | 240730 | 212880 | 247770 | 155250 | 123170 | 110630 | 71844 | 95747 | 151040 | 67166 | 88537 | 99141 | B |
| 231 | pme2743 | 8 | C_10_H_11_NO_3_ | N-Phenylacetylglycine | Amino acids and derivatives | 22053 | 19173 | 21769 | 9156 | 6734 | 9077 | 4159 | 5518 | 8209 | 3023 | 6158 | 1831 | B |
| 232 | pme3154 | 8 | C_6_H_12_O_4_ | (Rs)-Mevalonic acid | Organic acids | 8188200 | 7386200 | 7918100 | 2297500 | 2099800 | 2239700 | 1542000 | 1636100 | 1914100 | 1106700 | 1445500 | 1234900 | B |
| 233 | pme3968 | 8 | C_6_H_7_N_5_O | 7-Methylguanine | Nucleotides and derivatives | 67190 | 66420 | 68536 | 31633 | 38305 | 32164 | 29352 | 30212 | 24595 | 23854 | 25714 | 25945 | B |
| 234 | pmn001419 | 8 | C_15_H_18_O_8_ | 1-O-[(E)-p-Cumaroyl]-β-D-glucopyranose | Phenolic acids | 1777400 | 1823700 | 1712700 | 602710 | 656350 | 611070 | 570410 | 557000 | 495230 | 469690 | 470070 | 471770 | A |
| 235 | mws0177 | 9 | C_5_H_4_O_3_ | 2-Furanoic acid | Organic acids | 380940 | 393120 | 353040 | 203010 | 160940 | 172990 | 189570 | 183330 | 221400 | 267090 | 361330 | 347530 | B |
| 236 | pme0444 | 9 | C_23_H_25_O_12_+ | Malvidin 3-O-glucoside (Oenin) | Flavonoids | 14921 | 16896 | 11960 | 9987 | 5007 | 4942 | 10777 | 9929 | 4949 | 10453 | 13838 | 11535 | B |
| 237 | pme2853 | 9 | C_8_H_15_NO_3_ | Hexanoyl glycine | Amino acids and derivatives | 35856 | 24356 | 31797 | 10909 | 16157 | 15168 | 15464 | 11725 | 12963 | 14646 | 26490 | 16653 | B |
| 238 | pmn001705 | 9 | C_30_H_48_O_4_ | 3,24-Dihydroxy-17,21-semiacetal-12(13)oleanolic fruit | Terpenoids | 94337 | 105350 | 91011 | 36474 | 47477 | 27126 | 16183 | 40336 | 35683 | 43052 | 84670 | 39793 | B |
| 239 | pmn001710 | 9 | C_24_H_26_O_13_ | Rosmarinyl Glucoside | Phenolic acids | 234940 | 194850 | 165350 | 81669 | 92429 | 78438 | 113030 | 108820 | 124090 | 149550 | 202150 | 200220 | A |

A^a^: The second-level mass spectrometry and RT of the substance are consistent with the database; B^b^: The parameters of Q1, Q3, RT, DP and CE of the substance are consistent with the database.

**Table S4** 193 sulfur-fumigation markers in TR.

| Code | Index | Formula | Compounds | Class I | Class II | Level | VIP | Log2FC | Type |
| --- | --- | --- | --- | --- | --- | --- | --- | --- | --- |
| 1 | mws0159 | C_9_H_8_O_3_ | Phenylpyruvic acid | Organic acids | Organic acids | A^a^ | 1.17 | 13.54 | up |
| 2 | mws0177 | C_5_H_4_O_3_ | 2-Furanoic acid | Organic acids | Organic acids | B^b^ | 1.15 | -1.07 | down |
| 3 | mws0192 | C_4_H_6_O_4_ | Succinic acid | Organic acids | Organic acids | A | 1.16 | -1.11 | down |
| 4 | mws0236 | C_2_H_7_NO_3_S | 2-Aminoethanesulfonic acid | Organic acids | Organic acids | B | 1.11 | 1.58 | up |
| 5 | mws0237 | C_9_H_16_O_4_ | Anchoic Acid | Organic acids | Organic acids | A | 1.17 | -1.44 | down |
| 6 | mws0376 | C_4_H_4_O_4_ | Fumaric acid | Organic acids | Organic acids | A | 1.17 | -4.09 | down |
| 7 | mws0470 | C_4_H_6_O_4_ | Methylmalonic acid | Organic acids | Organic acids | B | 1.16 | -1.10 | down |
| 8 | mws0474 | C_12_H_22_O_4_ | Dodecanedioic aicd | Organic acids | Organic acids | A | 1.15 | -2.19 | down |
| 9 | mws0489 | C_8_H_6_O_3_ | Benzoylformic acid | Organic acids | Organic acids | B | 1.16 | -2.76 | down |
| 10 | mws0567 | C_5_H_11_N_3_O_2_ | 4-Guanidinobutyric acid | Organic acids | Organic acids | B | 1.14 | -1.02 | down |
| 11 | mws0576 | C_4_H_8_O_3_ | 3-Hydroxybutyrate | Organic acids | Organic acids | B | 1.17 | 15.61 | up |
| 12 | mws0639 | C_7_H_6_O_4_ | 2,3-Dihydroxybenzoic Acid | Organic acids | Organic acids | B | 1.17 | 20.95 | up |
| 13 | mws0972 | C_6_H_12_O_3_ | 5-Hydroxyhexanoic acid | Organic acids | Organic acids | B | 1.16 | -1.67 | down |
| 14 | pme0295 | C_6_H_11_NO_3_ | 4-Acetamidobutyric acid | Organic acids | Organic acids | B | 1.17 | -2.91 | down |
| 15 | pme2049 | C_4_H_8_O_3_ | 2-Hydroxybutanoic acid | Organic acids | Organic acids | B | 1.15 | -1.15 | down |
| 16 | pme3154 | C_6_H_12_O_4_ | (Rs)-Mevalonic acid | Organic acids | Organic acids | B | 1.17 | -1.82 | down |
| 17 | pme3207 | C_6_H_6_O_4_ | Trans,trans-Muconic acid | Organic acids | Organic acids | B | 1.16 | -1.12 | down |
| 18 | Qingke_Rfmb089-2-3 | C_18_H_34_O_4_ | 9,10-Dihydroxy-12-octadecenoic acid | Lipids | Free fatty acids | A | 1.17 | -2.82 | down |
| 19 | Qingke_Rfmb090-1-3 | C_18_H_32_O_3_ | 13-Hydroxy-9,11-octadecadienoic acid | Lipids | Free fatty acids | A | 1.17 | -1.60 | down |
| 20 | Qingke_Rfmb091-1-1 | C_18_H_32_O_3_ | 9-Hydroxy-10,12-octadecadienoic acid | Lipids | Free fatty acids | A | 1.16 | -1.56 | down |
| 21 | mws5045 | C_12_H_24_O_3_ | 12-Hydroxydodecanoic acid | Lipids | Free fatty acids | A | 1.16 | -2.13 | down |
| 22 | pmb0889 | C_18_H_30_O_2_ | Punicic acid | Lipids | Free fatty acids | A | 1.17 | -1.56 | down |
| 23 | pmb2778 | C_18_H_32_O_3_ | 9,10-EODE | Lipids | Free fatty acids | A | 1.17 | -1.75 | down |
| 24 | pmb2784 | C_18_H_30_O_3_ | 13-HOTrE | Lipids | Free fatty acids | A | 1.16 | -2.17 | down |
| 25 | pmb2786 | C_18_H_30_O_3_ | 9-HOTrE | Lipids | Free fatty acids | A | 1.16 | -1.36 | down |
| 26 | pmb2787 | C_18_H_30_O_3_ | 9-KODE | Lipids | Free fatty acids | A | 1.15 | -1.42 | down |
| 27 | pmb2792 | C_18_H_30_O_3_ | 13-HOTrE(r) | Lipids | Free fatty acids | A | 1.10 | 1.80 | up |
| 28 | pmb2799 | C_18_H_32_O_3_ | 12,13-EODE | Lipids | Free fatty acids | A | 1.17 | -1.41 | down |
| 29 | pmn001688 | C_18_H_32_O_3_ | 9S-Hyroxy-10E,12E-octadecadienoic acid | Lipids | Free fatty acids | A | 1.17 | -1.52 | down |
| 30 | pmn001689 | C_18_H_32_O_4_ | 9-Hydroxy-12-oxo-10-octadecenoic acid | Lipids | Free fatty acids | A | 1.17 | -2.56 | down |
| 31 | pmn001691 | C_18_H_32_O_5_ | 9,12,13-Trihyroxy-10,15-octadecadienoic acid | Lipids | Free fatty acids | A | 1.17 | -2.77 | down |
| 32 | pmn001694 | C_18_H_34_O_5_ | 9,10,13-Trihyroxy-11-octadecadienoic acid | Lipids | Free fatty acids | A | 1.17 | -2.03 | down |
| 33 | pmp001271 | C_33_H_58_O_14_ | 2,3-Dihydroxypropyl-9,12-octadecadienoate-hexose-hexose | Lipids | Free fatty acids | A | 1.07 | -1.58 | down |
| 34 | mws0005 | C_10_H_12_N_2_ | Tryptamine | Alkaloids | Plumerane | B | 1.17 | -13.86 | down |
| 35 | mws1015 | C_15_H_16_O_9_ | Esculin(6,7-DihydroxyCoumarin-6-glucoside) | Lignans and Coumarins | Coumarins | B | 1.17 | 5.68 | up |
| 36 | mws5039 | C_15_H_16_O_9_ | Esculin Hydrate | Lignans and Coumarins | Coumarins | B | 1.15 | 4.68 | up |
| 37 | pme2987 | C_9_H_8_O_2_ | 3,4-Dihydrocoumarin | Lignans and Coumarins | Coumarins | B | 1.17 | -4.25 | down |
| 38 | pmn001492 | C_11_H_8_O_3_ | Ayapin | Lignans and Coumarins | Coumarins | A | 1.17 | -1.57 | down |
| 39 | Hmfn00531 | C_6_H_8_O_6_ | L-Ascorbic acid | Others | Vitamin | B | 1.17 | -12.77 | down |
| 40 | pme2266 | C_10_H_16_N_2_O_3_S | Biotin | Others | Vitamin | B | 1.07 | -1.75 | down |
| 41 | mws0213 | C_5_H_12_O_5_ | Ribitol | Others | Saccharides and Alcohols | B | 1.17 | 3.75 | up |
| 42 | mws0264 | C_12_H_22_O_11_ | D-(+)-TrehaloseAnhydrous | Others | Saccharides and Alcohols | A | 1.14 | -1.60 | down |
| 43 | mws0437 | C_5_H_12_O_5_ | D-Arabitol | Others | Saccharides and Alcohols | B | 1.17 | 13.95 | up |
| 44 | mws1080 | C_12_H_22_O_11_ | Galactinol | Others | Saccharides and Alcohols | A | 1.16 | -1.94 | down |
| 45 | mws1155 | C_6_H_14_O_6_ | Mannitol | Others | Saccharides and Alcohols | B | 1.09 | -1.02 | down |
| 46 | mws1333 | C_12_H_22_O_11_ | Melibiose | Others | Saccharides and Alcohols | A | 1.14 | -1.26 | down |
| 47 | mws1593 | C_24_H_42_O_21_ | Maltotetraose | Others | Saccharides and Alcohols | B | 1.13 | -1.44 | down |
| 48 | mws4163 | C_24_H_42_O_21_ | 1,1-Kestotetraose | Others | Saccharides and Alcohols | B | 1.15 | -1.97 | down |
| 49 | mws5038 | C_12_H_22_O_11_ | Isomaltulose | Others | Saccharides and Alcohols | A | 1.16 | -1.32 | down |
| 50 | mws5040 | C_12_H_21_O_11_Na | Turanose | Others | Saccharides and Alcohols | B | 1.17 | -13.78 | down |
| 51 | pmb3081 | C_6_H_11_PO_11_ | Glucarate O-Phosphoric acid | Others | Saccharides and Alcohols | B | 1.16 | 1.82 | up |
| 52 | pme0500 | C_18_H_32_O_16_ | D-(+)-Melezitose | Others | Saccharides and Alcohols | B | 1.15 | -1.43 | down |
| 53 | pme0519 | C_12_H_22_O_11_ | D-(+)-Sucrose | Others | Saccharides and Alcohols | A | 1.16 | -2.33 | down |
| 54 | mws0677 | C_12_H_14_N_2_O_2_ | N-Acetyl-5-hydroxytryptamine | Alkaloids | Alkaloids | B | 1.17 | -11.56 | down |
| 55 | pme1137 | C_6_H_5_NO_3_ | 6-Hydroxynicotinic acid | Alkaloids | Alkaloids | B | 1.12 | -1.61 | down |
| 56 | pmp000628 | C_15_H_20_N_2_O_2_ | 9α-Hydroxysophoramine | Alkaloids | Alkaloids | B | 1.17 | 14.31 | up |
| 57 | Xigua_mab0299 | C_36_H_54_O_12_ | Dihydroisocucurbitacin I-Hex | Terpenoids | Triterpene Saponin | A | 1.08 | -1.70 | down |
| 58 | Xigua_mab0310 | C_36_H_54_O_12_ | Cucurbitacin D O-glucoside | Terpenoids | Triterpene Saponin | A | 1.08 | -1.70 | down |
| 59 | Xigua_mab0406 | C_38_H_56_O_13_ | Cucurbitacin D-aceGlu | Terpenoids | Triterpene Saponin | A | 1.15 | -2.63 | down |
| 60 | Xigua_mab0543 | C_38_H_56_O_13_ | 23,24-Dihydro cucurbitacin E O-glucoside | Terpenoids | Triterpene Saponin | A | 1.13 | -2.10 | down |
| 61 | pmp000898 | C_36_H_54_O_11_ | Deacetoxycucurbitacin B 3-O-glucoside | Terpenoids | Triterpene Saponin | B | 1.16 | -3.09 | down |
| 62 | pmp000899 | C_36_H_56_O_12_ | Cucurbitacin F O-glucoside | Terpenoids | Triterpene Saponin | B | 1.13 | -4.32 | down |
| 63 | pmp000901 | C_38_H_56_O_13_ | Isocucurbitacin B 2-O-glucoside | Terpenoids | Triterpene Saponin | B | 1.15 | -1.89 | down |
| 64 | Xigua_cub060 | C_32_H_46_O_8_ | 23,24-Dihydro cucurbitacin E | Terpenoids | Triterpene | B | 1.15 | -2.68 | down |
| 65 | pmn001429 | C_32_H_46_O_9_ | Cucurbitacin A | Terpenoids | Triterpene | B | 1.17 | -3.26 | down |
| 66 | pmn001433 | C_30_H_46_O_7_ | Cucurbitacin F | Terpenoids | Triterpene | B | 1.17 | -2.22 | down |
| 67 | pmn001705 | C_30_H_48_O_4_ | 3,24-Dihydroxy-17,21-semiacetal-12(13)oleanolic fruit | Terpenoids | Triterpene | B | 1.11 | -1.39 | down |
| 68 | pmp000270 | C_30_H_44_O_6_ | Lup-12-en-15α,19β-diol-3,11-dioxo-28-oic acid | Terpenoids | Triterpene | B | 1.15 | -1.10 | down |
| 69 | pmp000896 | C_32_H_46_O_8_ | Isocucurbitacin B | Terpenoids | Triterpene | B | 1.17 | -3.66 | down |
| 70 | pmb0864 | C_19_H_40_NO_7_P | LysoPE 14:0 | Lipids | LPE | A | 1.16 | -1.13 | down |
| 71 | pmd0160 | C_21_H_44_NO_7_P | LysoPE 16:0(2n isomer) | Lipids | LPE | A | 1.16 | -1.48 | down |
| 72 | pmb0863 | C_24_H_46_NO_7_P | LysoPC 16:2(2n isomer) | Lipids | LPC | A | 1.16 | 3.73 | up |
| 73 | pmb0865 | C_26_H_48_NO_7_P | LysoPC 18:3(2n isomer) | Lipids | LPC | A | 1.17 | 3.35 | up |
| 74 | pmd0130 | C_22_H_46_NO_7_P | LysoPC 14:0(2n isomer) | Lipids | LPC | A | 1.03 | -1.42 | down |
| 75 | pmd0132 | C_24_H_50_NO_7_P | LysoPC 16:0(2n isomer) | Lipids | LPC | A | 1.15 | -1.20 | down |
| 76 | pmp001281 | C_26_H_52_NO_7_P | LysoPC(18:1) | Lipids | LPC | A | 1.16 | 2.48 | up |
| 77 | pmb2221 | C_18_H_39_NO_3_ | 4-Hydroxysphinganine | Lipids | Sphingolipids | A | 1.14 | 2.49 | up |
| 78 | mws0601 | C_5_H_5_NO_2_ | Pyrrole-2-carboxylic acid | Others | Others | B | 1.17 | -2.32 | down |
| 79 | mws1038 | C_11_H_22_N_2_O_4_S | (R)-Pantetheine | Others | Others | A | 1.03 | 6.90 | up |
| 80 | mws1562 | C_15_H_22_O_10_ | Catalpol | Others | Others | B | 1.17 | 12.43 | up |
| 81 | mws4175 | C_6_H_8_O_6_ | D-Glucurono-6,3-lactone | Others | Others | B | 1.11 | -1.03 | down |
| 82 | pmp001045 | C_15_H_22_O_8_ | Bartsioside | Others | Others | B | 1.07 | 2.13 | up |
| 83 | Qingke_Rfmb262-der01-3 | C_28_H_36_O_13_ | Syringaresinol-Hex | Lignans and Coumarins | Lignans | B | 1.16 | 1.91 | up |
| 84 | Qingke_Rfmb262-der14-1 | C_30_H_38_O_14_ | Syringaresinol-aceGlu | Lignans and Coumarins | Lignans | B | 1.17 | 13.44 | up |
| 85 | mws0097 | C_20_H_22_O_6_ | Pinoresinol | Lignans and Coumarins | Lignans | B | 1.17 | 11.75 | up |
| 86 | mws2625 | C_20_H_22_O_6_ | Matairesinol | Lignans and Coumarins | Lignans | B | 1.17 | 10.65 | up |
| 87 | pmn001368 | C_32_H_44_O_17_ | Olivin Diglucoside | Lignans and Coumarins | Lignans | B | 1.16 | -1.74 | down |
| 88 | pmn001370 | C_32_H_42_O_16_ | Pinoresinol diglucoside | Lignans and Coumarins | Lignans | B | 1.15 | -2.11 | down |
| 89 | pmn001502 | C_21_H_24_O_7_ | Medioresinol | Lignans and Coumarins | Lignans | B | 1.09 | 2.67 | up |
| 90 | pmp001251 | C_26_H_51_NO_7_P+ | PC(18:2)isomer | Lipids | PC | A | 1.16 | 3.43 | up |
| 91 | mws0024 | C_7_H_6_O_5_ | Gallic acid | Flavonoids | Flavanols | B | 1.17 | 11.32 | up |
| 92 | mws0183 | C_7_H_6_O_4_ | Protocatechuic acid | Flavonoids | Flavanols | A | 1.17 | 20.96 | up |
| 93 | pmb3894 | C_17_H_14_O_7_ | Di-O-methylquercetin | Flavonoids | Flavonols | B | 1.17 | -2.05 | down |
| 94 | pme2954 | C_15_H_10_O_7_ | Quercetin | Flavonoids | Flavonols | A | 1.15 | 1.55 | up |
| 95 | mws0072 | C_21_H_20_O_10_ | Apigenin 5-O-glucoside | Flavonoids | Flavonoid | B | 1.15 | -1.86 | down |
| 96 | pmb3041 | C_23_H_22_O_14_ | Tricin O-saccharic acid | Flavonoids | Flavonoid | B | 1.17 | 13.30 | up |
| 97 | pmn001668 | C_22_H_24_O_8_ | Apigenin-3-O-α-L-rhamnoside | Flavonoids | Flavonoid | B | 1.12 | -1.74 | down |
| 98 | pme0444 | C_23_H_25_O_12_+ | Malvidin 3-O-glucoside (Oenin) | Flavonoids | Anthocyanins | B | 1.00 | -1.13 | down |
| 99 | mws0255 | C_4_H_5_N_3_O | Cytosine | Nucleotides and derivatives | Nucleotides and derivatives | A | 1.15 | 1.46 | up |
| 100 | mws0572 | C_5_H_7_N_3_O | 5-Methylcytosine | Nucleotides and derivatives | Nucleotides and derivatives | B | 1.17 | 12.16 | up |
| 101 | mws0609 | C_10_H_12_N_5_O_7_P | Guanosine 3',5'-cyclic monophosphate | Nucleotides and derivatives | Nucleotides and derivatives | B | 1.16 | 3.16 | up |
| 102 | mws0724 | C_10_H_13_N_5_O_6_ | 8-Hydroxyguanosine | Nucleotides and derivatives | Nucleotides and derivatives | B | 1.16 | 2.23 | up |
| 103 | mws0863 | C_5_H_9_NaO_7_P | 2-Deoxyribose 5-phosphate | Nucleotides and derivatives | Nucleotides and derivatives | B | 1.07 | -1.25 | down |
| 104 | mws1060 | C_10_H_12_N_4_O_5_ | 9-(β-D-Arabinofuranosyl)hypoxanthine | Nucleotides and derivatives | Nucleotides and derivatives | A | 1.16 | -3.20 | down |
| 105 | pmb0530 | C_21_H_27_N_7_O_14_P_2_ | Nicotinic acid adenine dinucleotide | Nucleotides and derivatives | Nucleotides and derivatives | B | 1.05 | -1.94 | down |
| 106 | pmb0998 | C_10_H_14_N_5_O_8_P | Guanosine 5'-monophosphate | Nucleotides and derivatives | Nucleotides and derivatives | B | 1.03 | 1.83 | up |
| 107 | pmb2922 | C_15_H_24_N_2_O_17_P_2_ | Uridine 5'-diphospho-D-glucose | Nucleotides and derivatives | Nucleotides and derivatives | A | 1.15 | 1.79 | up |
| 108 | pme0256 | C_5_H_4_N_4_O_2_ | Xanthine | Nucleotides and derivatives | Nucleotides and derivatives | B | 1.17 | -15.00 | down |
| 109 | pme0257 | C_4_H_4_N_2_O_2_ | Uracil | Nucleotides and derivatives | Nucleotides and derivatives | A | 1.17 | 21.32 | up |
| 110 | pme0264 | C_10_H_14_N_2_O_5_ | Thymidine | Nucleotides and derivatives | Nucleotides and derivatives | B | 1.17 | 2.07 | up |
| 111 | pme1109 | C_5_H_5_N_5_O | Guanine | Nucleotides and derivatives | Nucleotides and derivatives | A | 1.16 | -2.03 | down |
| 112 | pme1173 | C_5_H_4_N_4_O | Allopurinol | Nucleotides and derivatives | Nucleotides and derivatives | B | 1.15 | -1.03 | down |
| 113 | pme1184 | C_10_H_13_N_5_O_4_ | Deoxyguanosine | Nucleotides and derivatives | Nucleotides and derivatives | B | 1.17 | 3.31 | up |
| 114 | pme1194 | C_9_H_13_N_3_O_4_ | Deoxycytidine | Nucleotides and derivatives | Nucleotides and derivatives | B | 1.17 | 15.47 | up |
| 115 | pme1373 | C_9_H_14_N_3_O_7_P | 2'-Deoxycytidine-5'-monophosphate | Nucleotides and derivatives | Nucleotides and derivatives | B | 1.16 | 1.43 | up |
| 116 | pme3732 | C_9_H_13_N_3_O_5_ | Cytidine | Nucleotides and derivatives | Nucleotides and derivatives | B | 1.16 | 1.61 | up |
| 117 | Hmyn007168 | C_22_H_45_O_9_P | LysoPG(16:0) | Lipids | Glycerol ester | B | 1.14 | 1.06 | up |
| 118 | pmb0164 | C_21_H_38_O_4_ | MAG(18:2)isomer1 | Lipids | Glycerol ester | A | 1.16 | -1.28 | down |
| 119 | pmb0890 | C_21_H_38_O_4_ | MAG(18:2) | Lipids | Glycerol ester | B | 1.16 | 1.16 | up |
| 120 | pmb1562 | C_21_H_34_O_4_ | MAG(18:4)isomer3 | Lipids | Glycerol ester | A | 1.14 | -1.49 | down |
| 121 | pmb1605 | C_21_H_36_O_4_ | MAG(18:3)isomer3 | Lipids | Glycerol ester | A | 1.17 | 14.97 | up |
| 122 | pmb1656 | C_21_H_36_O_4_ | MAG(18:3)isomer4 | Lipids | Glycerol ester | B | 1.16 | 1.27 | up |
| 123 | pmb2325 | C_21_H_36_O_4_ | MAG(18:3)isomer2 | Lipids | Glycerol ester | A | 1.16 | -1.54 | down |
| 124 | pmb2363 | C_21_H_40_O_4_ | MAG(18:1)isomer1 | Lipids | Glycerol ester | A | 1.16 | -1.05 | down |
| 125 | pmn001495 | C_19_H_38_O_4_ | Hexadecanoic acid 2,3-dihydroxypropyl ester | Lipids | Glycerol ester | A | 1.17 | -2.07 | down |
| 126 | Lmgn001670 | C_7_H_6_O_3_ | Salicylic acid | Phenolic acids | Phenolic acids | A | 1.17 | 12.89 | up |
| 127 | Lmhn002926 | C_13_H_12_O_7_ | p-coumaroylmalic acid | Phenolic acids | Phenolic acids | B | 1.17 | 10.94 | up |
| 128 | Lmlp002133 | C_9_H_8_O_3_ | Trans-p-Hydroxycinnamic acid | Phenolic acids | Phenolic acids | B | 1.17 | -2.97 | down |
| 129 | Lmnn002886 | C_23_H_26_O_11_ | 1'-O-β-D-(3,4-dihydroxyphenethyl)-O-caffeoyl-glucoside | Phenolic acids | Phenolic acids | B | 1.16 | 4.33 | up |
| 130 | Lmtn003866 | C_9_H_8_O_2_ | trans-Cinnamic acid | Phenolic acids | Phenolic acids | B | 1.17 | -4.21 | down |
| 131 | Lmtp002625 | C_9_H_8_O_3_ | p-Hydroxycinnamic acid | Phenolic acids | Phenolic acids | B | 1.17 | -2.98 | down |
| 132 | Zmhn001926 | C_13_H_16_O_8_ | Salicylic acid O-glycoside | Phenolic acids | Phenolic acids | A | 1.17 | -2.33 | down |
| 133 | Zmhn002301 | C_15_H_18_O_8_ | p-Coumaric acid-O-glycoside | Phenolic acids | Phenolic acids | A | 1.17 | -1.59 | down |
| 134 | Zmhn002422 | C_16_H_20_O_9_ | Feruloyl glucose | Phenolic acids | Phenolic acids | B | 1.16 | -1.55 | down |
| 135 | Zmxn001997 | C_13_H_16_O_8_ | isosalicylic acid O-glycoside | Phenolic acids | Phenolic acids | A | 1.17 | -2.35 | down |
| 136 | mws0008 | C_9_H_10_O_2_ | Hydrocinnamic acid | Phenolic acids | Phenolic acids | B | 1.12 | -2.76 | down |
| 137 | mws0014 | C_10_H_10_O_4_ | Ferulic acid | Phenolic acids | Phenolic acids | A | 1.17 | 13.32 | up |
| 138 | mws0027 | C_9_H_10_O_5_ | Syringic acid | Phenolic acids | Phenolic acids | A | 1.17 | 14.21 | up |
| 139 | mws0028 | C_8_H_8_O_4_ | Vanillic acid | Phenolic acids | Phenolic acids | A | 1.16 | 3.05 | up |
| 140 | mws0093 | C_10_H_12_O_3_ | Coniferyl alcohol | Phenolic acids | Phenolic acids | B | 1.07 | 2.92 | up |
| 141 | mws0178 | C_16_H_18_O_9_ | Chlorogenic acid | Phenolic acids | Phenolic acids | A | 1.06 | -1.59 | down |
| 142 | mws0180 | C_7_H_6_O_4_ | 2,5-Dihydroxybenzoic acid | Phenolic acids | Phenolic acids | A | 1.17 | 21.01 | up |
| 143 | mws0467 | C_9_H_10_O_3_ | 3-(4-Hydroxyphenyl)-propionic acid | Phenolic acids | Phenolic acids | A | 1.16 | -2.95 | down |
| 144 | mws0628 | C_7_H_6_O_2_ | 4-Hydroxybenzaldehyde | Phenolic acids | Phenolic acids | A | 1.16 | -1.49 | down |
| 145 | mws0885 | C_7_H_6_O_4_ | 2,4-Dihydroxy benzoic acid | Phenolic acids | Phenolic acids | B | 1.17 | 20.90 | up |
| 146 | mws0921 | C_9_H_10_O_2_ | p-Coumaryl alcohol | Phenolic acids | Phenolic acids | A | 1.16 | 2.16 | up |
| 147 | mws0749 | C_7_H_8_O_2_ | 4-Hydroxybenzyl alcohol | Phenolic acids | Phenolic acids | B | 1.16 | -2.54 | down |
| 148 | mws1297 | C_13_H_18_O_6_ | Benzyl β-D-Glucopyranoside | Phenolic acids | Phenolic acids | B | 1.17 | 13.74 | up |
| 149 | mws1354 | C_10_H_10_O_4_ | Trans-ferulic acid | Phenolic acids | Phenolic acids | A | 1.17 | 13.40 | up |
| 150 | mws2212 | C_9_H_8_O_4_ | Caffeic acid | Phenolic acids | Phenolic acids | B | 1.17 | 13.80 | up |
| 151 | mws2213 | C_9_H_8_O_2_ | Cinnamic acid | Phenolic acids | Phenolic acids | A | 1.16 | -4.00 | down |
| 152 | pmb0423 | C_10_H_10_O_4_ | Hydroxy-methoxycinnamate | Phenolic acids | Phenolic acids | B | 1.05 | -1.00 | down |
| 153 | pmb0752 | C_17_H_20_O_9_ | 3-O-Feruloyl quinic acid | Phenolic acids | Phenolic acids | B | 1.13 | 2.72 | up |
| 154 | pmb2871 | C_13_H_16_O_9_ | 2,5-Dihydroxy benzoic acid O-hexside | Phenolic acids | Phenolic acids | A | 1.13 | -1.15 | down |
| 155 | pmb3074 | C_16_H_18_O_8_ | 3-O-p-Coumaroyl quinic acid | Phenolic acids | Phenolic acids | B | 1.14 | 1.19 | up |
| 156 | pme1439 | C_9_H_8_O_3_ | p-Coumaric acid | Phenolic acids | Phenolic acids | A | 1.17 | -3.00 | down |
| 157 | pmn001367 | C_13_H_16_O_9_ | Protocatechuic acid-4-glucoside | Phenolic acids | Phenolic acids | A | 1.16 | -1.02 | down |
| 158 | pmn001419 | C_15_H_18_O_8_ | 1-O-[(E)-p-Cumaroyl]-β-D-glucopyranose | Phenolic acids | Phenolic acids | A | 1.17 | -1.51 | down |
| 159 | pmn001517 | C_15_H_22_O_8_ | 3,4,5-Trimethoxyphenyl-β-D-Glucopyranoside | Phenolic acids | Phenolic acids | B | 1.17 | -2.24 | down |
| 160 | pmn001518 | C_13_H_16_O_10_ | 1-O-Galloyl-β-D-glucose | Phenolic acids | Phenolic acids | B | 1.17 | 9.53 | up |
| 161 | pmn001627 | C_13_H_16_O_10_ | Glucogallin | Phenolic acids | Phenolic acids | B | 1.17 | 12.70 | up |
| 162 | pmn001682 | C_9_H_8_O_4_ | Sorbic acid | Phenolic acids | Phenolic acids | B | 1.17 | 13.08 | up |
| 163 | pmn001710 | C_24_H_26_O_13_ | Rosmarinyl Glucoside | Phenolic acids | Phenolic acids | A | 1.13 | -1.24 | down |
| 164 | pmn001712 | C_22_H_34_O_12_ | 3-Hydroxy-4-isopropylbenzylalcohol 3-glucoside-glucoside | Phenolic acids | Phenolic acids | B | 1.17 | 9.79 | up |
| 165 | pmp000545 | C_16_H_18_O_9_ | 4-Caffeoylquinic acid | Phenolic acids | Phenolic acids | A | 1.17 | -1.88 | down |
| 166 | pmb0492 | C_34_H_37_N_3_O_6_ | N',N'',N'''-p-Coumaroyl-cinnamoyl-caffeoyl spermidine | Alkaloids | Phenolamine | B | 1.17 | -5.24 | down |
| 167 | mws0219 | C_4_H_7_NO_4_ | L-AsparticAcid | Amino acids and derivatives | Amino acids and derivatives | B | 1.17 | 1.60 | up |
| 168 | mws0282 | C_11_H_12_N_2_O_2_ | L-Tryptophan | Amino acids and derivatives | Amino acids and derivatives | A | 1.16 | -5.83 | down |
| 169 | mws0520 | C_11_H_13_NO_4_ | N-Acetyl-L-tyrosine | Amino acids and derivatives | Amino acids and derivatives | B | 1.15 | -1.69 | down |
| 170 | mws0629 | C_13_H_16_N_2_O_5_ | Asp-phe | Amino acids and derivatives | Amino acids and derivatives | B | 1.17 | 13.67 | up |
| 171 | mws0636 | C_18_H_20_N_2_O_3_ | Phe-Phe | Amino acids and derivatives | Amino acids and derivatives | A | 1.04 | 7.82 | up |
| 172 | mws0712 | C_5_H_9_NO_3_ | N-Propionylglycine | Amino acids and derivatives | Amino acids and derivatives | A | 1.16 | -1.26 | down |
| 173 | mws0736 | C_8_H_16_N_2_O_3_ | N-Glycyl-L-leucine | Amino acids and derivatives | Amino acids and derivatives | A | 1.16 | 4.15 | up |
| 174 | mws0890 | C_3_H_7_NO_3_ | Serine | Amino acids and derivatives | Amino acids and derivatives | B | 1.13 | 1.66 | up |
| 175 | mws1050 | C_5_H_9_NO_4_ | O-Acetylserine | Amino acids and derivatives | Amino acids and derivatives | B | 1.17 | -1.40 | down |
| 176 | mws4134 | C_20_H_32_N_6_O_12_S_2_ | Oxidized Glutathione | Amino acids and derivatives | Amino acids and derivatives | B | 1.16 | -4.83 | down |
| 177 | mws4176 | C_12_H_16_N_2_O_3_ | Alanyl-phenylalanine | Amino acids and derivatives | Amino acids and derivatives | A | 1.17 | 5.77 | up |
| 178 | mws5035 | C_15_H_22_N_2_O_3_ | Leucylphenylalanine | Amino acids and derivatives | Amino acids and derivatives | A | 1.17 | 7.47 | up |
| 179 | mws5042 | C_11_H_14_N_2_O_3_ | Glycylphenylalanine | Amino acids and derivatives | Amino acids and derivatives | A | 1.17 | 5.37 | up |
| 180 | pme0014 | C_5_H_9_NO_4_ | L-Glutamic acid | Amino acids and derivatives | Amino acids and derivatives | A | 1.15 | 1.32 | up |
| 181 | pme0124 | C_7_H_12_N_2_O_3_ | Glycyl-L-proline | Amino acids and derivatives | Amino acids and derivatives | B | 1.16 | 2.98 | up |
| 182 | pme0128 | C_6_H_12_N_2_O_3_ | AlanylAlanine | Amino acids and derivatives | Amino acids and derivatives | B | 1.14 | 2.00 | up |
| 183 | pme0137 | C_7_H_12_N_2_O_4_ | N-α-Acetyl-L-glutamine | Amino acids and derivatives | Amino acids and derivatives | B | 1.17 | 13.49 | up |
| 184 | pme0170 | C_8_H_16_N_4_O_3_ | N-α-Acetyl-L-arginine | Amino acids and derivatives | Amino acids and derivatives | B | 1.16 | -2.47 | down |
| 185 | pme0253 | C_8_H_15_NO_3_ | N-Acetyl-L-leucine | Amino acids and derivatives | Amino acids and derivatives | B | 1.17 | -2.25 | down |
| 186 | pme1086 | C_10_H_17_N_3_O_6_S | Glutathione reduced form | Amino acids and derivatives | Amino acids and derivatives | B | 1.14 | 4.38 | up |
| 187 | pme1228 | C_11_H_12_N_2_O_3_ | 5-Hydroxy-L-tryptophan | Amino acids and derivatives | Amino acids and derivatives | B | 1.10 | 1.60 | up |
| 188 | pme2122 | C_5_H_9_N_3_ | Histamine | Amino acids and derivatives | Amino acids and derivatives | B | 1.16 | 1.50 | up |
| 189 | pme2602 | C_3_H_8_NO_6_P | O-Phospho-L-serine | Amino acids and derivatives | Amino acids and derivatives | B | 1.10 | 2.40 | up |
| 190 | pme2743 | C_10_H_11_NO_3_ | N-Phenylacetylglycine | Amino acids and derivatives | Amino acids and derivatives | B | 1.14 | -1.34 | down |
| 191 | pme2853 | C_8_H_15_NO_3_ | Hexanoyl glycine | Amino acids and derivatives | Amino acids and derivatives | B | 1.07 | -1.12 | down |
| 192 | pme2890 | C_8_H_16_N_2_O_4_S_2_ | L-Homocystine | Amino acids and derivatives | Amino acids and derivatives | B | 1.16 | 4.92 | up |
| 193 | pme3193 | C_4_H_7_NO_3_ | N-Acetylglycine | Amino acids and derivatives | Amino acids and derivatives | A | 1.17 | -10.99 | down |

A^a^: The second-level mass spectrometry and RT of the substance are consistent with the database; B^b^: The parameters of Q1, Q3, RT, DP and CE of the substance are consistent with the database.

**Table S5** 14 terpene metabolites in TR by sulfur-fumigation.

| NO. | Index | Formula | Name | Class I | Class II | VIP | Log2FC | Type |
| --- | --- | --- | --- | --- | --- | --- | --- | --- |
| 1 | mws0407 | C_30_H_44_O_7_ | Cucurbitacin D | Terpenoids | Triterpene | 1.17 | -13.86 | down |
| 2 | pmp000896 | C_32_H_46_O_8_ | Cucurbitacin B | Terpenoids | Triterpene | 1.17 | -15.00 | down |
| 3 | pmn001429 | C_32_H_46_O_9_ | Cucurbitacin A | Terpenoids | Triterpene | 1.17 | -3.26 | down |
| 4 | pmn001433 | C_30_H_46_O_7_ | Cucurbitacin F | Terpenoids | Triterpene | 1.17 | -2.22 | down |
| 5 | pmp000898 | C_36_H_54_O_11_ | Deacetoxycucurbitacin B 3-O-glucoside | Terpenoids | Triterpene Saponin | 1.16 | -3.09 | down |
| 6 | pmp000901 | C_38_H_56_O_13_ | Isocucurbitacin B 2-O-glucoside | Terpenoids | Triterpene Saponin | 1.15 | -1.89 | down |
| 7 | pmp000270 | C_30_H_44_O_6_ | Lup-12-en-15α,19β-diol-3,11-dioxo-28-oic acid | Terpenoids | Triterpene | 1.15 | -1.10 | down |
| 8 | Xigua_cub060 | C_32_H_46_O_8_ | 23,24-Dihydro cucurbitacin E | Terpenoids | Triterpene | 1.15 | -2.68 | down |
| 9 | Xigua_mab0406 | C_38_H_56_O_13_ | Cucurbitacin D-aceGlu | Terpenoids | Triterpene Saponin | 1.15 | -2.63 | down |
| 10 | Xigua_mab0543 | C_38_H_56_O_13_ | 23,24-Dihydro cucurbitacin E O-glucoside | Terpenoids | Triterpene Saponin | 1.13 | -2.10 | down |
| 11 | pmp000899 | C_36_H_56_O_12_ | Cucurbitacin F O-glucoside | Terpenoids | Triterpene Saponin | 1.13 | -4.32 | down |
| 12 | pmn001705 | C_30_H_48_O_4_ | 3,24-Dihydroxy-17,21-semiacetal-12(13) oleanolic fruit | Terpenoids | Triterpene | 1.11 | -1.39 | down |
| 13 | Xigua_mab0299 | C_36_H_54_O_12_ | Dihydroisocucurbitacin I-Hex | Terpenoids | Triterpene Saponin | 1.08 | -1.70 | down |
| 14 | Xigua_mab0310 | C_36_H_54_O_12_ | Cucurbitacin D O-glucoside | Terpenoids | Triterpene Saponin | 1.08 | -1.70 | down |
